# RNA‐seq profiling of tubulointerstitial tissue reveals a potential therapeutic role of dual anti‐phosphatase 1 in glomerulonephritis

**DOI:** 10.1111/jcmm.17340

**Published:** 2022-04-29

**Authors:** Sehoon Park, Hajeong Lee, Jeongha Lee, Sangmoon Lee, Semin Cho, Hyeok Huh, Joo Young Kim, Minkyoung Park, Soojin Lee, Yaerim Kim, Murim Choi, Kwon Wook Joo, Yon Su Kim, Seung Hee Yang, Dong Ki Kim

**Affiliations:** ^1^ 37990 Department of Biomedical Sciences Seoul National University College of Medicine Seoul Korea; ^2^ Department of Internal Medicine Armed Forces Capital Hospital Gyeonggi‐do Korea; ^3^ 58927 Department of Internal Medicine Seoul National University Hospital Seoul Korea; ^4^ 58927 Kidney Research Institute Seoul National University Seoul Korea; ^5^ Department of Internal Medicine Uijeongbu Eulji University Medical Center Gyeonggi‐do Korea; ^6^ 37976 Department of Internal Medicine Keimyung University School of Medicine Daegu Korea; ^7^ 37990 Department of Internal Medicine Seoul National University College of Medicine Seoul Korea

**Keywords:** DUSP1, kidney biopsy, microdissection, RNA sequencing, transcriptome

## Abstract

Transcriptome profiling of tubulointerstitial tissue in glomerulonephritis may reveal a potential tubulointerstitial injury‐related biomarker. We profiled manually microdissected tubulointerstitial tissue from biopsy cores of 65 glomerulonephritis cases, including 43 patients with IgA nephropathy, 3 with diabetes mellitus nephropathy, 3 with focal segmental glomerulosclerosis, 3 with lupus nephritis, 4 with membranous nephropathy and 9 with minimal change disease, and additional 22 nephrectomy controls by RNA sequencing. A potential biomarker was selected based on the false discovery rate, and experiments were performed in TNF‐α‐stimulated primary cultured human tubular epithelial cells (hTECs). We identified 3037 genes with low expression and 2852 genes with high expression in the disease samples compared to the controls. Dual‐specificity phosphatase 1 (DUSP1) exhibited universal low expression in various diseases (log2 fold change, −3.87), with the lowest false discovery rate (7.03E‐132). In further experimental validation study, DUSP1 overexpression ameliorated inflammatory markers related to MAP kinase pathways in hTECs, while pharmacologic inhibition of DUSP1 increased these markers. The combination of DUSP1 overexpression with low‐concentration corticosteroid treatment resulted in more potent suppression of inflammation than high‐concentration corticosteroid treatment alone. The profiled transcriptomes provide insights into the pathophysiology of tubulointerstitial injury in kidney diseases and may reveal a potential therapeutic biomarker.

## 1 INTRODUCTION

Glomerulonephritis is one of the major aetiologies of chronic kidney disease.^1^ Both primary glomerulonephritis, such as immunoglobulin A nephropathy (IgAN), and secondary nephropathies, such as diabetic kidney disease, are related to kidney function decline and are major health problems affecting patient prognosis and causing substantial socioeconomic burden.

Many glomerulonephritis diseases share a common tubulointerstitial fibrosis pathway in their advanced state.^2^ As treatment strategies targeting tubulointerstitial changes may reverse kidney function impairment, previous studies have aimed to reveal the pathophysiologic mechanism, such as tumour necrosis factor‐alpha (TNFα) or mitogen‐activated phosphatase kinase (MAPK, p38) related pathways,^3^, ^4^, ^5^, ^6^ active in the tubulointerstitium in various glomerulonephritis.^7^ Certain studies tried to ameliorate the activity of glomerulonephritis targeting molecules related to tubulointerstitial injury.^4^, ^8^ However, a specific treatment strategy targeting tubulointerstitial injury in glomerulonephritis has yet been established, although such intervention may improve response rate and avoid the side effects of conventional immunosuppressive treatment.

Transcriptomic profiling provides the opportunity of unsupervised investigation for the abundance of messenger RNA expression. In particular, high‐throughput RNA sequencing (RNA‐seq) overcomes the previous limitations of microarray techniques, enabling a transcriptome annotation with clearer resolution.^9^, ^10^ Currently, few studies have reported tubulointerstitial transcriptome profiles in diverse kidney diseases, and transcriptome profiling can reveal early pathophysiologic changes before they are reflected in protein expression levels.

In this study, we profiled the transcriptomes of tubulointerstitial tissues, from which glomeruli were removed by microdissection, from patients with certain types of glomerulonephritis. Along with reporting the tubulointerstitial transcriptome of major kidney diseases, we compared the RNA expression profiles with those of nephrectomy controls and investigated whether there is a potential biomarker that is universally changed in the profiled glomerulonephritis diseases. We performed additional experimental validation to determine whether the tubulointerstitial transcriptome profiles may suggest a clinically significant biomarker.

## 2 MATERIALS AND METHODS

### 2.1 Ethical considerations

The institutional review boards of Seoul National University Hospital approved this study and the usage of biospecimens from the National Biobank of Korea (No. H‐1706‐139‐816). The National Biobank of Korea obtained the kidney biopsy samples with the informed consent of the patients. The study was performed in accordance with the principles of the Declaration of Helsinki.

### 2.2 RNA‐seq profiling in patient samples and collection of kidney tissue samples

Biopsy cores from glomerulonephritis patients were obtained from punch biopsies performed between 2010 and 2017 and stored in RNAlater (Qiagen) at −80°C.^10^ We collected samples from 46 patients with IgA nephropathy, 3 with diabetes mellitus nephropathy, 3 with focal segmental glomerulosclerosis, 3 with lupus nephritis, 4 with membranous nephropathy and 9 with minimal change disease, according to sample availability. Patients with profound kidney dysfunction and those with an estimated glomerular filtration rate (eGFR) <30 ml/min/1.73 m^2^ were not considered for this study. To establish the control group, normal cortical tissues were collected from the non‐cancer‐affected cortex of 22 renal carcinoma patients who exhibited no evidence of chronic kidney disease (eGFR <60 ml/min/1.73 m^2^ or detected dipstick albuminuria). These nephrectomy control group samples were also stored in RNA later immediately after surgical excision via the same protocol used for the biopsy core tissues.

### 2.3 Microdissection, RNA isolation and RNA‐seq

The frozen cortical tissues from the nephrectomy controls were initially cut into the size of a biopsy core, and all collected kidney tissues were manually microdissected under a stereomicroscope (Olympus) to remove glomeruli. After thorough removal of glomeruli, total RNA was extracted from the remaining tubulointerstitial tissues using an RNeasy Micro Kit (Qiagen). A TruSeq Stranded Total RNA Library Prep Kit (Illumina) was used to construct cDNA libraries. Paired‐end RNA‐seq was performed on the HiSeq 2500 platform (Illumina), and 100‐base‐pair sequences were obtained as in FASTQ format.

### 2.4 RNA‐seq profiling

FastQC was used for the initial quality control, and no significant contamination or low‐quality sequencing was suspected.^11^ The FASTQ reads were aligned to the human reference genome (GRCh37.p13) using the mapping program Bowtie 2 (version 2.1.0.0).^12^ To quantify gene expression, uniquely mapped reads were counted with featureCounts.^13^ Genes with low expression, defined as a read count of less than 20 in more than half of the studied samples, were disregarded in further analysis.^10^, ^14^ Principal component analysis was used to determine technical outliers, and 3 IgA nephropathy samples were excluded. The final transcriptome profile results of 65 diseased kidney and 22 normal control tubulointerstitial samples were deposited in the National Center for Biotechnology (NCBI) Gene Expression Omnibus (GEO) under the accession number GSE175759 (Edgar et al., 2002).^15^

### 2.5 Differentially expressed genes and enrichment analysis

With the read counts, we modelled a negative binomial distribution, and differentially expressed genes (DEGs) were identified as those with a Benjamini–Hochberg‐adjusted false discovery rate <0.05 with the DESeq2 package.^16^ Gene ontology (GO) term annotation and searches for gene domains or pathways that were enriched or significantly depleted in the tubulointerstitial transcriptome of diseased kidneys were performed with ToppGene Suite, and analyses included the DEGs with an absolute log2 fold change >1 and a false discovery rate <0.05.^17^ The annotated domains with a false discovery rate <0.05 are presented. Heatmaps and correlation plots were drawn with the counts per million reads values of the genes of interest.

### 2.6 Selection of the gene of interest for experimental validation

The gene that showed the greatest difference based on the false discovery rate (i.e. dual‐specificity phosphatase 1, *DUSP1*) was selected as the gene of interest for experimental validation. To ensure that gene expression was universally altered in the profiled kidney diseases, we tested whether the expression level in each disease category was significantly different compared with that in the nephrectomy controls.

### 2.7 Immunohistochemical and immunofluorescence staining

Immunohistochemical staining was performed on independent human kidney tissues. We included samples from patients with major primary glomerulonephritis, specifically 32 patients with IgA nephropathy, 14 with membranous nephropathy and 15 with minimal change disease. In addition, pathology slides from 24 nephrectomy controls were included. No patient had a significantly reduced eGFR (<60 ml/min/1.73 m^2^). We sliced paraffin‐embedded, unstained tissue sections stored at the time of kidney biopsy or nephrectomy into 4‐μm sections. After deparaffinization and rehydration with three incubations in xylene followed by a series of descending concentrations of ethanol, immunohistochemical staining was performed. To block nonspecific background staining, blocking reagent was used. In addition to *DUSP1*, kinases affected by *DUSP1* activity, including p38 and *ERK*, were also targeted in this experiment. Staining with an antibody against *DUSP1*, phospho‐p38 and phospho‐*ERK* (Novus Biologicals) was conducted at 4°C overnight. Images were acquired using a light microscopy system (Leica Microsystems). We quantified the staining results with ImageJ (version 1.8.0., National Institute of Health).

For immunofluorescence staining, deparaffinized tissue slides were stained with a primary antibody against *DUSP1* (Novus Biologicals) followed by secondary antibodies (Alexa Fluor goat anti‐mouse 488 [A1101] and goat anti‐rabbit 555 [A214228]; Thermo Fisher Scientific). For nuclear staining, 4′,6‐diamidino‐2‐phenylindole (DAPI) was used. Confocal microscopy was performed on a TCS SP8 STED CW confocal microscope (Leica Microsystems).

### 2.8 Primary cultured human tubular epithelial cells

We additionally performed an *in vitro* study, and primary cultured human tubular epithelial cells (hTECs) from the unaffected cortex of a nephrectomy control individual were obtained.^18^ After mechanically dissecting the kidney cortices, we isolated tubules with the sieving technique and cultured them for 8 days. We trypsinized the outgrowing cells and collected cultured hTECs via additional sieving. On day 8, the cultured hTECs were counted, and 1 × 10^6^ cells were incubated with Fc receptor blocking reagent (1 μg/ml, BD Bioscience). We identified hTECs by labelling with anti‐CD90 monoclonal antibodies (cat. 12‐0909‐42, Thermo Fischer Scientific) and sorting in a BD FACSCalibur instrument (BD Bioscience). The sorted cells were cultured in a humidified 5% CO_2_ atmosphere at 37°C.

### 2.9 Western blot analysis

With the hTECs, we performed Western blot analysis and evaluated the *DUSP1*‐related molecular pathways with altered *DUSP1* activity. As *DUSP1* suppresses the activity of p38, its target molecules include not only p38 but also the molecules in the downstream c‐Jun (JUN)/c‐Fos (FOS) pathway (including JUN, FOS and c‐Jun N‐terminal kinase [JNK]) and extracellular signal‐regulated kinase (ERK).^19^ In addition, the downstream transcription factor activated protein 1 (AP‐1) was evaluated. We treated hTECs with TNFα to mimic tubulointerstitial inflammation, because evidence indicates that this molecule plays a role in various kidney diseases.^6^, ^20^, ^21^ hTECS were stimulated with TNFα at a concentration of 10 ng/ml for 1 h.

For Western blot analysis, we extracted total protein and separated equal amounts (80 μg) of protein on 10% sodium dodecyl sulphate (SDS)‐polyacrylamide gels and transferred them onto Immobilon‐FL 0.4 μM polyvinylidene difluoride membranes (Millipore). Primary antibodies against β‐actin (Sigma‐Aldrich), DUSP1 (Novus Biologicals), p38 (Cell Signaling Technology), phospho‐p38 (Cell Signaling Technology), JNK (Cell Signaling Technology), phospho‐JNK (Cell Signaling Technology), ERK (Cell Signaling Technology), phospho‐ERK (Cell Signaling Technology), JUN (Cell Signaling Technology), phospho‐JUN (Cell Signaling Technology), FOS (Cell Signaling Technology), phospho‐FOS (Cell Signaling Technology), AP‐1 (MyBioSource) and phospho‐AP1 (MyBioSource) were used. We used an anti‐rabbit IgG secondary antibody (Cell Signaling Technology). Quantification of Western blot band densities was performed with ImageJ, and the relative levels of DUSP1 and phosphoproteins were calculated by dividing these levels by the expression level of β‐actin and the expression levels of the corresponding total forms.

### 2.10 *In vitr* *o* pharmacologic inhibition of DUSP1

BCI is an allosteric inhibitor of DUSP1/6, and its effect on various disease conditions has been shown.^22^, ^23^ We first confirmed the appropriate BCI concentration for the experiment by adding BCI (Millipore) at 1.25 mM, 2.5 mM and 5.0 mM to the culture medium of hTECs for 3 h and then stimulating the hTECs with TNFα. The BCI concentration considered to sufficiently increase p38 expression was used for the main experiment. Next, we tested the effects of pharmacologic inhibition by BCI in cells with no treatment, with BCI treatment only, with TNFα stimulation only, and with TNFα stimulation after treatment with BCI at the determined concentration for 3 h.

### 2.11 *In vitro* adenovirus‐mediated overexpression of DUSP1

We used custom‐manufactured adenovirus for *in vitro* transduction of *DUSP1* (adeno‐*DUSP1*, Sirion Biotech, Germany) to overexpress *DUSP1* in hTECs. We first confirmed the appropriate multiplicity of infection (MOI) for *DUSP1* overexpression by transducing cells with adeno‐DUSP1 at MOIs of 10, 25 and 50 for 24 h and assessing phospho‐p38 and DUSP1 levels. Next, we assessed the relative levels of the target molecules in cells transduced with control adenovirus, transduced with adeno‐*DUSP1*, stimulated with TNFα and stimulated with TNFα after adenovirus‐mediated overexpression of *DUSP1*.

### 2.12 *In vitro* glucocorticoid treatment

As previous studies suggested that glucocorticoids act through *DUSP1* to exert their effects on p38 and related pathways, we further sought to determine whether specifically targeting *DUSP1* may be helpful for reducing the glucocorticoid dosage while maintaining immunosuppressive efficacy in hTECs. First, to confirm the dexamethasone concentration, we assessed phospho‐p38 and DUSP1 levels in hTECs treated with 0.25, 0.5 and 1.0 mM dexamethasone (Sigma‐Aldrich) for 1 h and then stimulated with TNFα. Next, we treated TNFα‐stimulated hTECs with a low concentration and a high concentration of dexamethasone and assessed the relative levels of the target molecules. In addition, hTECs transduced with adeno‐DUSP1 at an MOI of 50 for 24 h were treated with the low concentration of dexamethasone and then stimulated with TNFα.

## 3 RESULTS

### 3.1 Clinical characteristics of the study patients

The clinical characteristics of the profiled individuals are presented in Table 1. The profiled patients exhibited largely preserved kidney function (eGFR ≥60 ml/min/1.73 m^2^); 5 study patients—one with IgA nephropathy, one with diabetic nephropathy, one with focal segmental glomerulosclerosis and two with minimal change disease—had an eGFR <60 ml/min/1.73 m^2^. The lupus nephritis patients had the youngest age range and the highest median eGFR value, while the minimal change disease and membranous nephropathy patients had a relatively high median urine protein‐to‐creatinine ratio (above 10 g/g). A total of 3 kidney disease patients—2 minimal change disease patients and one focal segmental glomerulosclerosis patient—had been treated with immunosuppressants before kidney biopsy.

**TABLE 1:** Baseline characteristics of the study population

	Control	IgA nephropathy	Minimal change disease	Membranous nephropathy	Focal segmental glomerulosclerosis	Diabetic nephropathy	Lupus nephritis
Number of individuals	22	43	9	4	3	3	3
Age (years)	55 [49;60]	42 [32;49]	61 [38;75]	63 [57;68]	68 [54;69]	61 [57;63]	30 [25;32]
Sex							
Female	8 (36.4%)	22 (51.2%)	5 (55.6%)	1 (25.0%)	1 (33.3%)	3 (100.0%)	2 (66.7%)
Male	14 (63.6%)	21 (48.8%)	4 (44.4%)	3 (75.0%)	2 (66.7%)	0 (0.0%)	1 (33.3%)
Body mass index (kg/m^2^)	24.4 [22.8; 25.5]	22.5 [20.5; 24.5]	25.7 [22.8; 26.5]	25.0 [24.6;25.4]	22.8 [22.4; 24.5]	24.4 [22.8; 24.6]	23.3 [21.2; 24.4]
Creatinine (mg/dL)	0.8 [0.7; 0.9]	0.9 [0.8; 1.0]	0.8 [0.7; 1.0]	0.7 [0.5; 0.8]	0.7 [0.7; 1.2]	0.7 [0.7; 0.9]	0.7 [0.5; 0.7]
eGFR (ml/min/1.73 m^2^)	96.0 [86.0; 105.0]	88.2 [75.3; 105.8]	78.1 [61.9; 106.9]	97.5 [89.4;107.8]	81.5 [61.0; 100.7]	84.2 [70.9; 91.9]	126.2 [126.2;133.0]
Urine protein‐to‐creatinine ratio (g/g)	1.0 [1.0; 1.0]	0.9 [0.5; 1.7]	11.4 [9.7; 15.7]	14.2 [6.1;19.6]	2.7 [2.6; 6.0]	3.3 [2.6; 5.2]	5.1 [3.1; 6.2]
Hypertension	9 (40.9%)	14 (32.6%)	2 (22.2%)	2 (50.0%)	3 (100.0%)	1 (33.3%)	1 (33.3%)
Diabetes mellitus	1 (4.5%)	2 (4.7%)	0 (0.0%)	0 (0.0%)	1 (33.3%)	3 (100.0%)	0 (0.0%)
Immunosuppressant use before biopsy	0 (0%)	0 (0%)	2 (22.2%)	0 (0%)	1 (33.3%)	0 (0%)	1 (33.3%)

Abbreviation: eGFR, estimated glomerular filtration rate.

### 3.2 Explanatory analysis results

In our RNA‐seq profiling data, the samples showed a >90% mapping rate to Ensembl‐annotated exons with a median of 34,032,131 (interquartile range, 31,211,550‐37,958,876) mapped reads. After filtering out the genes with low expression, 16,164 genes were included in the downstream analysis. The normalized counts per million reads values were relatively low for glomerulus‐specific genes, including nephrin (*NPHS1)* and podocin (*NPHS2*), but were relatively high for tubule‐specific genes, including aquaporin 1 (*AQP1*), uromodulin (*UMOD*), solute carrier family 12 member 3 (*SLC12A3*) and *AQP2* (Figure S1). Specifically, these results implied that the gene expression profile of the tubulointerstitium was successfully acquired via our microdissection technique with minimal contamination by glomeruli. The global patterns of gene expression were investigated by principal component analysis, and some overlap was noted between the kidney disease group and the nephrectomy control group based on the first and second principal components.

### 3.3 Differentially expressed genes

**FIGURE 1:**
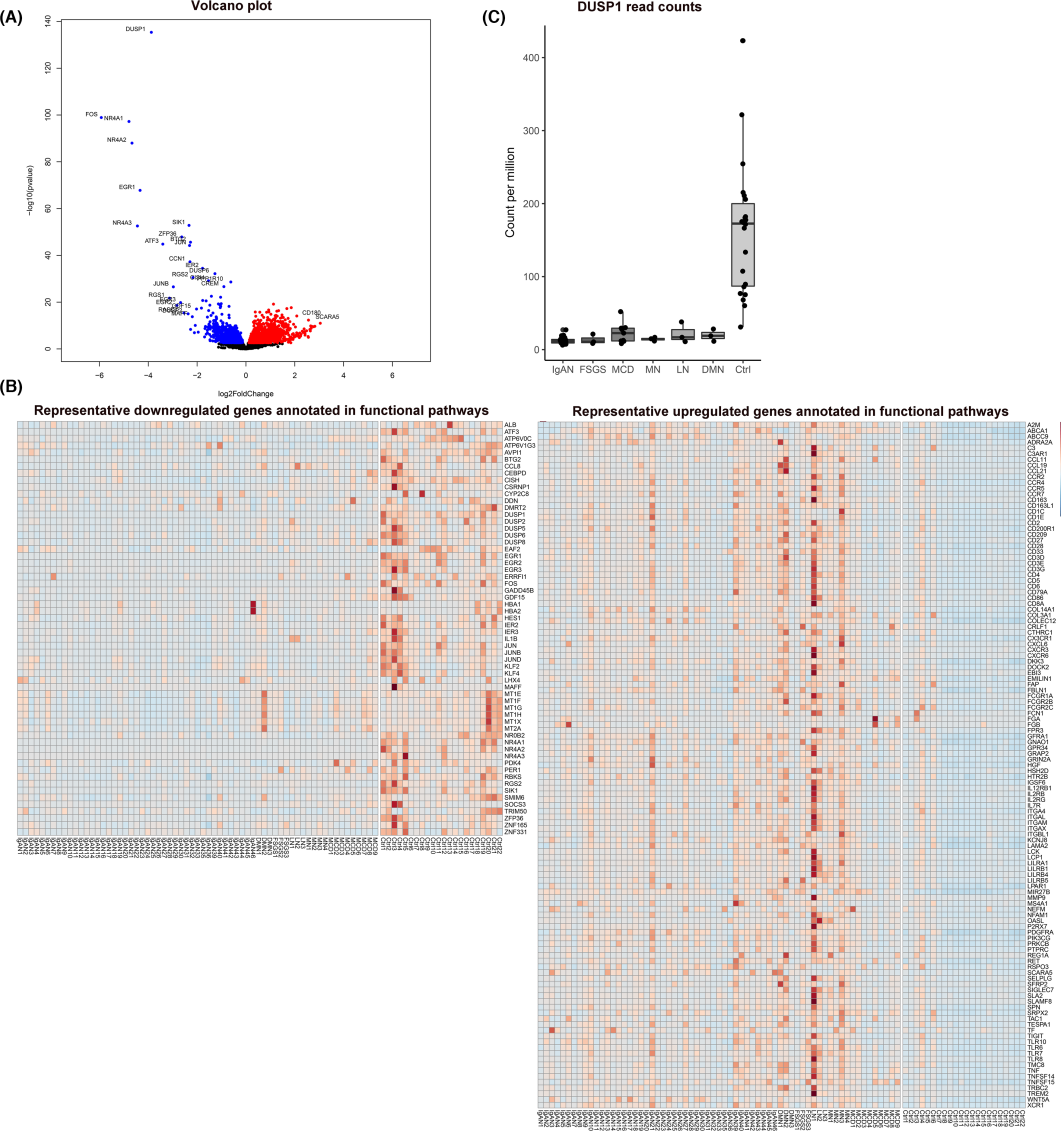
Differentially expressed genes and representative gene expression levels. (A) Volcano plot showing the differentially expressed genes. The differentially expressed genes with a false discovery rate <0.05 are coloured blue (for genes with low expression) and red (for genes with high expression). (B) Representative gene expression levels in each sample. The plotted genes were annotated by gene ontology analysis. Red indicates relatively high expression, and blue indicates relatively low expression, graded in 10 ordinal categories. The representative genes were selected based on the gene ontology term annotation findings and the genes that most commonly annotated are presented in the figure. (C) DUSP1 read counts in the study samples were determined to confirm that the expression level was generally reduced in patients with kidney disease

We identified 3037 genes with low expression and 2852 genes with high expression with a false discovery rate <0.05 (Table S1 and Figure 1A). Among these genes, 142 genes with low expression showed a log2 fold change value <−1, and 383 genes with high expression showed a log2 fold change value ≥1. The significantly downregulated genes with the greatest absolute log2 fold change rates were *FOS*, nuclear receptor subfamily 4 group A member 1 (*NR4A1*), *NR4A2*, *NR4A3*, early growth response 1 (*EGR1*) and *DUSP1*, and *DUSP1* showed the lowest false discovery rate (7.03E‐132). The significantly upregulated genes with the greatest log2 fold change were immunoglobulin heavy chain variable (*IGHV*) genes. The overall false discovery rates for the upregulated genes were larger than those for the downregulated genes, and ATP binding cassette subfamily G member 1 (ABCG1) had the lowest false discovery rate (4.10E‐17).

### 3.4 Gene ontology enrichment analysis

There were 844 gene ontology domains (46 molecular function, 749 biological processes, 49 cellular components) annotated by the highly expressed DEGs and 250 domains (33 molecular function, 215 biological process, 2 cellular component) by the lowly expressed DEGs, respectively (Table S2). Regarding the genes with low expression, notable significantly enriched gene ontology molecular function domains included MAP kinase or protein kinase pathways, transcription factor activity‐related pathways and pathways related to glucocorticoid receptor activity. Diverse biological process gene ontology terms were identified, and notable terms included cellular response to stimulus and negative regulation of MAP kinase or related pathways. Transcription factor AP‐1 complex (GO:0035976) and nuclear chromatin (GO:0000790) were the cellular component gene ontology terms that were enriched with the significantly downregulated genes with log2 fold change <−1.

For the highly expressed genes, chemokine or cytokine activity‐related pathways were notable molecular function gene ontology terms identified by annotation. The most significant enrichment was identified in terms related to immune response processes. Regarding cellular component gene ontology terms, cell membrane‐ or immunoglobulin‐related pathways were the primary pathways enriched with the highly expressed genes.

The expression levels of the representative genes included in the gene ontology annotations in each sample are presented in Figure 1B.

### 3.5 Selection of the target gene

For further experimental validation, we selected *DUSP1* as the target gene of interest, considering its significance level in our DEG analysis results (*p* = 7.03E‐132, log 2‐fold change −3.87). In addition, *DUSP1* appeared in a large proportion of the gene ontology annotation results (10/33 molecular function terms, 81/215 biological process terms), and the MAP kinase pathway, which is the direct target of *DUSP1*, was notably identified to be significantly downregulated in the profiled diseased kidney tubulointerstitial tissues.

When we assessed the expression of *DUSP1* according to specific kidney disease types, all categories of profiled diseases showed significantly lower expression of *DUSP1* than the nephrectomy controls (Figure 1C).

### 3.6 Immunohistochemical and immunofluorescence staining

**FIGURE 2:**
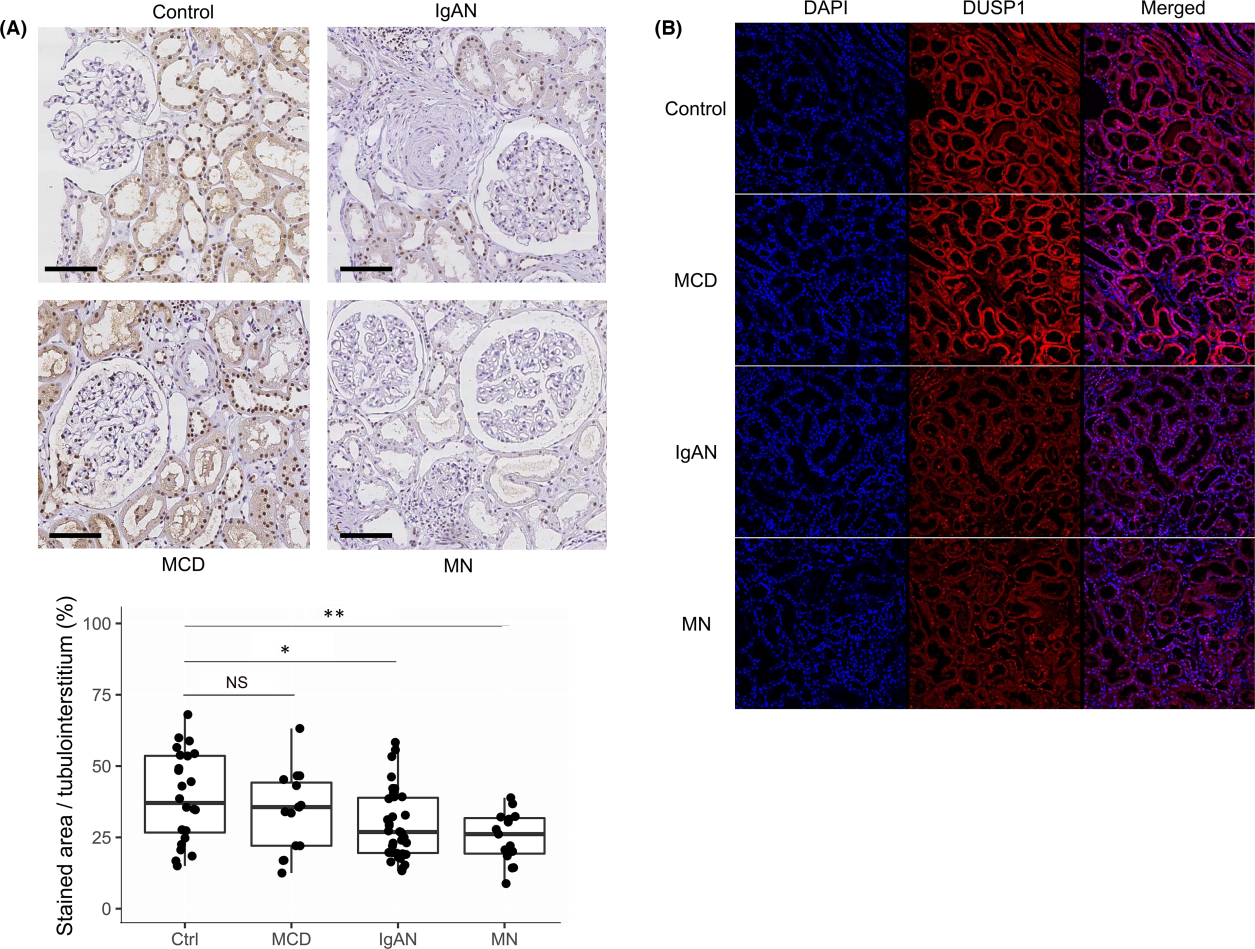
Immunohistochemical and immunofluorescence staining results for DUSP1. (A) Representative immunohistochemical staining results for DUSP1 in samples from controls and patients with IgAN, minimal change disease (MCD) or membranous nephropathy (MN). The expression of DUSP1 was significantly lower in IgA nephropathy and membranous nephropathy samples than in control samples, but this pattern was not observed for minimal change disease samples. ‘NS’ indicates a nonsignificant difference, ‘*’indicates *p* < 0.05 and ‘**’ indicates *p* < 0.01. (B) Immunofluorescence staining showed findings similar to those of immunohistochemical staining, as the expression of DUSP1 was markedly lower in IgA nephropathy and membranous nephropathy samples than in control samples

Immunohistochemical staining of samples from patients with IgA nephropathy, membranous nephropathy or minimal change disease and samples from nephrectomy controls indicated that the protein expression level of DUSP1 was significantly lower in the IgA nephropathy and membranous nephropathy samples than in the nephrectomy control samples (Figure 2A). However, in samples from patients with minimal change disease, no apparent difference was observed.

Immunofluorescence staining revealed similar findings, as DUSP1 expression was prominently reduced in samples from patients with IgA nephropathy or membranous nephropathy, while samples from patients with minimal change disease showed protein expression levels similar to those in samples from controls (Figure 2B).

**FIGURE 3:**
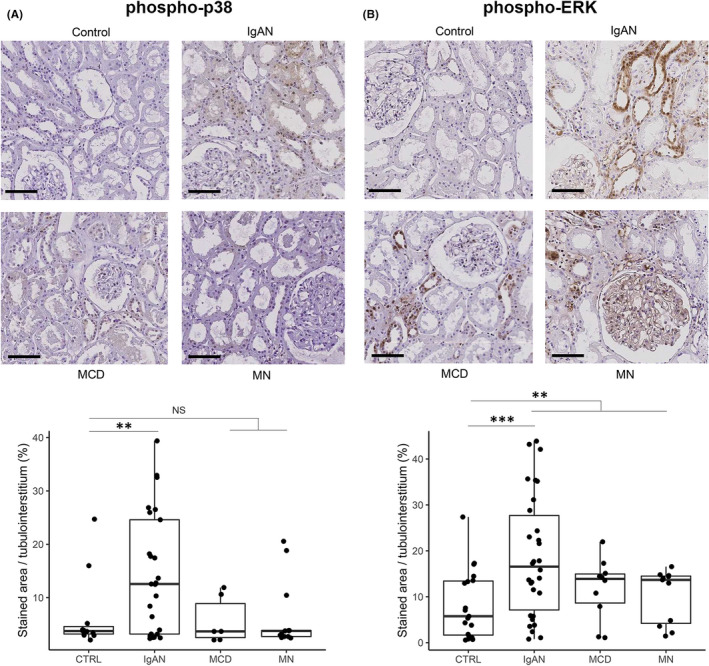
Immunohistochemical and immunofluorescence staining results for phospho‐p38 and phospho‐ERK. (A) Representative immunohistochemical staining results for phospho‐p38. Due to sample availability, staining for phospho‐p38 was performed for 25 IgAN, 13 membranous nephropathy, 6 minimal change disease, 11 membranous nephropathy and 11 control samples, respectively. Expression of phospho‐p38 was significantly higher in IgA nephropathy samples than in control cases, but this pattern was not observed for minimal change disease or membranous nephropathy. (B) Representative immunohistochemical staining results for phospho‐ERK. Staining for phospho‐ERK was performed for 30 IgAN, 10 minimal change disease, 11 membranous nephropathy and 19 control samples, respectively. Expression of phosphor‐ERK was significantly higher in glomerulonephritis cases than in controls, particularly for IgA nephropathy samples. ‘NS’ indicates a nonsignificant difference, ‘**’ indicates *p* < 0.01 and ‘***’ indicates *p* < 0.001

When downstream molecules were assessed, expression of phospho‐p38 was significantly higher in IgA nephropathy samples than in control cases (Figure 3A), but this pattern was not observed for minimal change disease or membranous nephropathy. The expression of phosphor‐ERK was significantly higher in glomerulonephritis cases than in controls, particularly for IgA nephropathy samples (Figure 3B).

### 3.7 *In vitro* experimental results

We further assessed markers of inflammation‐related mechanisms associated with the MAP kinase pathway, including the expression of JNK, ERK, JUN, FOS and AP1 along with the modification of DUSP1.

**FIGURE 4:**
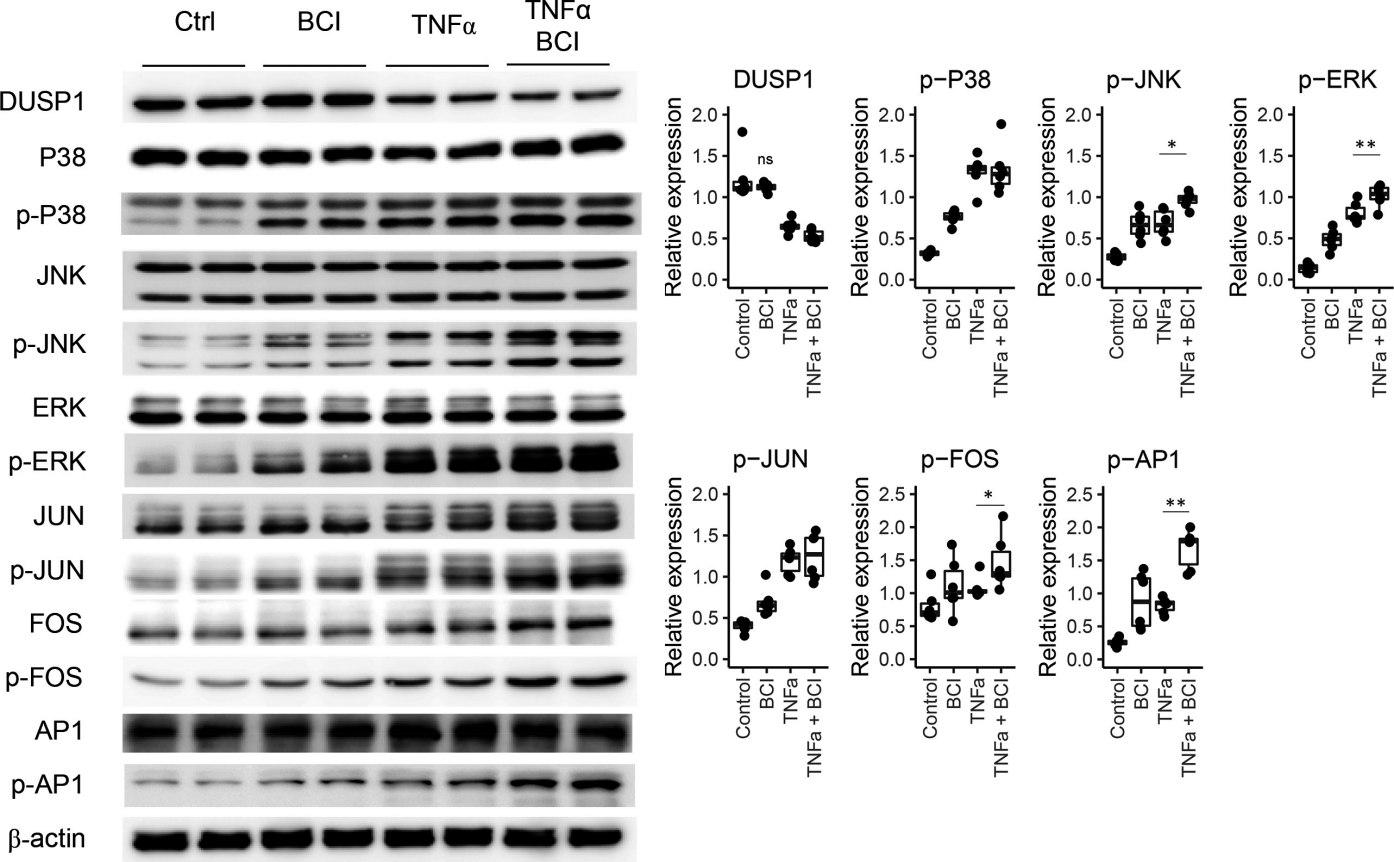
*In vitro* Western blot results for inhibition of DUSP1. DUSP1 and inflammatory molecules in untreated human tubular epithelial cells (hTECs), hTECs treated with 5.0 mM BCI for 3 h, hTECs stimulated with 10 ng/ml TNFα for 1 h, and hTECs treated with BCI and then stimulated with TNFα were analysed by Western blotting. All experiments were performed in triplicate. ‘ns’ indicates a nonsignificant difference, ‘*’ indicates *p* < 0.05 and ‘**’ indicates *p* < 0.01. When comparisons were performed with groups other than the control group, lines were drawn to indicate the groups that were compared

Pharmacologic inhibition of DUSP1/6 with 5 mM BCI (Figure S2) was significantly associated with an increase in the level of phospho‐p38. However, as BCI is an allosteric inhibitor, the expression level of DUSP1 changed only minimally with the addition of BCI. When we evaluated the effect of BCI under various conditions (Figure 4), the effects of DUSP1/6 inhibition with BCI alone on the induction of target inflammatory molecules were similar to those of TNFα stimulation. When TNFα stimulation followed BCI treatment, the levels of phospho‐JNK, phospho‐ERK, phospho‐FOS and phospho‐AP1 were further increased.

**FIGURE 5:**
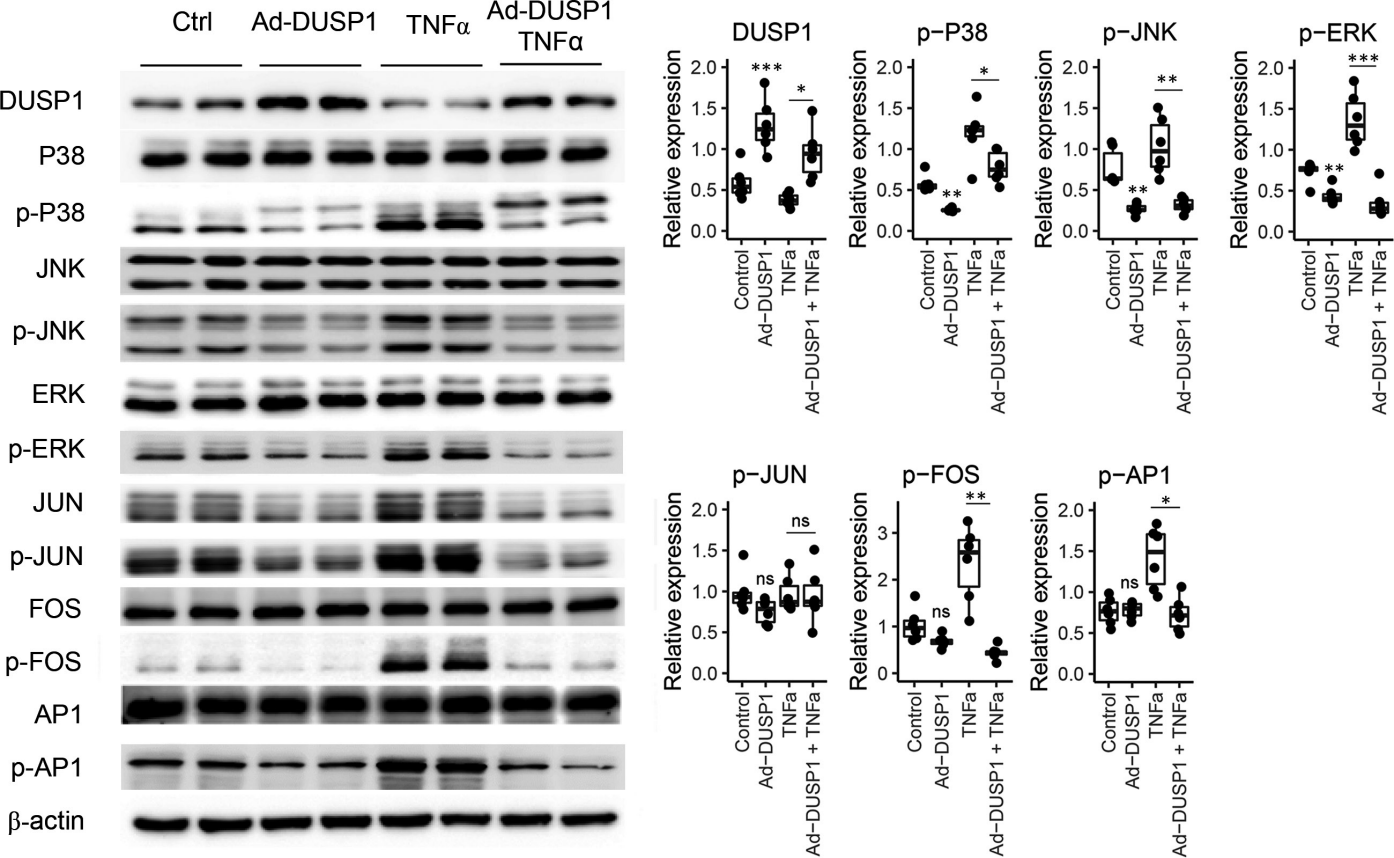
In vitro Western blot results for DUSP1 overexpression. The experiment was performed in human tubular epithelial cells (hTECs) transfected with control adenovirus, hTECs transduced with DUSP1‐adenovirus at 50 MOI, hTECs stimulated with TNFα, and hTECs transduced with DUSP1‐adenovirus and then stimulated with TNFα. All experiments were performed in triplicate. ‘ns’ indicates a nonsignificant difference, ‘*’ indicates *p* < 0.05, ‘**’ indicates *p* < 0.01, and ‘***’ indicates *p* < 0.001. When comparisons were performed with groups other than the control group, lines were drawn to indicate the groups that were compared

On the other hand, adenovirus‐mediated *DUSP1* overexpression at an MOI of 50, which was considered to induce sufficient *DUSP1* overexpression (Figure S3), reduced the levels of phospho‐p38, phospho‐JNK and phospho‐ERK compared with those in hTECs transduced with control adenovirus (Figure 5). Furthermore, when hTECs were transduced with adeno‐*DUSP1* and stimulated with TNFα, the levels of most inflammatory molecules were markedly reduced compared with those in hTECs stimulated with TNFα alone.

**FIGURE 6:**
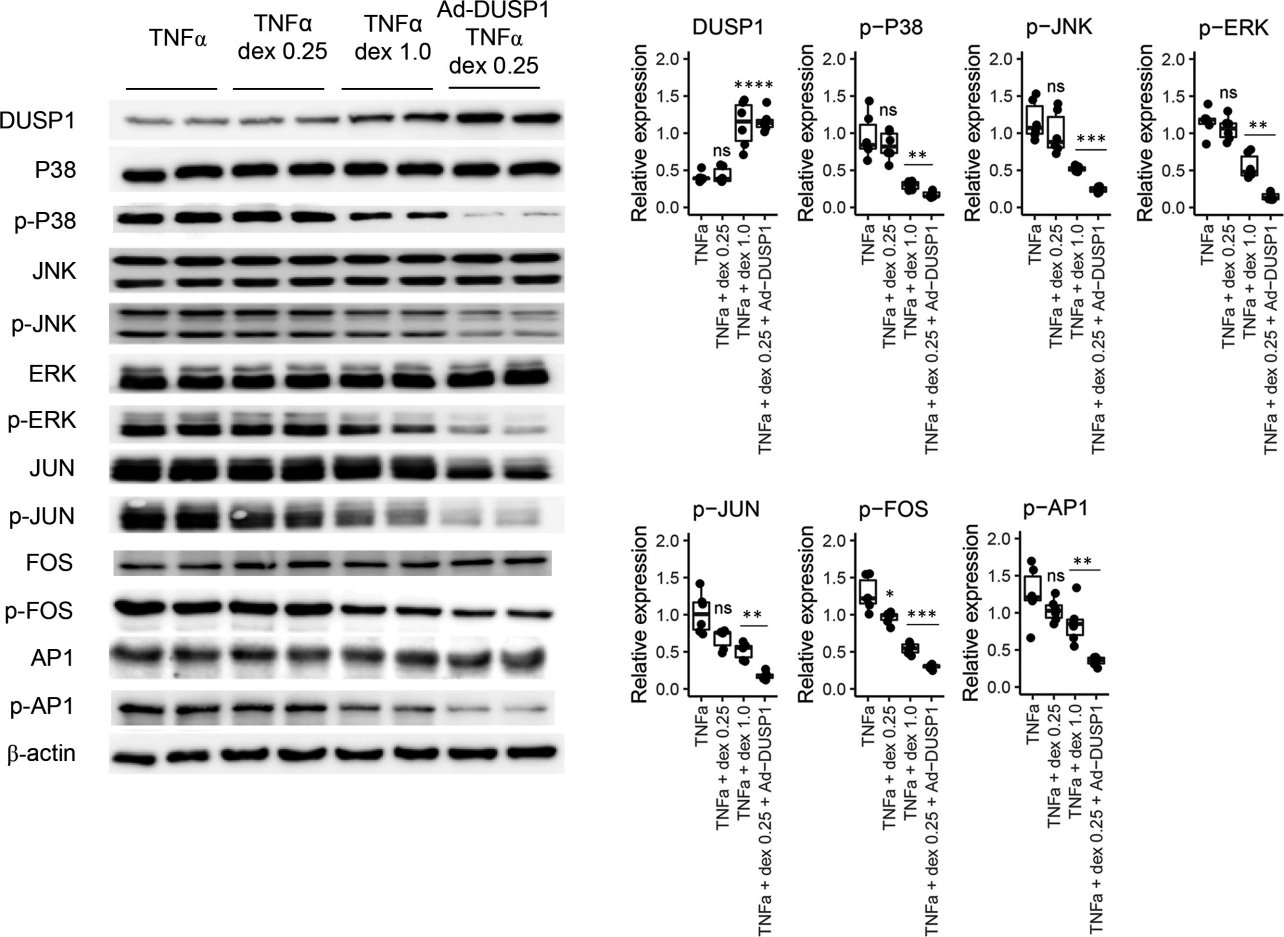
*In vitro* Western blot results for DUSP1 overexpression with addition of corticosteroid treatment. The experiment was performed in human tubular epithelial cells (hTECs) transduced with control adenovirus and stimulated with TNFα, in control adenovirus‐transduced hTECs stimulated with TNFα and treated with dexamethasone (0.25 mM), in control adenovirus‐transduced hTECs stimulated with TNFα and treated with dexamethasone (1.0 mM), and in DUSP1‐adenovirus‐transduced hTECs stimulated with TNFα and treated with dexamethasone (0.25 mM). All experiments were performed in triplicate. ‘ns’ indicates a nonsignificant difference, ‘*’ indicates *p* < 0.05, ‘**’ indicates *p* < 0.01, and ‘***’ indicates *p* < 0.001. When comparisons were performed with groups other than the control group, lines were drawn to indicate the groups that were compared

We further aimed to assess whether modification of DUSP1 may be a potential corticosteroid‐preserving anti‐inflammatory strategy by comparing the effects of DUSP1 overexpression and dexamethasone treatment on stimulated hTECs. In the experiment to determine the appropriate corticosteroid concentration, treatment with a low concentration (0.25 mM, Figure S4) of dexamethasone did not result in significant reductions in the levels of phospho‐p38, phospho‐JNK and phospho‐ERK or a change in DUSP1 expression but did decrease the levels of phospho‐JUN, phospho‐FOS and phospho‐AP1 (Figure 6). In contrast, treatment with a high concentration (1.0 mM) of dexamethasone decreased the levels of certain inflammatory molecules and increased the expression of DUSP1. When hTECs overexpressing DUSP1 were treated with 0.25 mM dexamethasone, the DUSP1 expression level was similar to that in hTECs treated with the high concentration of dexamethasone, and the levels of the target inflammatory molecules were significantly reduced even compared to those after 1.0 mM dexamethasone treatment.

## 4 DISCUSSION

In this study, we performed transcriptome profiling of the tubulointerstitium in various glomerulonephritis diseases by RNA‐seq. The expression levels of various genes were altered in the diseased tubulointerstitium compared to the normal cortex, and the activity of relevant inflammation‐related pathways was altered. Among the DEGs, we performed experimental studies with *DUSP1*, which was universally expressed at low levels in injured kidney tubulointerstitial tissues in various kidney diseases. The *in vitro* experimental results suggested that the modification of *DUSP1* expression is a potential therapeutic strategy for renal tubulointerstitial injury.

High‐throughput sequencing can allow unsupervised transcriptome profiling and can provide novel insights regarding a disease's pathophysiology. In nephrology, RNA‐seq profiling has revealed potential biomarkers for and pathologic pathways mediating kidney diseases.^9^, ^10^ Along with recent advances in novel transcriptome profiling approaches, including single‐cell sequencing techniques,^24^ the understanding of the kidney transcriptome and cellular pathophysiology is being expanded. RNA‐seq of microdissected bulk tissue has some advantages, as it can allow profiling the kidney by microstructure, and the results are less biased by background noise than those obtainable by previous microarray techniques. Compared with the recently advanced single‐cell sequencing technique, RNA‐seq of microdissected bulk tissue can allow profiling of the target tissue even in small kidney biopsy cores; in addition, the major limitation in applying single‐cell sequencing for various kidney diseases is that only a small number of viable cells can be obtained via punch biopsy. In this study, we profiled the tubulointerstitial transcriptome of various kidney diseases by RNA‐seq with a relatively large number of biopsy cores from patients with various types of kidney disease. The identified DEGs and gene ontology analysis results showed relevant findings, and the pathways related to inflammatory processes were altered in diseased tubulointerstitium. One strength of the current study is that the results are freely available online; thus, a future study may investigate the profiling results to search for additional target biomarkers in the kidney tubulointerstitium. Our experiments with *DUSP1* suggested that the transcriptome profiling results may lead to the identification of a potential biomarker for kidney diseases. Furthermore, unlike the current study, a future study may investigate a disease‐specific transcriptome within a single disease category. Alternatively, the current study may serve as an external validation set for other research regarding kidney tubulointerstitial pathophysiology.

Based on the pathway annotation results and false discovery rates, DUSP1, which downregulates the MAP kinase pathway, was selected as the target biomarker for further validation. Activation of the MAP kinase pathway is a core mechanism mediating various glomerulonephritis.^3^, ^5^, ^8^, ^25^ In our study, the expression of DUSP1 was generally reduced in various types of glomerulonephritis, which may promote upregulation of the MAP kinase pathway and related inflammatory signalling. The role of DUSP1 has been suggested in few studies regarding kidney diseases, and previous experimental studies reported that DUSP1 ameliorates diabetic kidney diseases.^4^, ^26^ As both downregulation and upregulation of DUSP1 resulted in altered inflammatory signalling activity in our study, DUSP1 in the kidney tubulointerstitium may be a potential target to reduce inflammation and tubulointerstitial injury.

The promoter region of the DUSP1 gene contains binding sites for AP1, nuclear factor‐κB, cAMP response element‐binding protein and glucocorticoid receptor. Previous studies have focused on the observation that the effects of glucocorticoids are mediated by DUSP1, as the binding of glucocorticoids to glucocorticoid receptors induces DUSP1 transcription, which is the major mechanism further downregulating inflammatory pathways.^27^ In particular, the role of DUSP1 has been emphasized in airway inflammatory diseases (e.g. asthma or chronic obstructive pulmonary diseases), where glucocorticoids are the primary choice of treatment for acute exacerbated states.^28^ As targeting DUSP1 has shown anti‐inflammatory effects in airway cells and *in vivo* models, DUSP1 has been suggested to be a potential therapeutic target for steroid‐resistant cases.^29^, ^30^, ^31^ Regarding glomerulonephritis, glucocorticoids are also a widely used treatment strategy for various inflammation‐related conditions. Glucocorticoids have limitations because of side effects due to their broad effect on diverse systems; thus, modification of DUSP1 expression may be an alternative treatment strategy with limited use of steroids, as suggested by our *in vitro* experimental results. This strategy would be particularly important in diabetic kidney diseases, in which the role of DUSP1 has also been identified by *in vivo* experiments,^26^ and direct modification of DUSP1 may reduce inflammatory injury while avoiding the metabolic side effects of glucocorticoids. Alternatively, for glomerulonephritis for which glucocorticoid treatment has limited efficacy, directly targeting DUSP1 may be a potential strategy to prevent tubulointerstitial injury because of its potency. Thus, the results of our study support future studies to test the efficacy of targeting DUSP1 in various glomerulonephritis and researchers may particularly focus on the validation of the benefits of DUSP1 restoration by *in vivo* experiments.

The current study has several limitations and points to consider. First, although profiling was conducted on a relatively large number of samples acquired from biopsy cores, many samples were from patients with IgA nephropathy, as this disease is the most prevalent primary glomerulonephritis diagnosed by renal biopsy. Furthermore, selection bias for other disease types remains because of the small number of included samples. As immunohistochemistry results for downstream molecules showed some different findings according to specific glomerulonephritis diseases, future studies utilizing our transcriptome profiling results should consider that mRNA expression may differ across disease types. Second, the protein expression level of DUSP1 in samples from patients with minimal change disease was not significantly different from that in samples from controls as assessed by immunohistochemical staining, although the RNA‐seq profiles showed prominently lower *DUSP1* mRNA expression levels. This discrepancy may imply that as tubular injury might have been milder in the patients with minimal change disease than in those with IgA nephropathy or membranous nephropathy, a relatively small difference might not have been evident by immunohistochemical staining, and RNA‐seq might have detected the early changes in mRNA expression. Third, as the study was performed in a single centre including an East Asian population, the generalizability of the results should be expanded by future studies.

In conclusion, transcriptome profiling of tubulointerstitial tissue from patients with various glomerulonephritis revealed notable findings that may provide insights into pathophysiologic mechanisms. Future studies may consider the utility of *DUSP1* as a potential treatment target for preventing inflammation‐related tubulointerstitial injury.

## CONFLICT OF INTEREST

The authors declare no conflicts of interest.

## AUTHOR CONTRIBUTIONS

**Sehoon Park:** Conceptualization (equal); Data curation (equal); Formal analysis (equal); Funding acquisition (equal); Investigation (equal); Methodology (equal); Resources (equal); Software (equal); Visualization (equal); Writing – original draft (lead); Writing – review & editing (equal). **Hajeong Lee:** Conceptualization (equal); Data curation (equal); Formal analysis (equal); Methodology (equal); Project administration (equal); Resources (equal); Software (equal); Supervision (equal); Visualization (equal); Writing – original draft (lead); Writing – review & editing (equal). **Jeongha Lee:** Data curation (equal); Formal analysis (equal); Investigation (equal); Methodology (equal); Resources (equal); Software (equal); Writing – original draft (equal). **Sangmoon Lee:** Conceptualization (equal); Data curation (equal); Formal analysis (equal); Investigation (equal); Methodology (equal); Resources (equal); Software (equal); Writing – original draft (equal). **Semin Cho:** Data curation (equal); Formal analysis (equal); Funding acquisition (equal); Investigation (equal); Methodology (equal); Writing – original draft (equal). **Hyeok Huh:** Data curation (equal); Formal analysis (equal); Methodology (equal); Resources (equal); Writing – original draft (supporting). **Joo Young Kim:** Formal analysis (equal); Funding acquisition (equal); Investigation (equal); Resources (equal). **Minkyoung Park:** Data curation (equal); Formal analysis (equal); Investigation (equal); Resources (equal); Software (equal); Validation (equal); Writing – original draft (equal). **Soojin Lee:** Conceptualization (equal); Data curation (equal); Formal analysis (equal); Investigation (equal); Resources (equal); Writing – original draft (equal). **Yaerim Kim:** Conceptualization (equal); Data curation (equal); Investigation (equal); Methodology (equal); Resources (equal); Writing – original draft (equal). **Murim Choi:** Conceptualization (equal); Data curation (equal); Methodology (equal); Project administration (equal); Resources (equal); Software (equal); Supervision (equal); Validation (equal); Visualization (equal); Writing – original draft (equal); Writing – review & editing (equal). **Kwon Wook Joo:** Data curation (equal); Formal analysis (equal); Funding acquisition (equal); Investigation (equal); Methodology (equal); Resources (equal); Software (equal); Supervision (equal); Writing – original draft (equal); Writing – review & editing (equal). **Yon Su Kim:** Data curation (equal); Formal analysis (equal); Funding acquisition (equal); Methodology (equal); Project administration (equal); Resources (equal); Writing – original draft (equal); Writing – review & editing (equal). **Seung Hee Yang:** Conceptualization (equal); Data curation (equal); Formal analysis (equal); Investigation (equal); Methodology (equal); Project administration (equal); Resources (equal); Software (equal); Supervision (equal); Validation (equal); Visualization (equal); Writing – original draft (equal); Writing – review & editing (equal). **Dong Ki Kim:** Conceptualization (equal); Data curation (equal); Formal analysis (equal); Funding acquisition (equal); Investigation (equal); Methodology (equal); Project administration (equal); Resources (equal); Software (equal); Supervision (equal); Validation (equal); Visualization (equal); Writing – original draft (lead); Writing – review & editing (lead).

## Supporting information

Figure S1‐S6

Table S1

Table S2

Supplementary Material

## Data Availability

The final transcriptome profile results of 65 diseased kidneys and 22 normal control tubulointerstitial samples were deposited in the National Center for Biotechnology (NCBI) Gene Expression Omnibus (GEO) under the accession number GSE175759. Other data of experimental studies will be made available by the corresponding authors upon a reasonable request.
