# Supplementary material for: RNA‐seq profiling of tubulointerstitial tissue reveals a potential therapeutic role of dual anti‐phosphatase 1 in glomerulonephritis
Source: J Cell Mol Med. 2022 Apr 29;26(12):3364–77. doi: 10.1111/jcmm.17340 (PMC9189340; doi:10.1111/jcmm.17340)
Supplement: Supplementary file 1 — Figure S1‐S6 [file JCMM-26-3364-s002.pptx]

## Slide 1
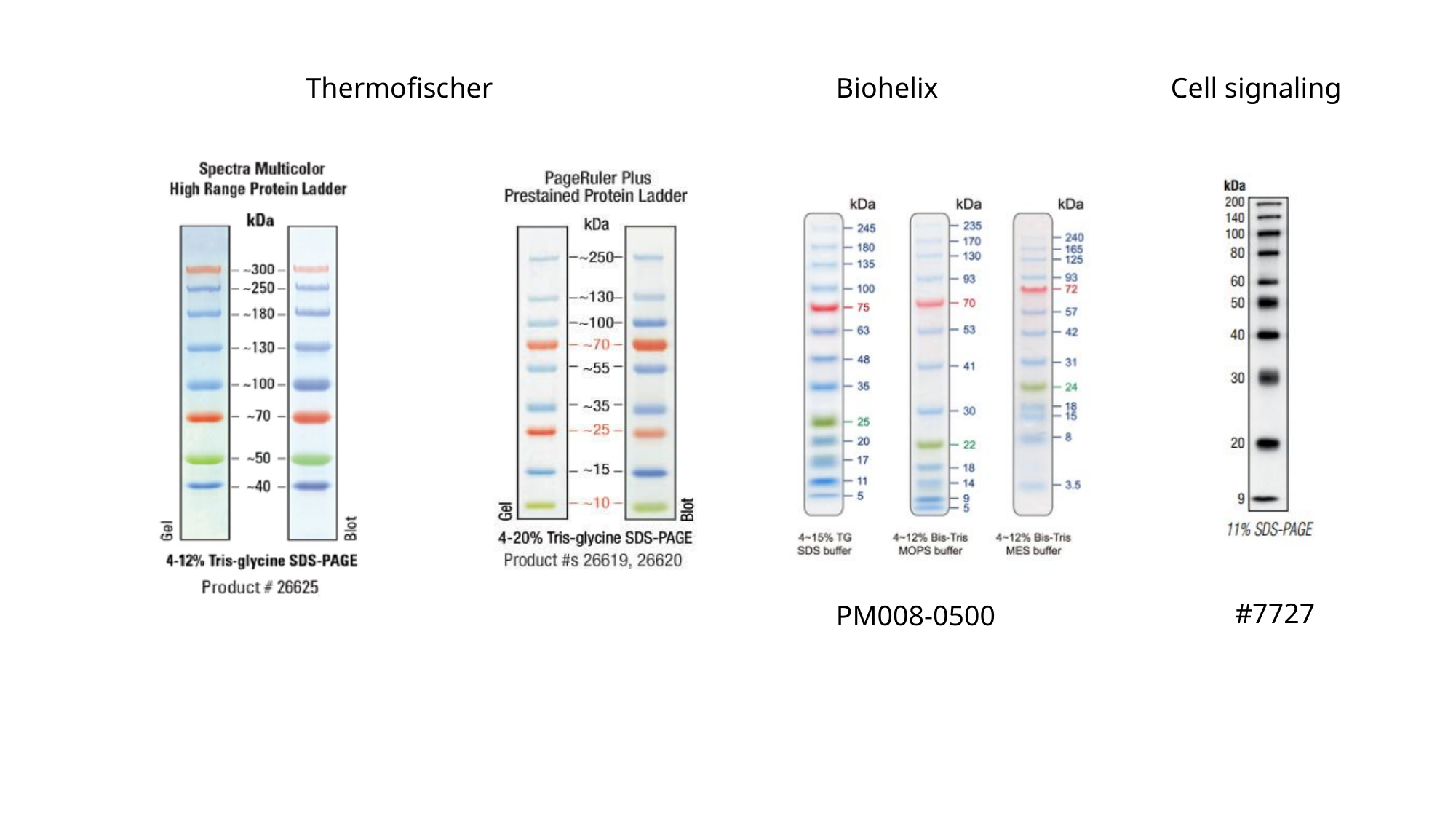

Thermofischer
Biohelix
Cell signaling
#7727
PM008-0500

## Slide 2
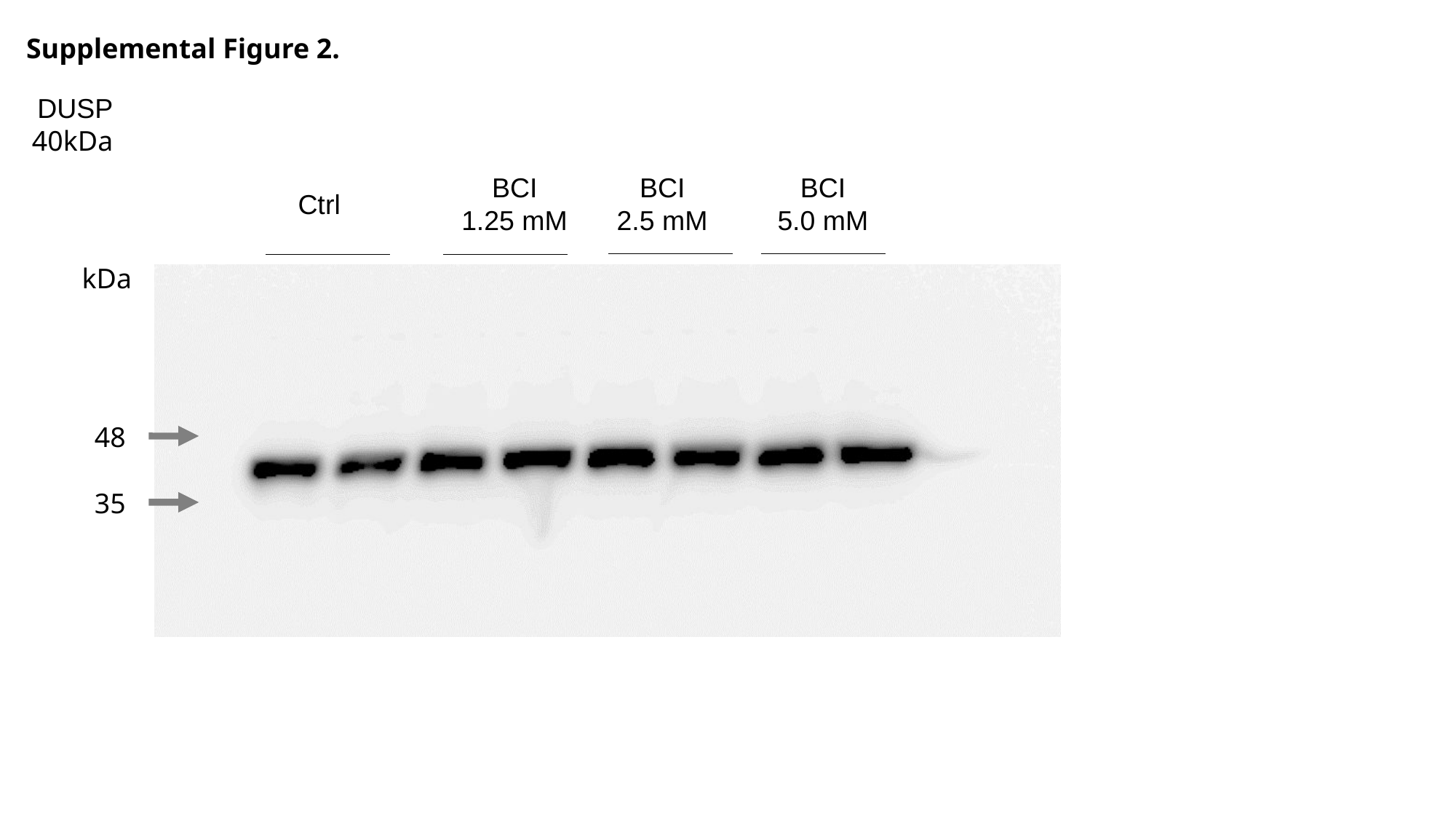

Supplemental Figure 2.
DUSP
40kDa
BCI
5.0 mM
BCI
1.25 mM
BCI
2.5 mM
Ctrl
kDa
48
35

## Slide 3
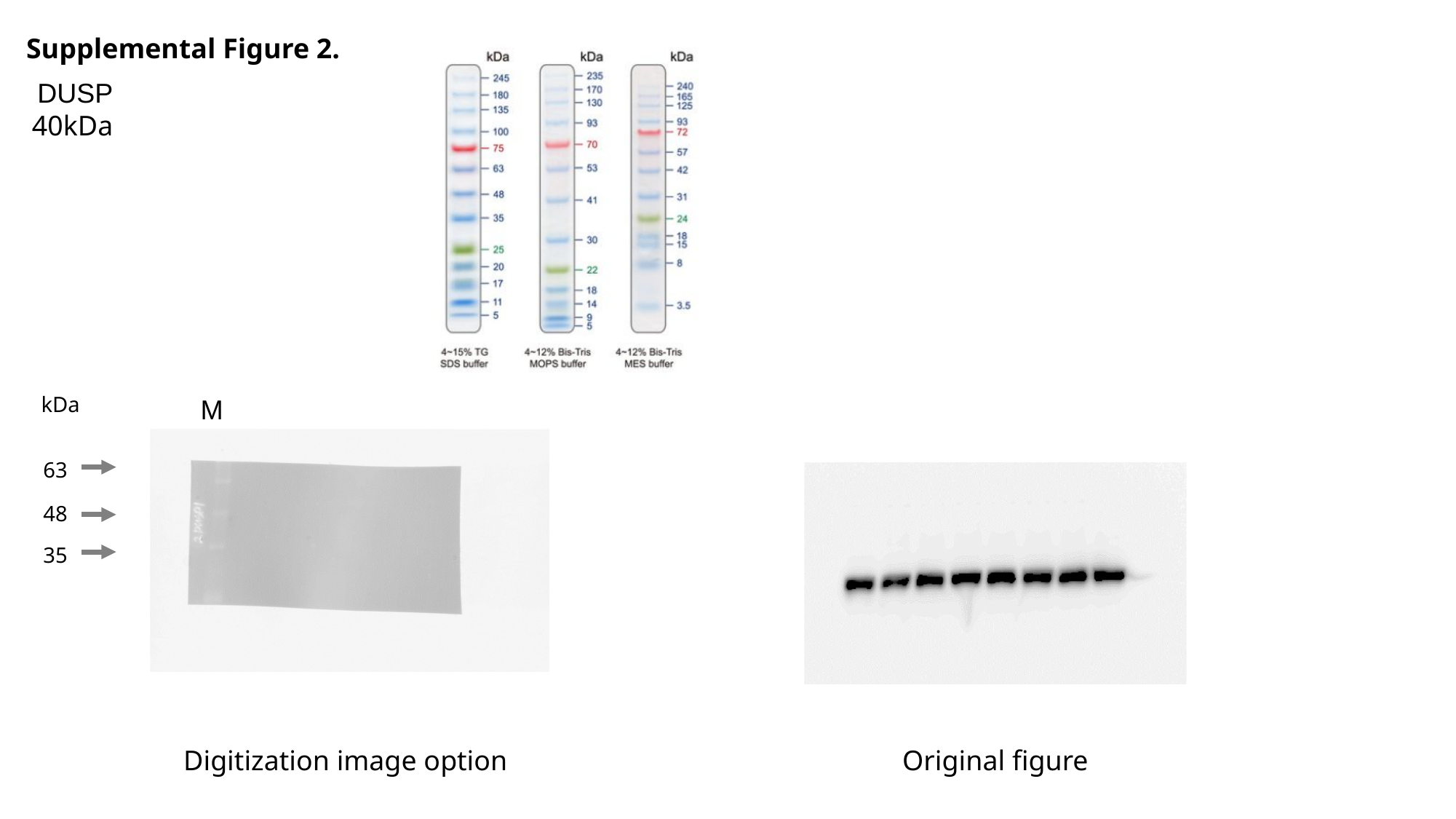

Supplemental Figure 2.
DUSP
40kDa
kDa
M
63
48
35
Digitization image option
Original figure

## Slide 4
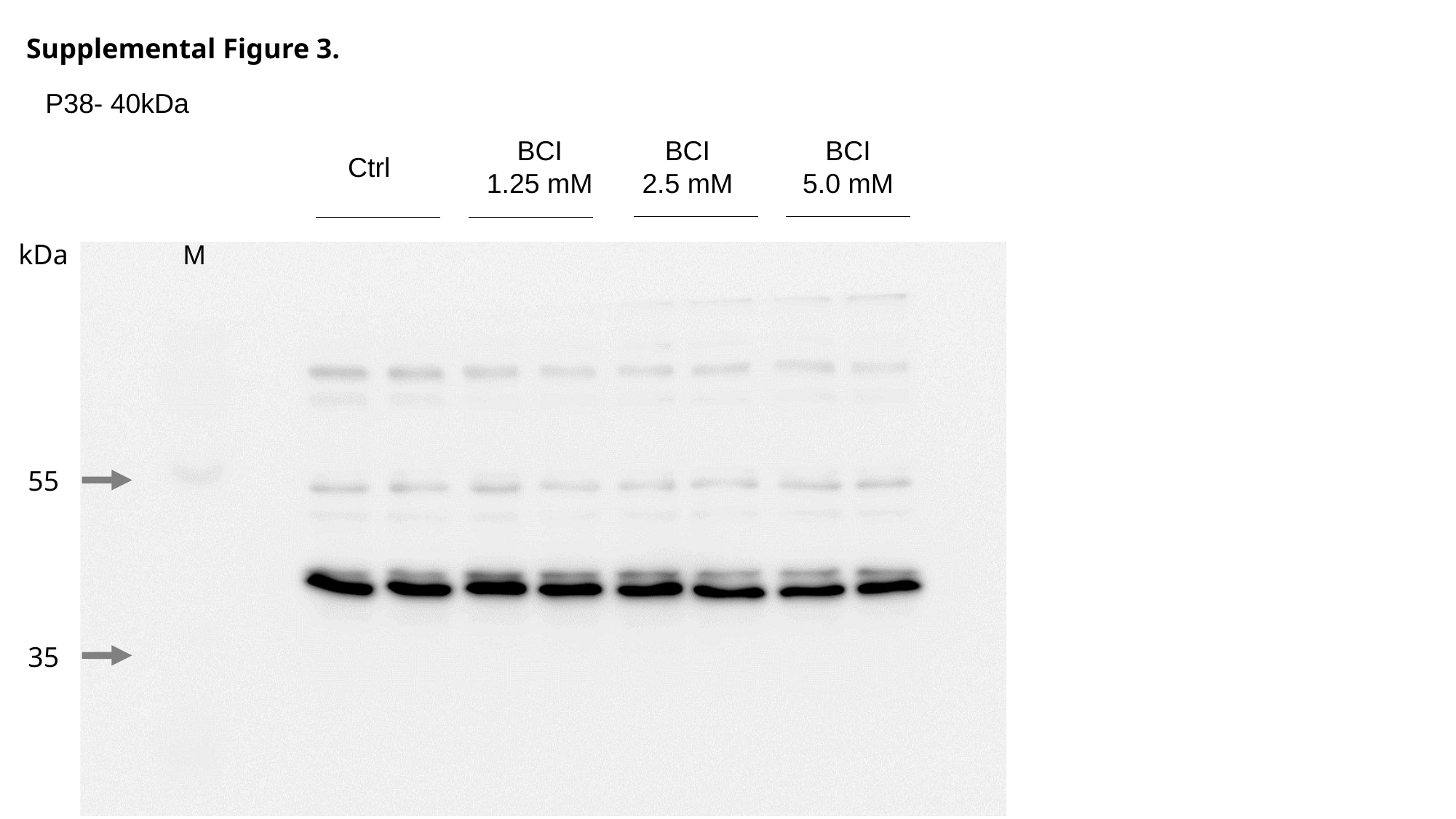

Supplemental Figure 3.
P38- 40kDa
BCI
5.0 mM
BCI
1.25 mM
BCI
2.5 mM
Ctrl
kDa
M
55
35

## Slide 5
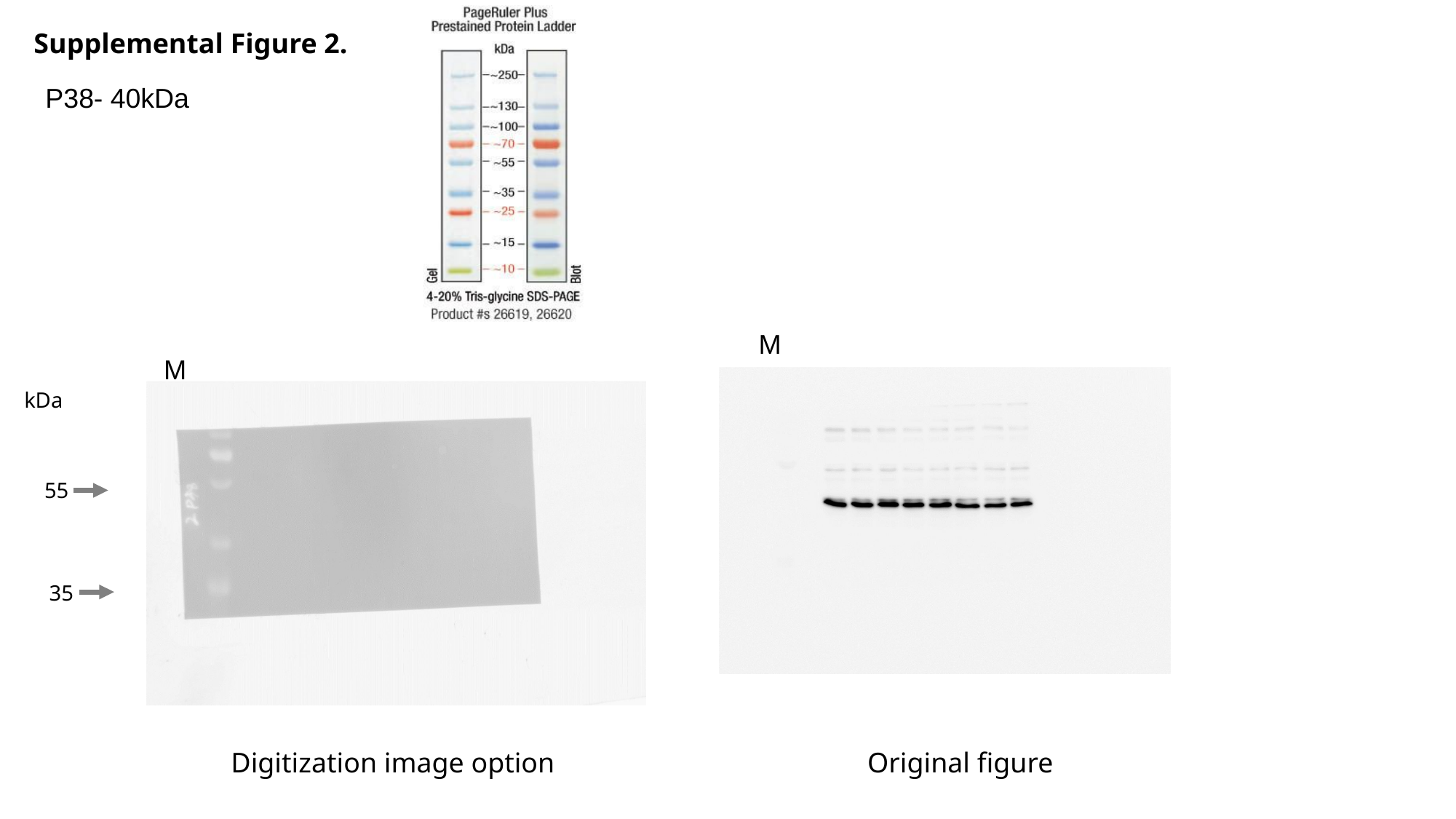

Supplemental Figure 2.
P38- 40kDa
M
M
kDa
55
35
Digitization image option
Original figure

## Slide 6
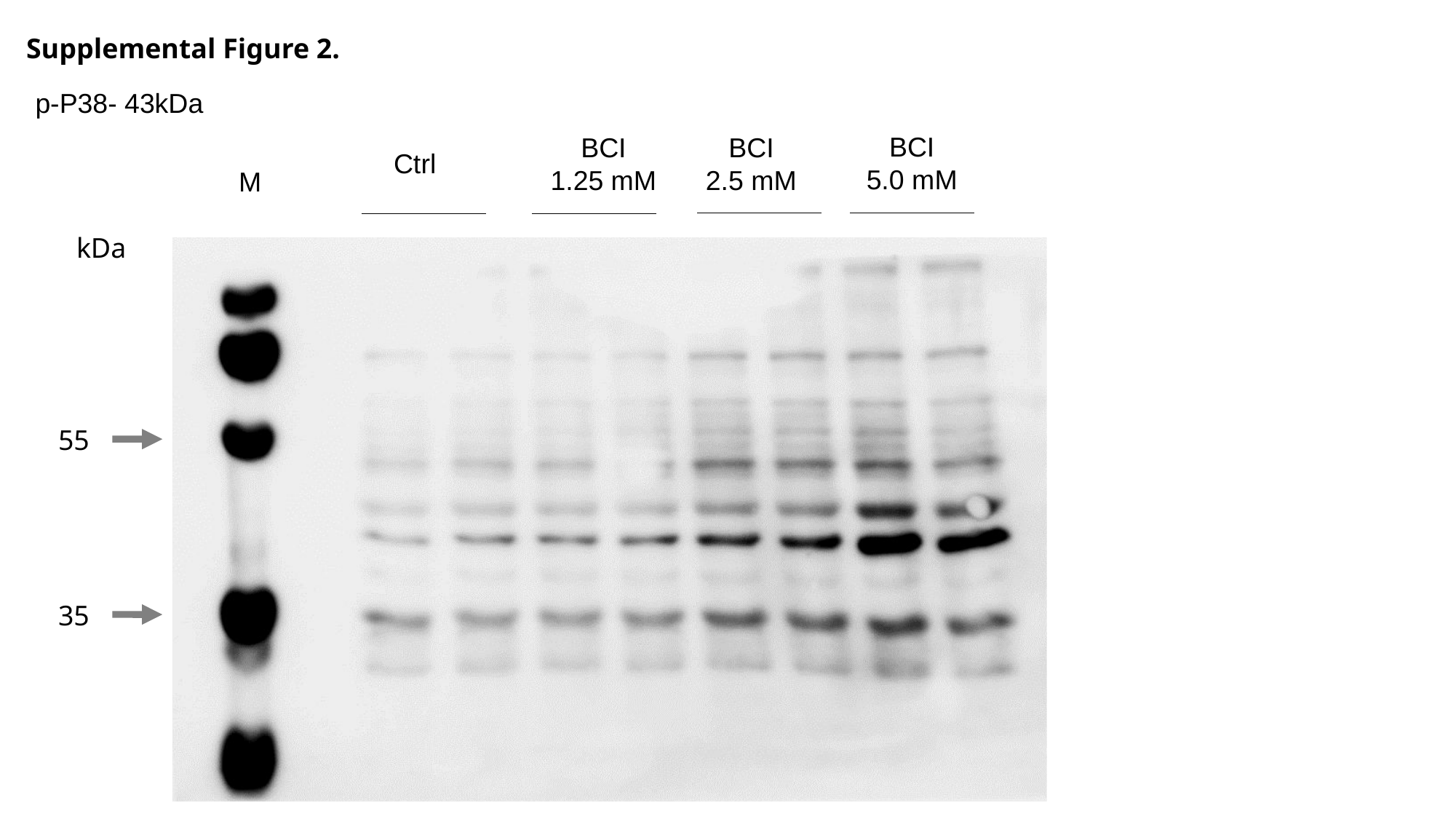

Supplemental Figure 2.
p-P38- 43kDa
BCI
5.0 mM
BCI
1.25 mM
BCI
2.5 mM
Ctrl
M
kDa
55
35

## Slide 7
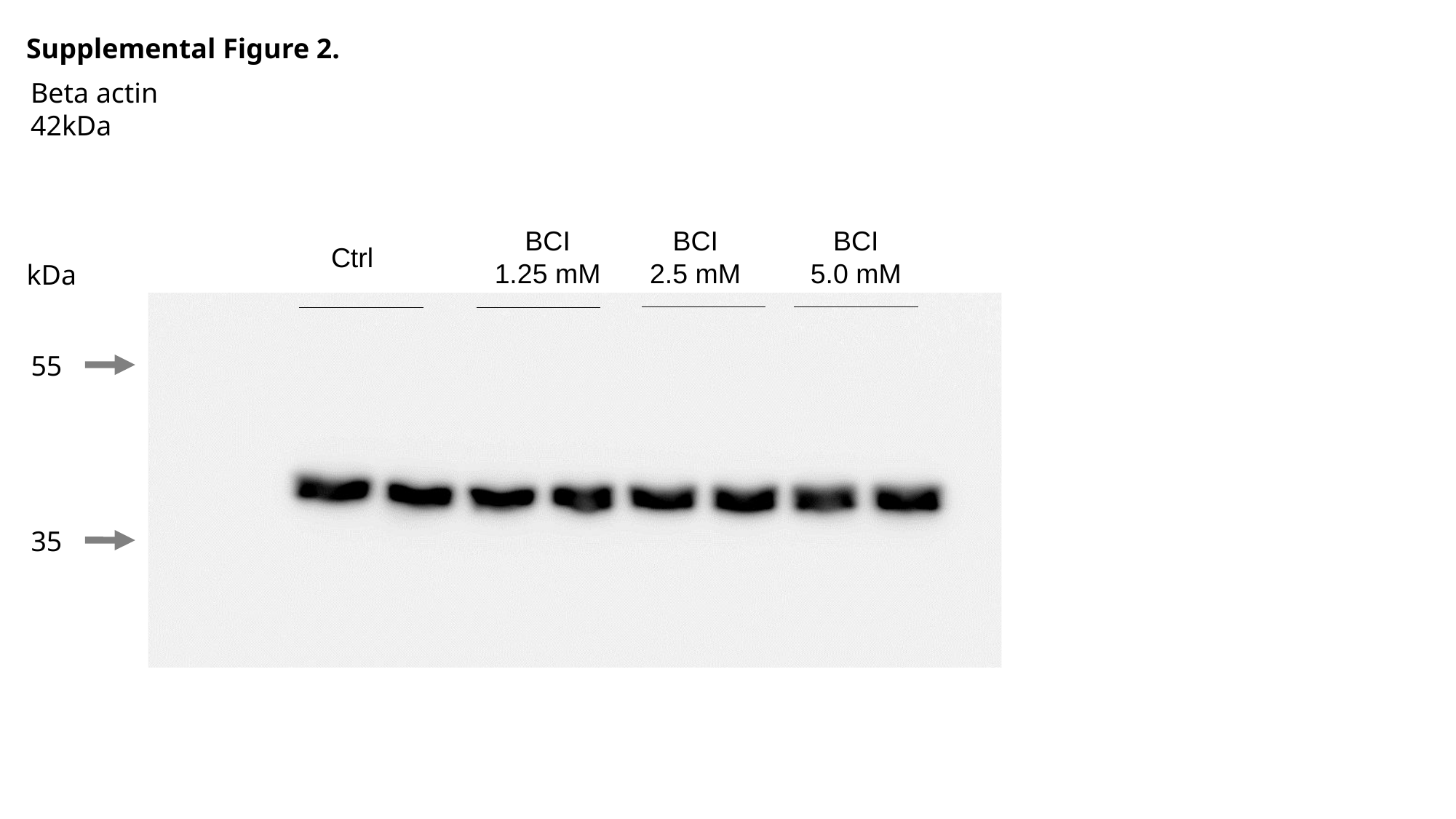

Supplemental Figure 2.
Beta actin
42kDa
BCI
5.0 mM
BCI
1.25 mM
BCI
2.5 mM
Ctrl
kDa
55
35

## Slide 8
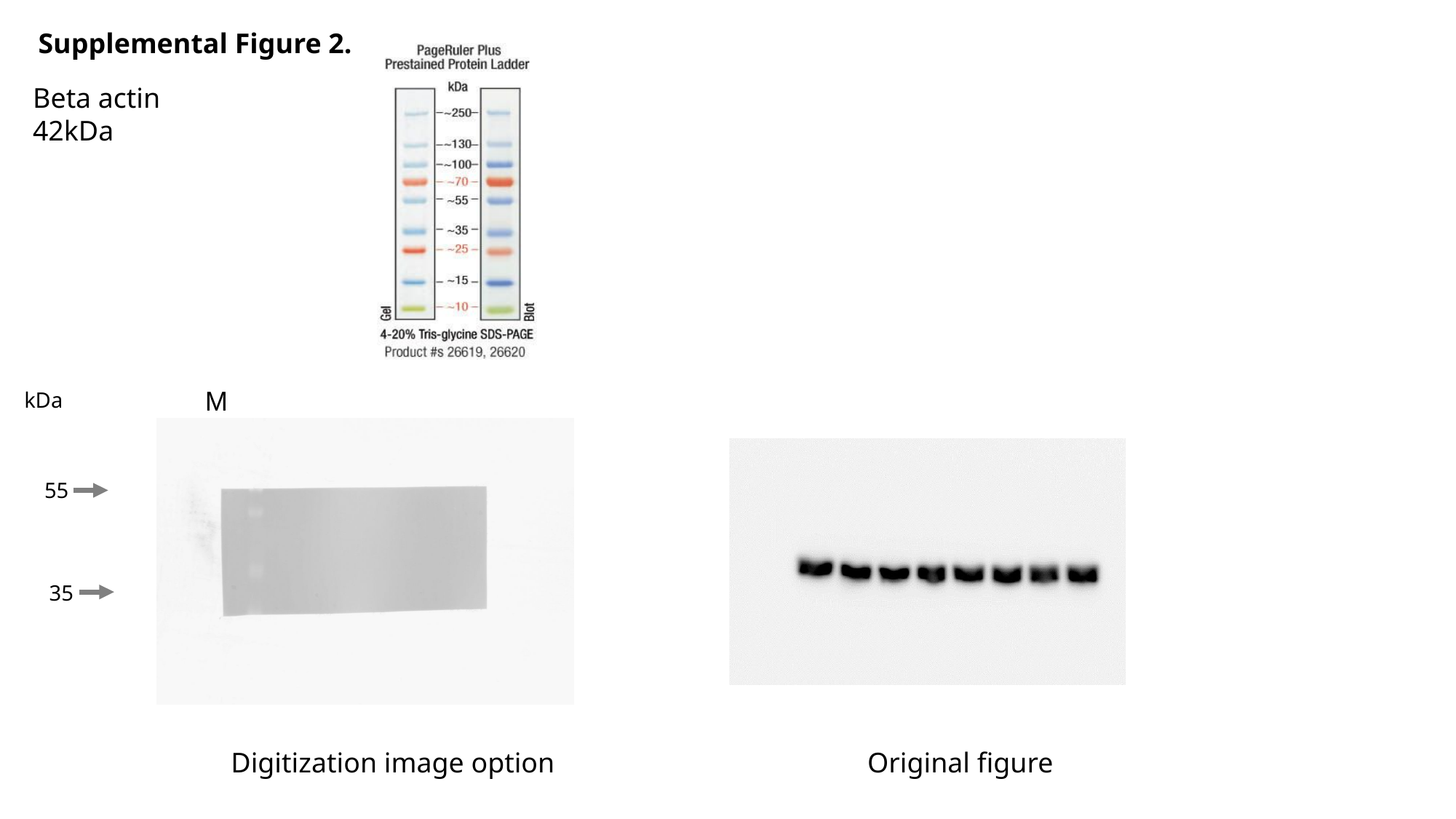

Supplemental Figure 2.
Beta actin
42kDa
M
kDa
55
35
Digitization image option
Original figure

## Slide 9
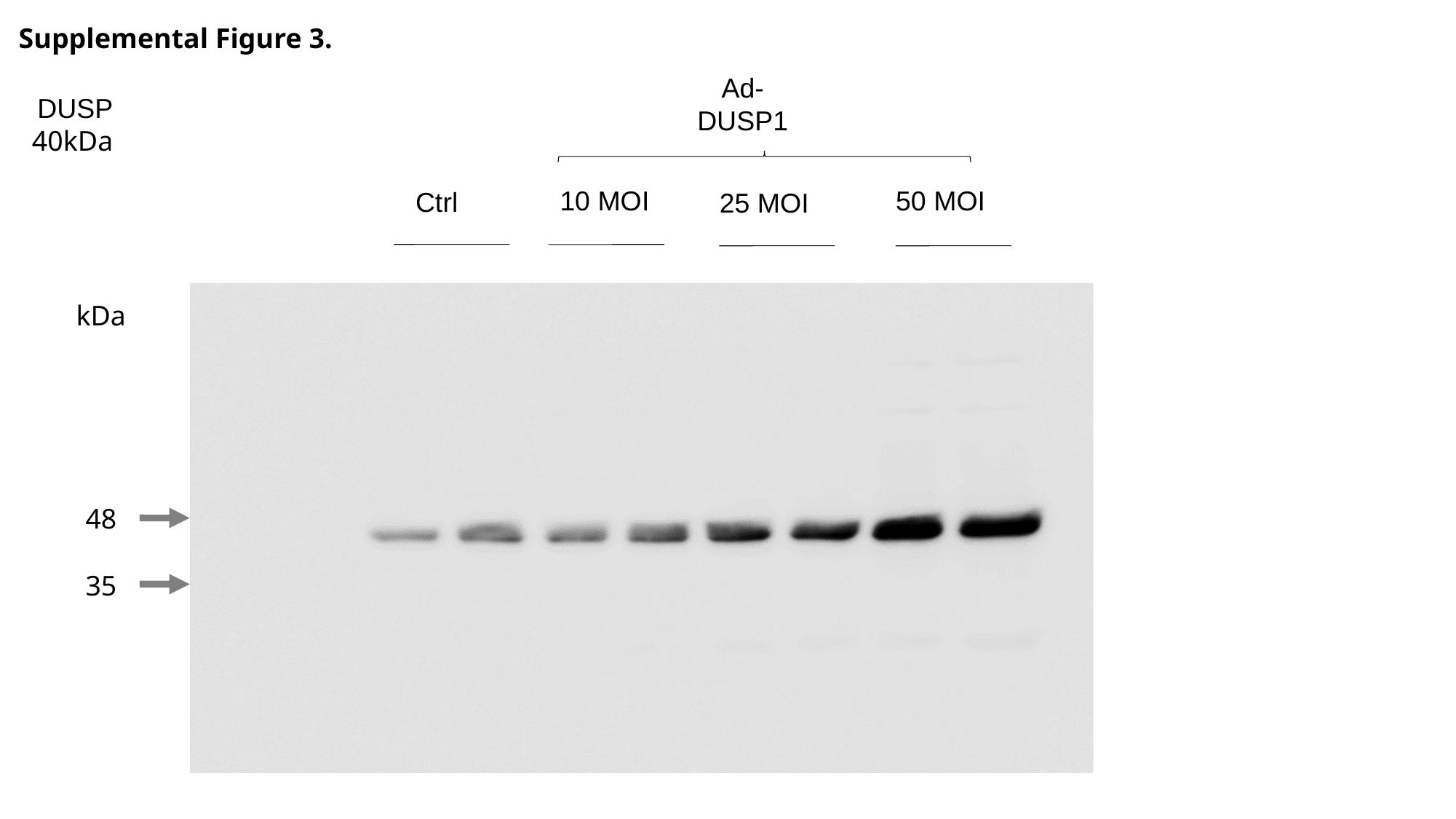

Supplemental Figure 3.
Ad-DUSP1
DUSP
40kDa
10 MOI
50 MOI
Ctrl
25 MOI
kDa
48
35

## Slide 10
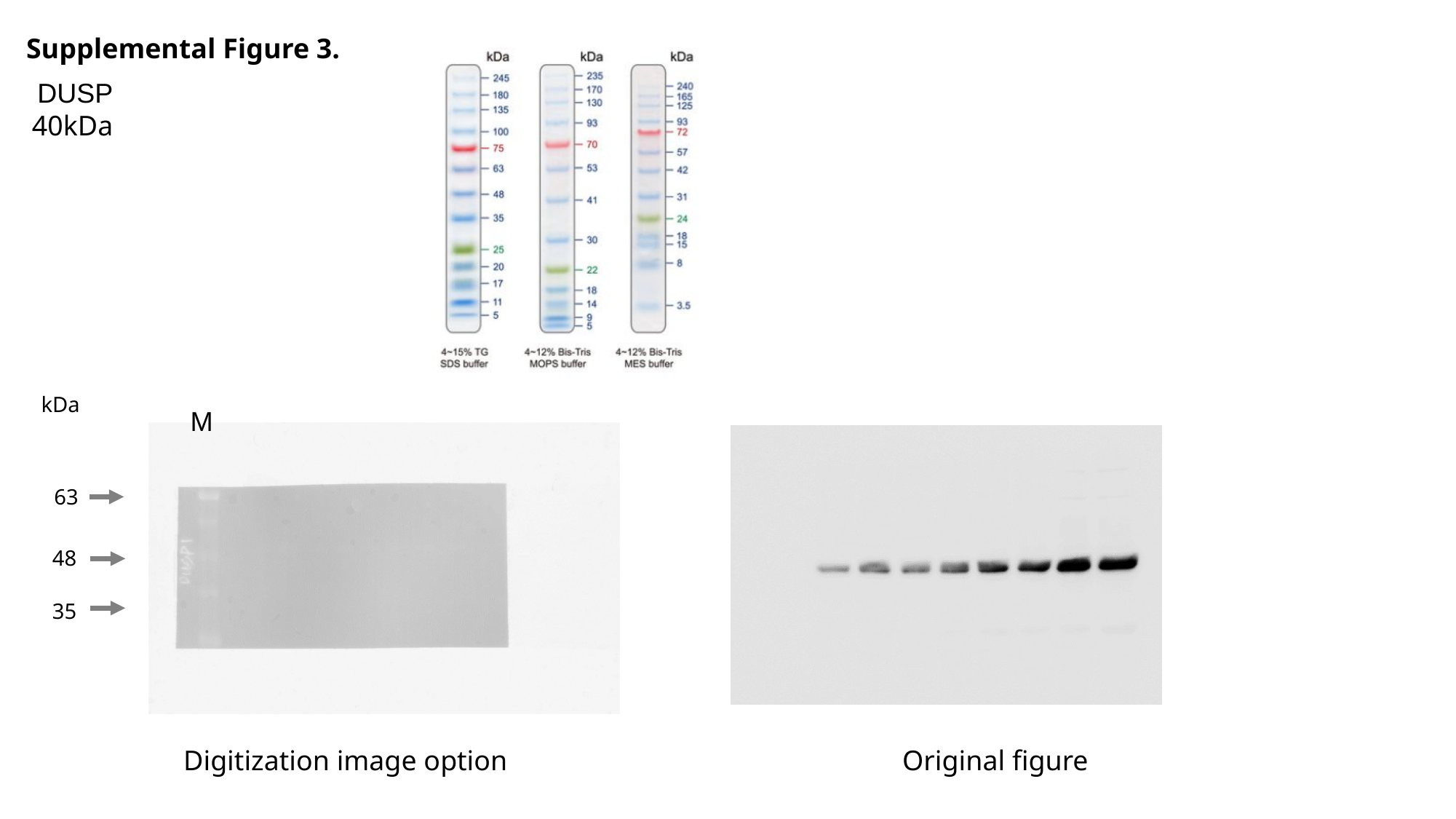

Supplemental Figure 3.
DUSP
40kDa
kDa
M
63
48
35
Digitization image option
Original figure

## Slide 11
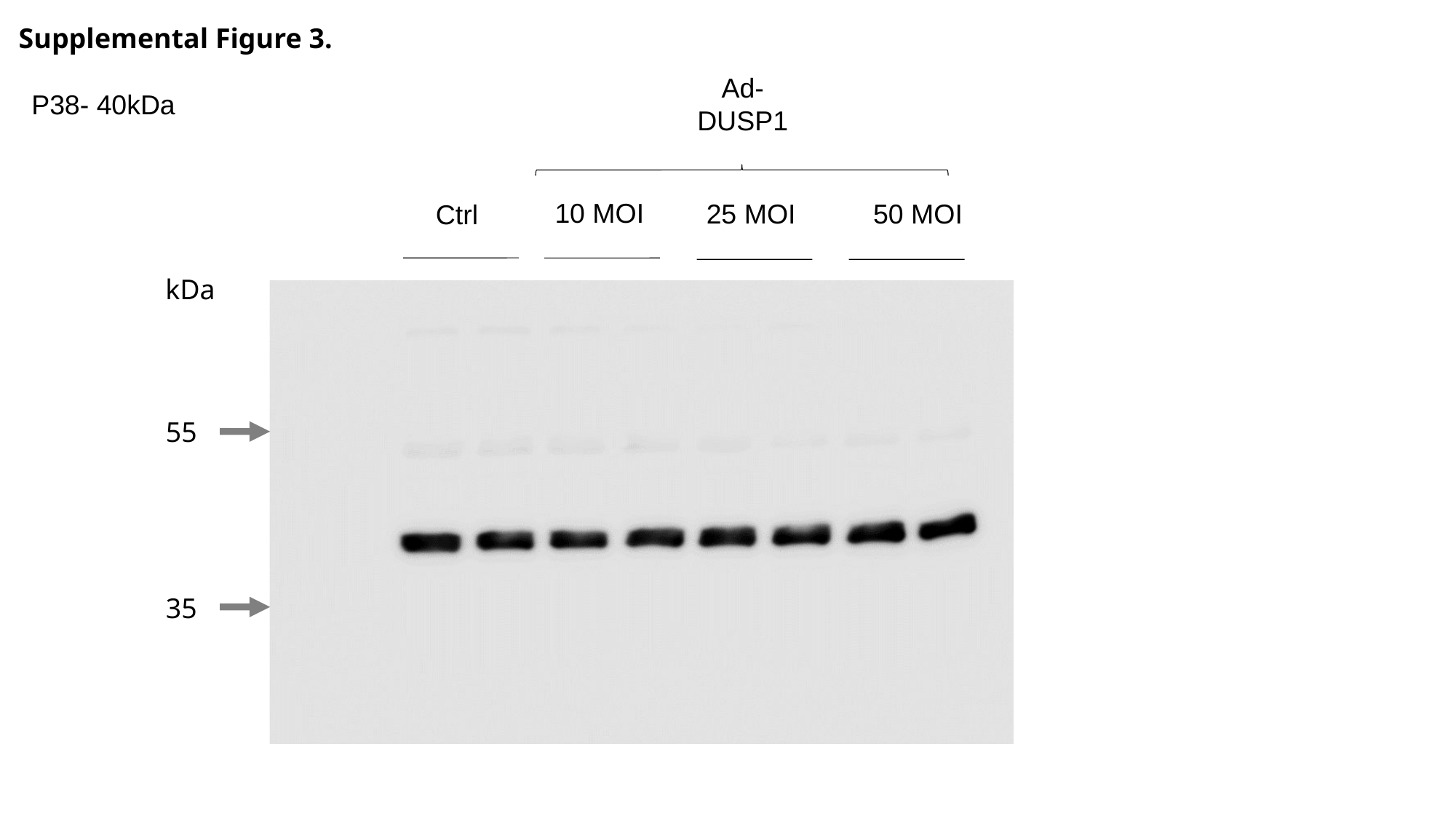

Supplemental Figure 3.
Ad-DUSP1
P38- 40kDa
10 MOI
25 MOI
50 MOI
Ctrl
kDa
55
35

## Slide 12
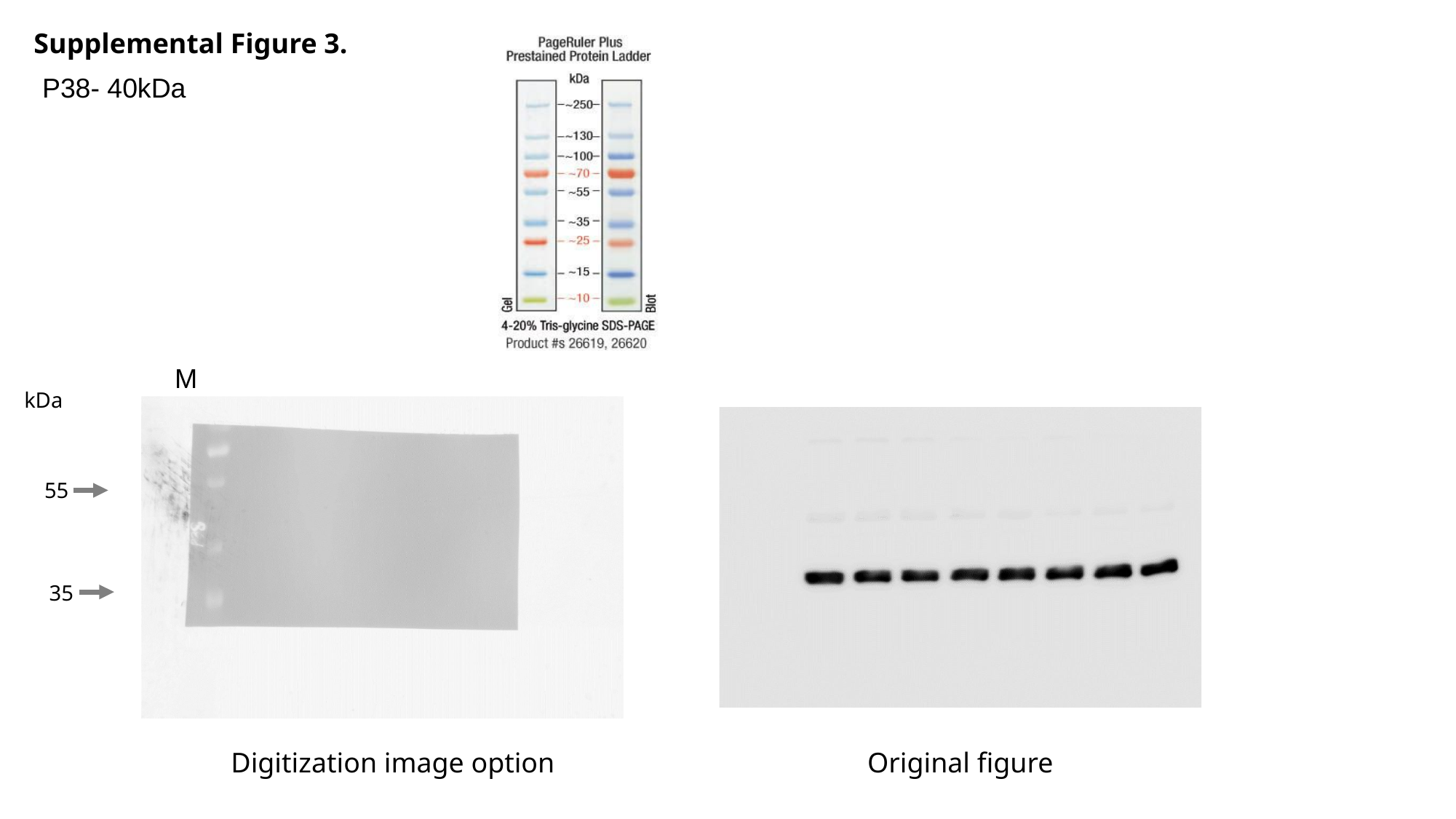

Supplemental Figure 3.
P38- 40kDa
M
kDa
55
35
Digitization image option
Original figure

## Slide 13
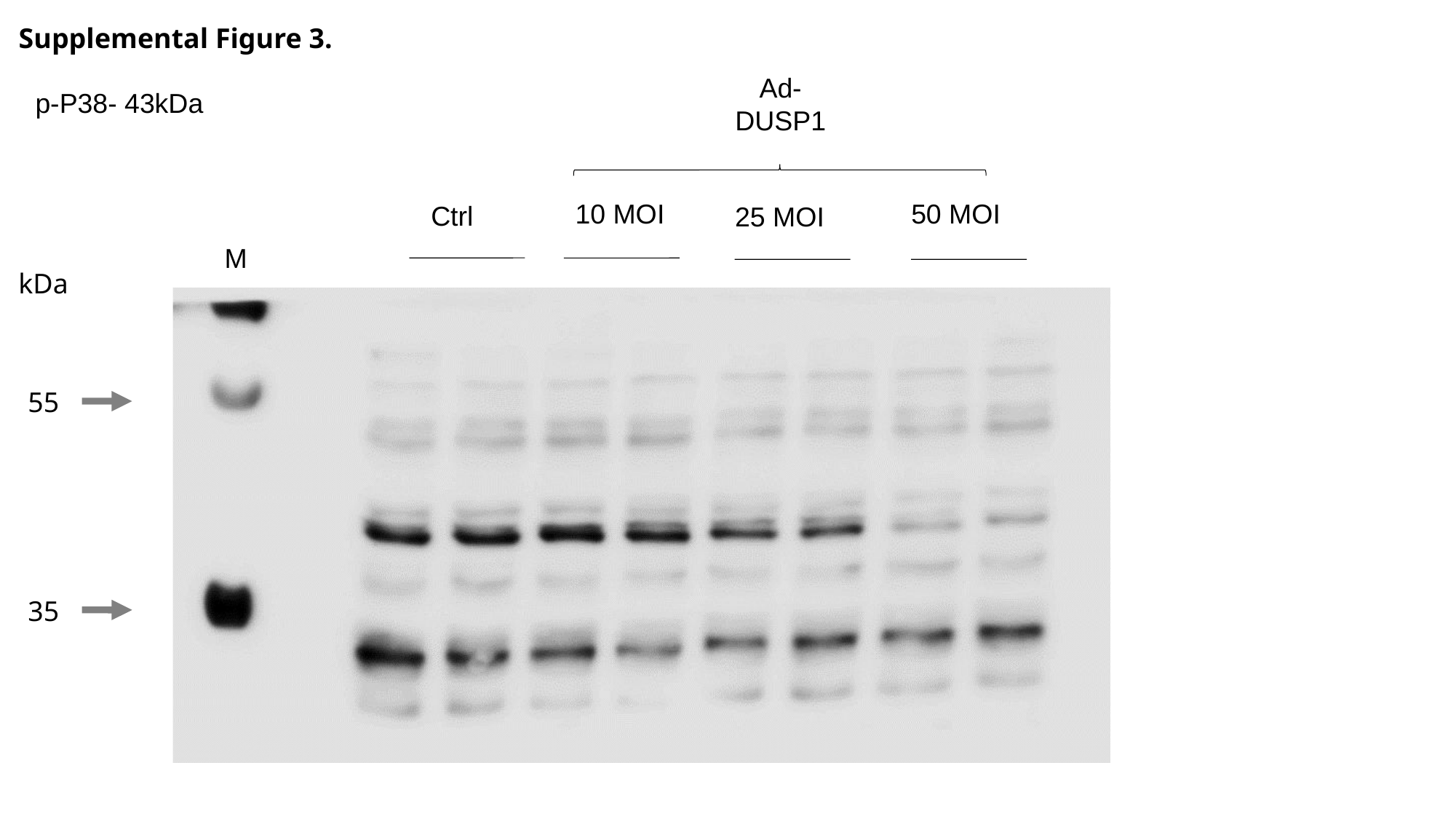

Supplemental Figure 3.
Ad-DUSP1
p-P38- 43kDa
10 MOI
50 MOI
Ctrl
25 MOI
M
kDa
55
35

## Slide 14
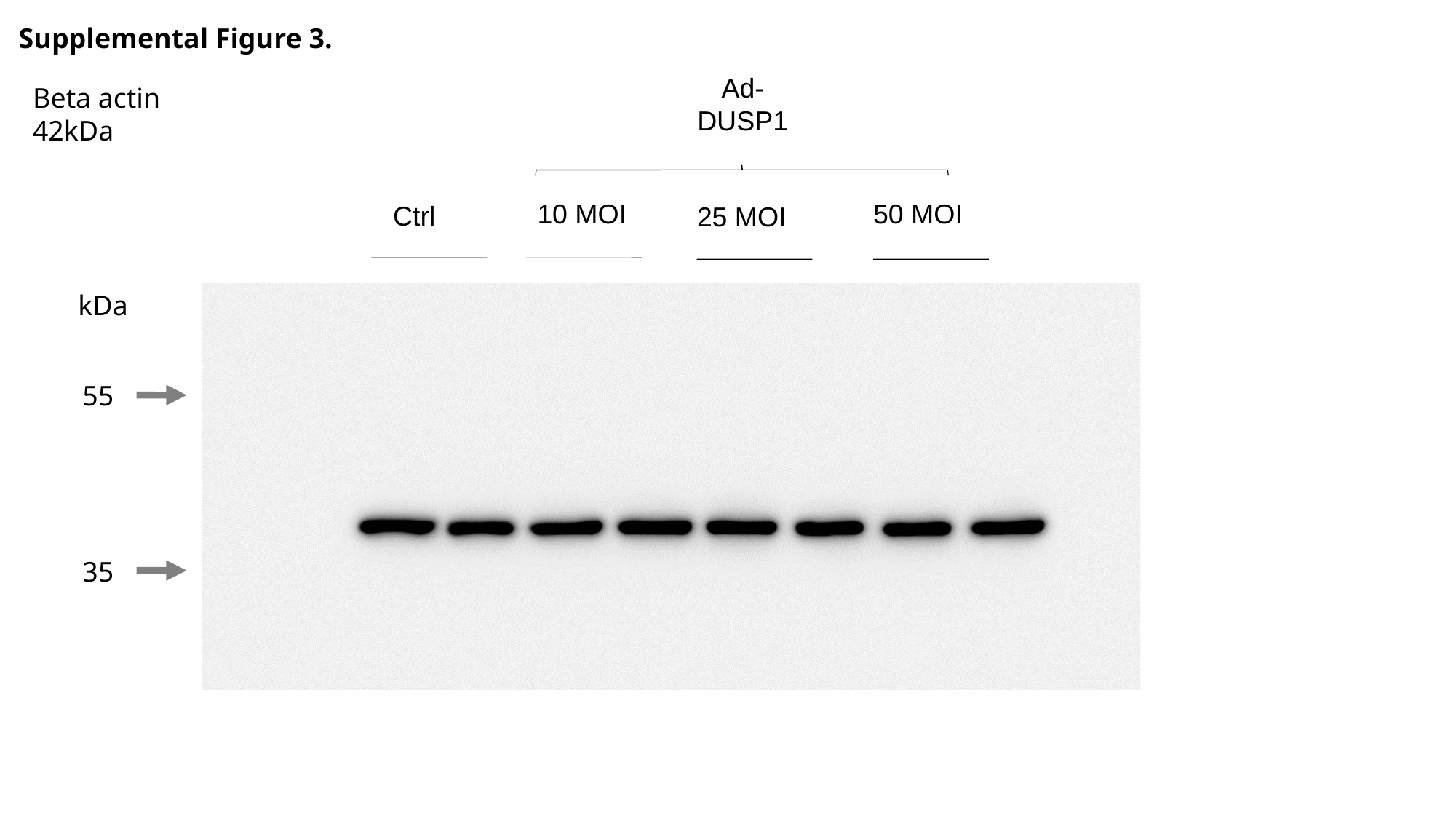

Supplemental Figure 3.
Ad-DUSP1
Beta actin
42kDa
10 MOI
50 MOI
Ctrl
25 MOI
kDa
55
35

## Slide 15
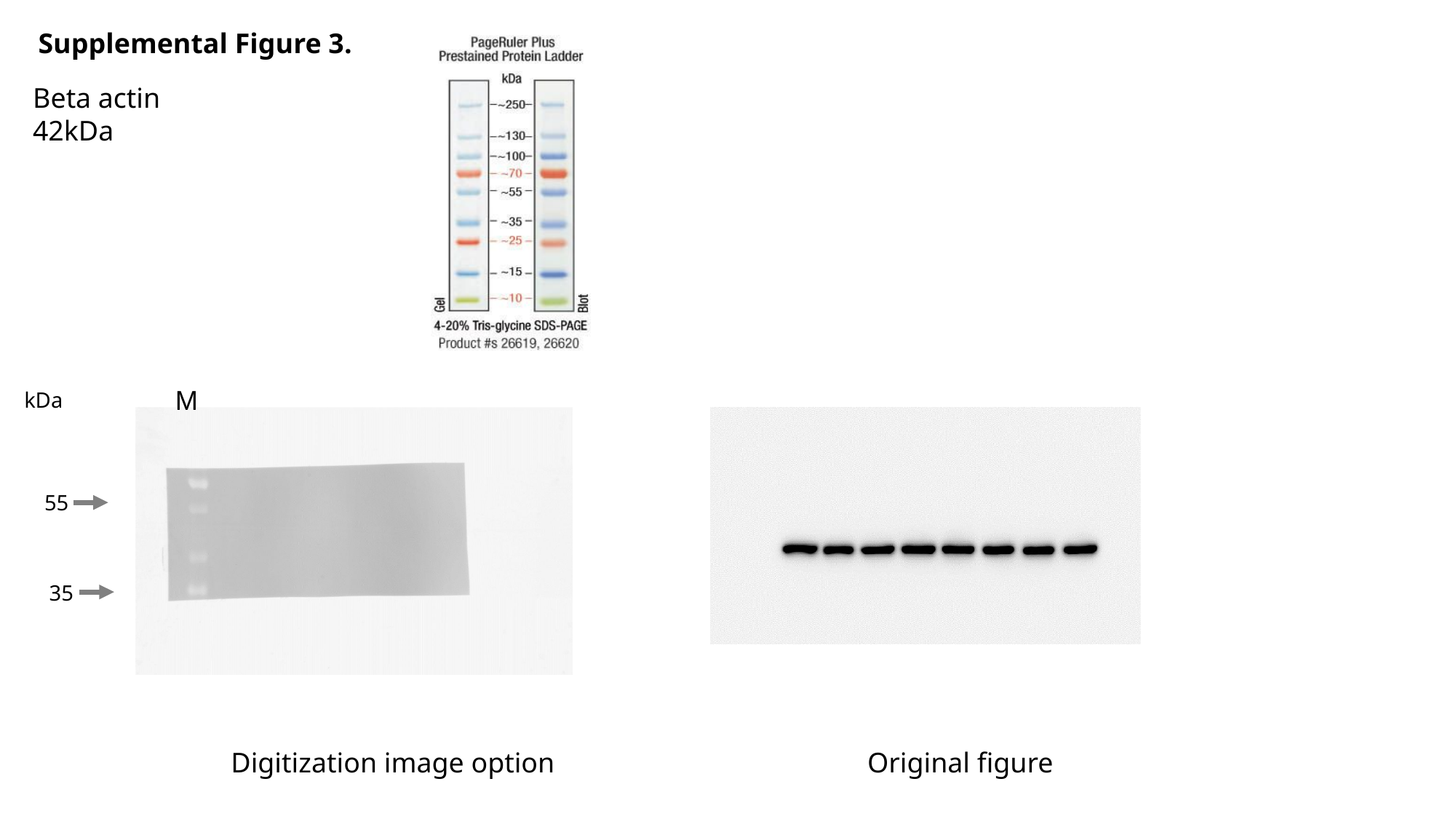

Supplemental Figure 3.
Beta actin
42kDa
M
kDa
55
35
Digitization image option
Original figure

## Slide 16
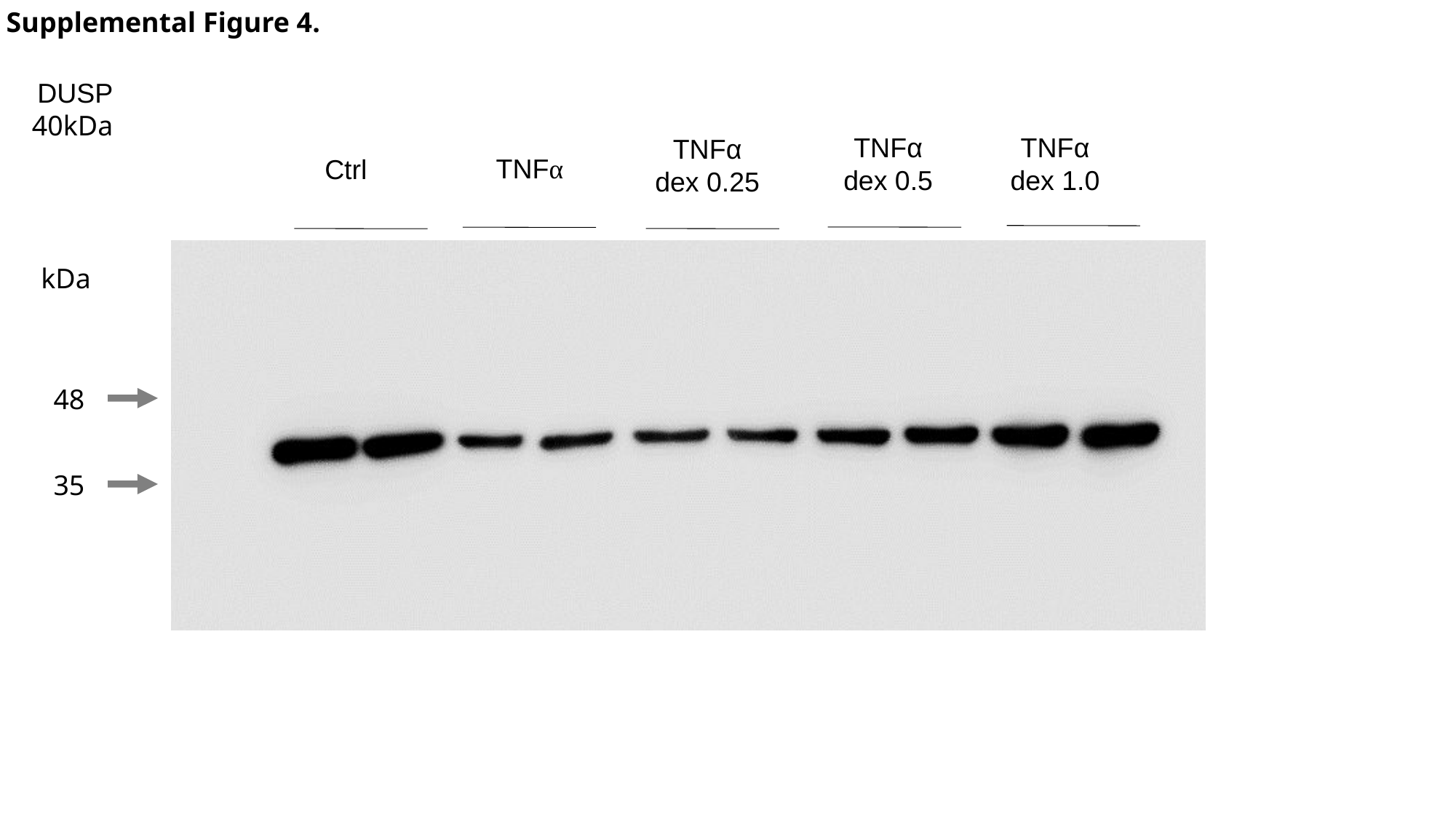

Supplemental Figure 4.
DUSP
40kDa
TNFα
dex 0.5
TNFα
dex 1.0
TNFα
dex 0.25
TNFα
Ctrl
kDa
48
35

## Slide 17
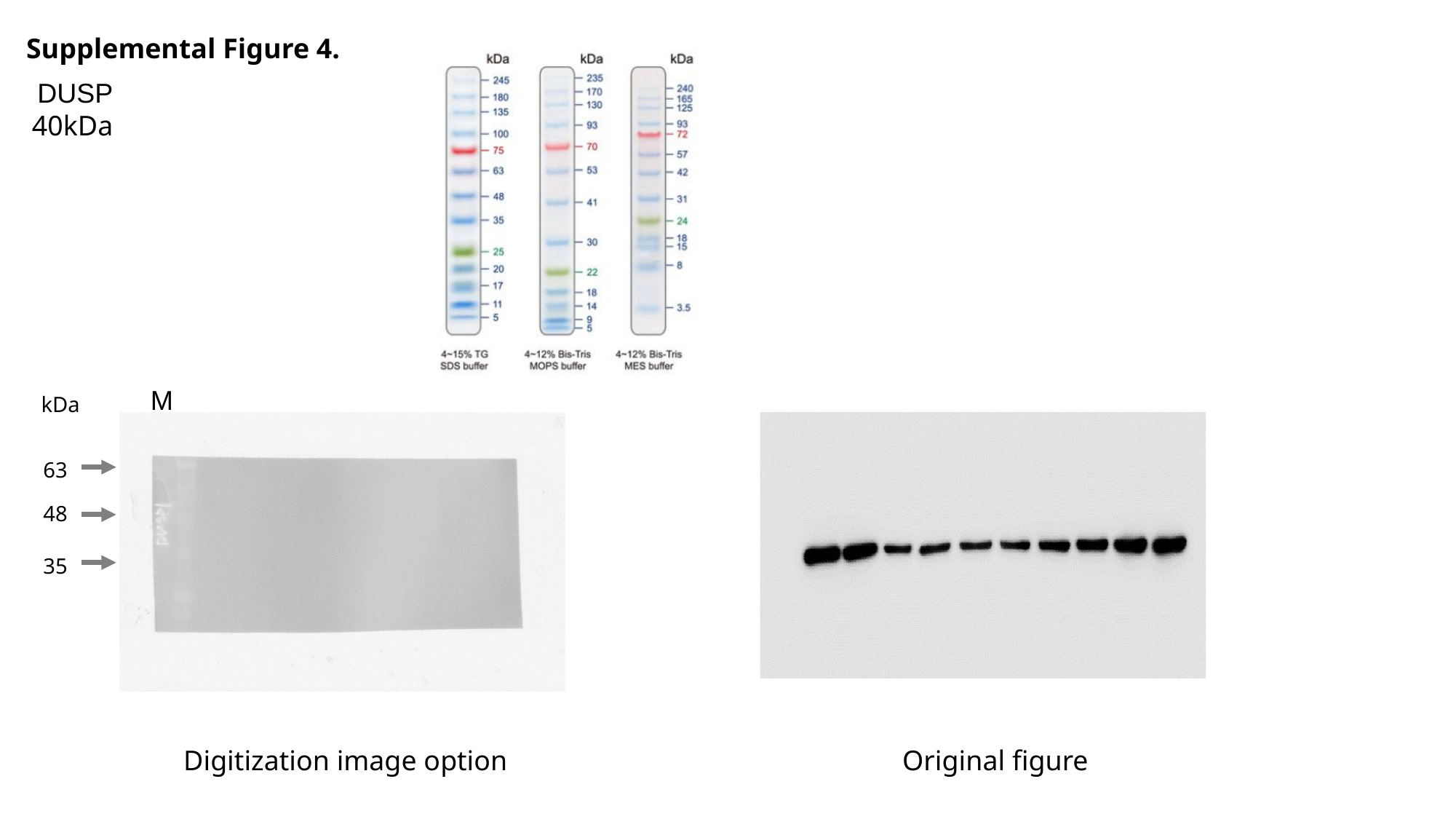

Supplemental Figure 4.
DUSP
40kDa
M
kDa
63
48
35
Digitization image option
Original figure

## Slide 18
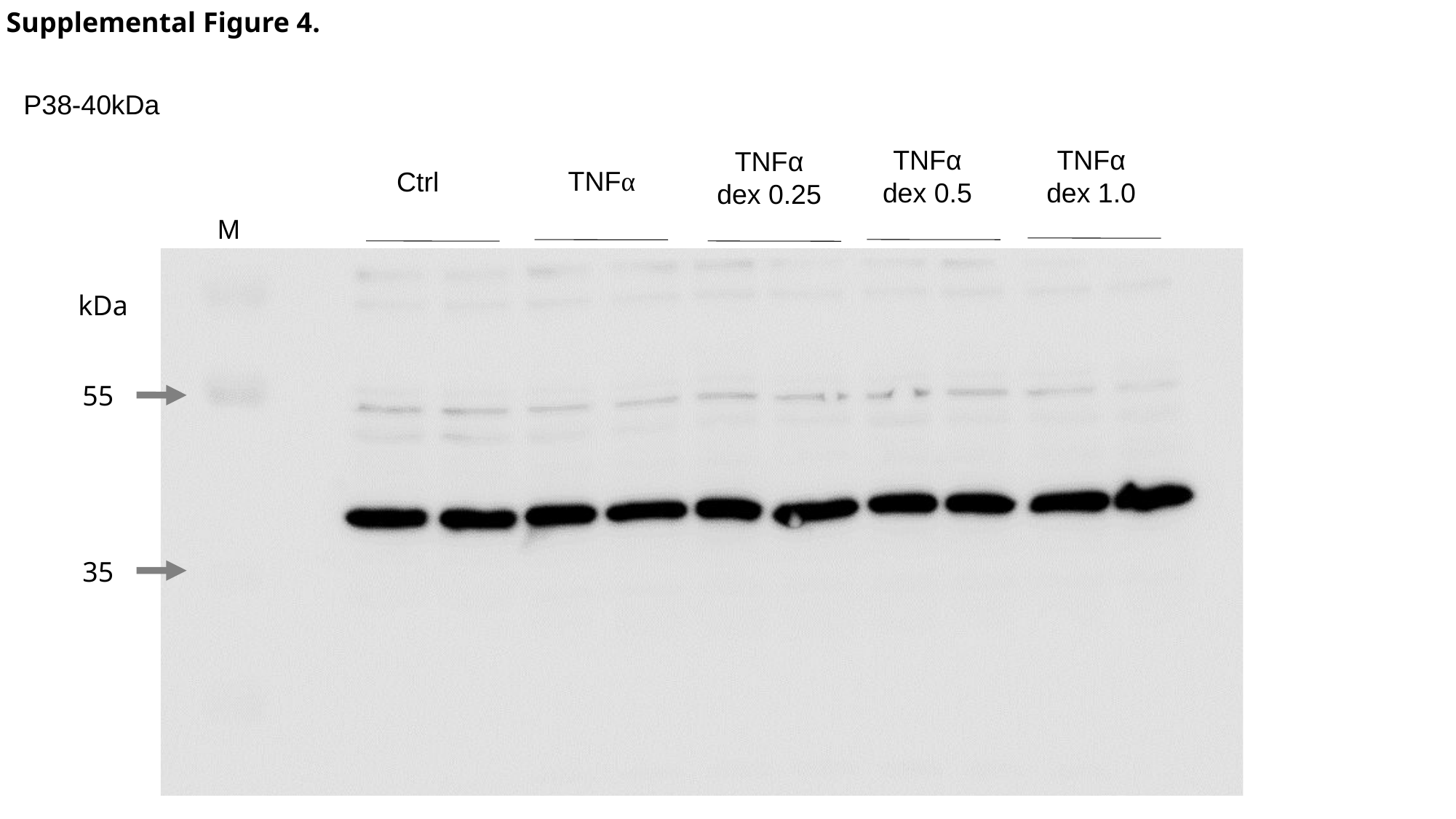

Supplemental Figure 4.
P38-40kDa
TNFα
dex 0.5
TNFα
dex 1.0
TNFα
dex 0.25
TNFα
Ctrl
M
kDa
55
35

## Slide 19
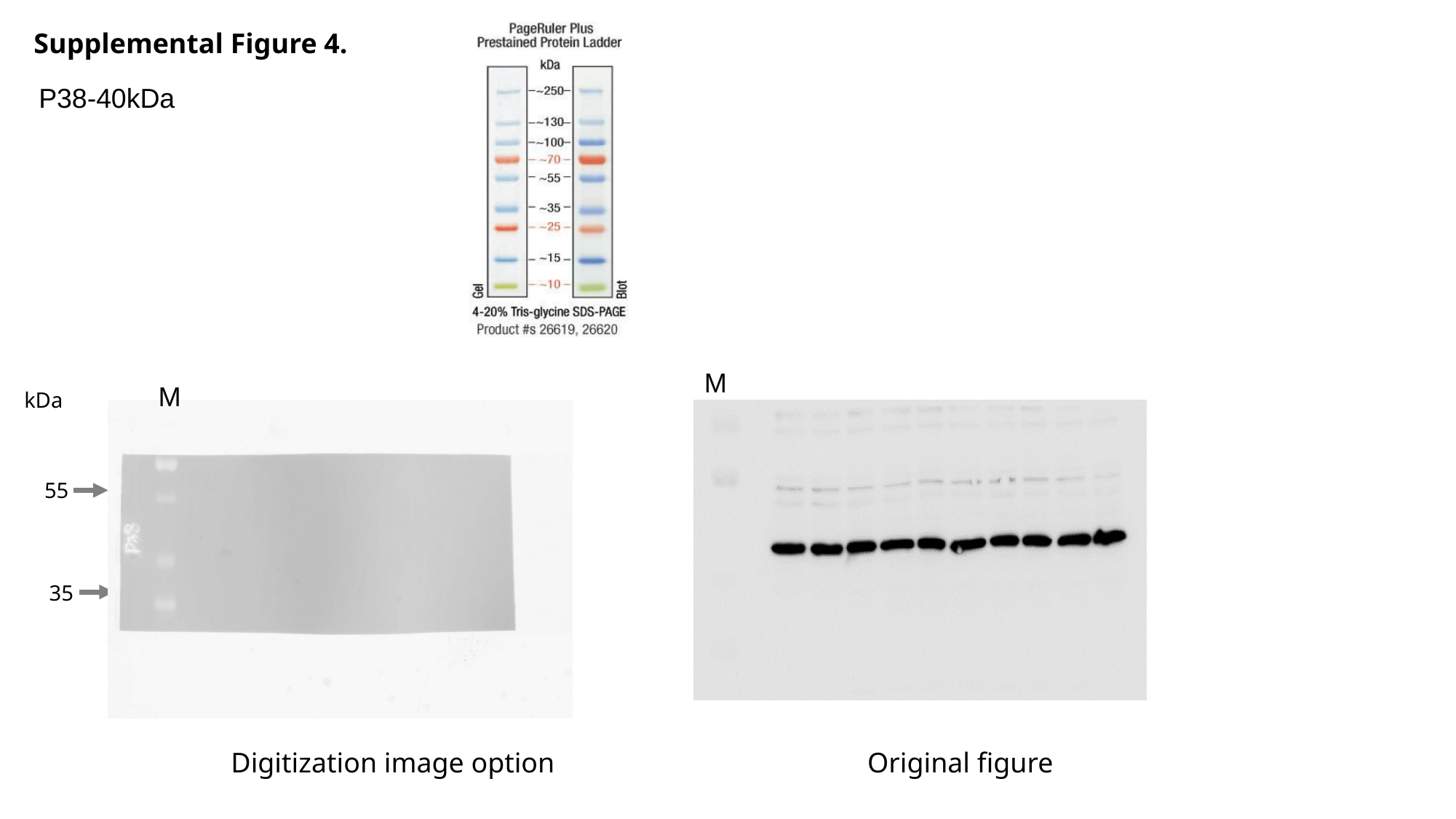

Supplemental Figure 4.
P38-40kDa
M
M
kDa
55
35
Digitization image option
Original figure

## Slide 20
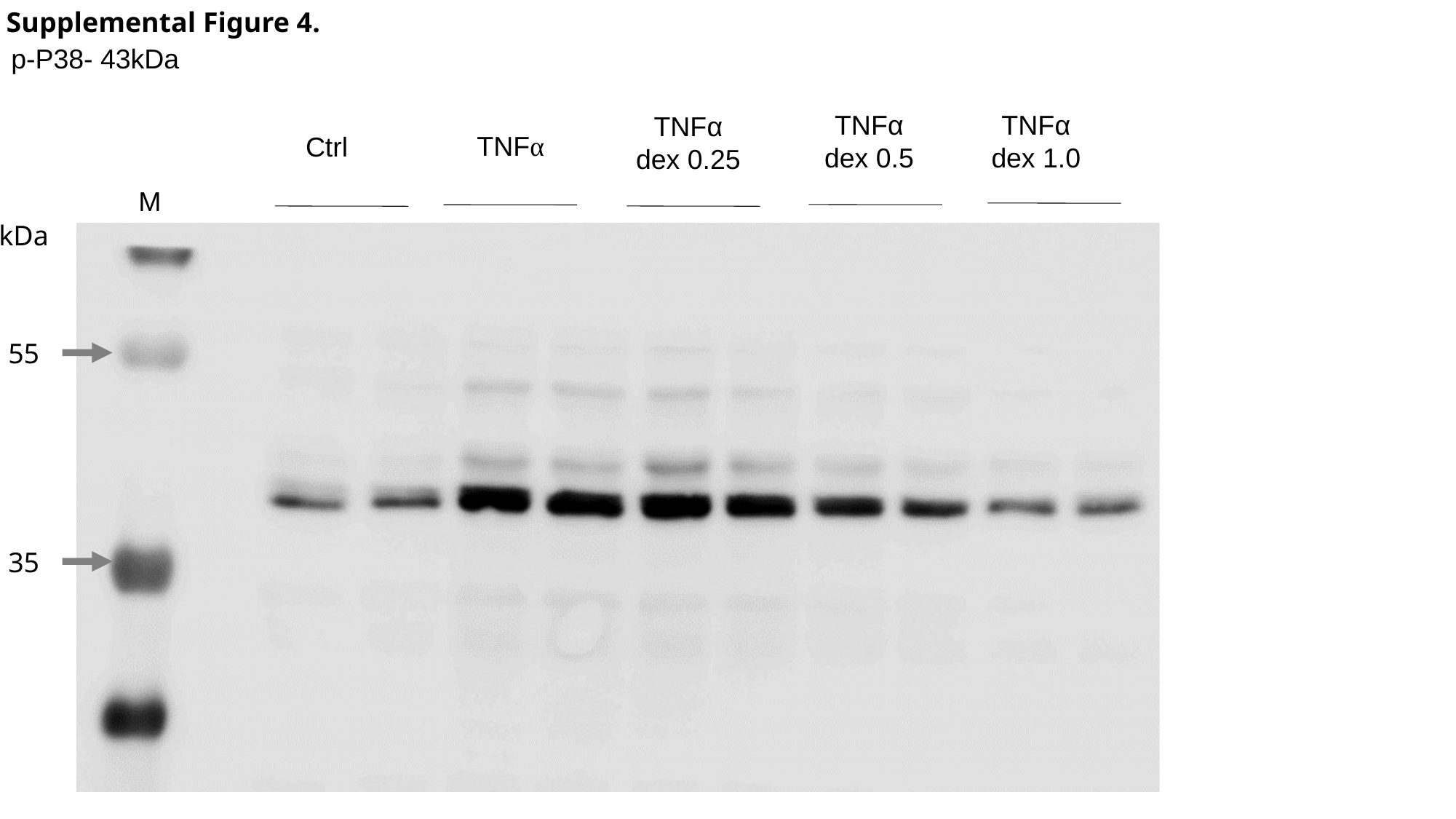

Supplemental Figure 4.
p-P38- 43kDa
TNFα
dex 0.5
TNFα
dex 1.0
TNFα
dex 0.25
TNFα
Ctrl
M
kDa
55
35

## Slide 21
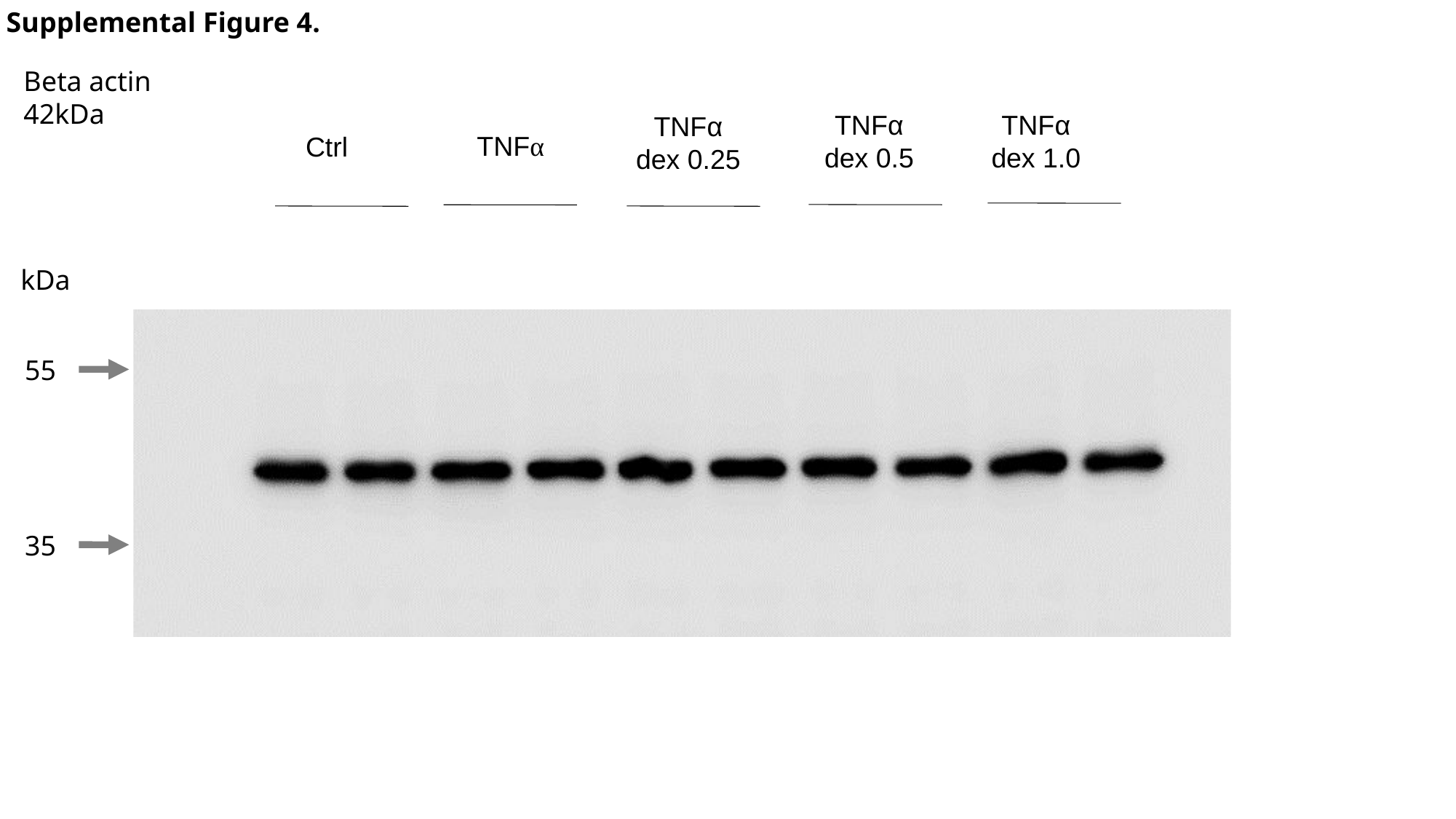

Supplemental Figure 4.
Beta actin
42kDa
TNFα
dex 0.5
TNFα
dex 1.0
TNFα
dex 0.25
TNFα
Ctrl
kDa
55
35

## Slide 22
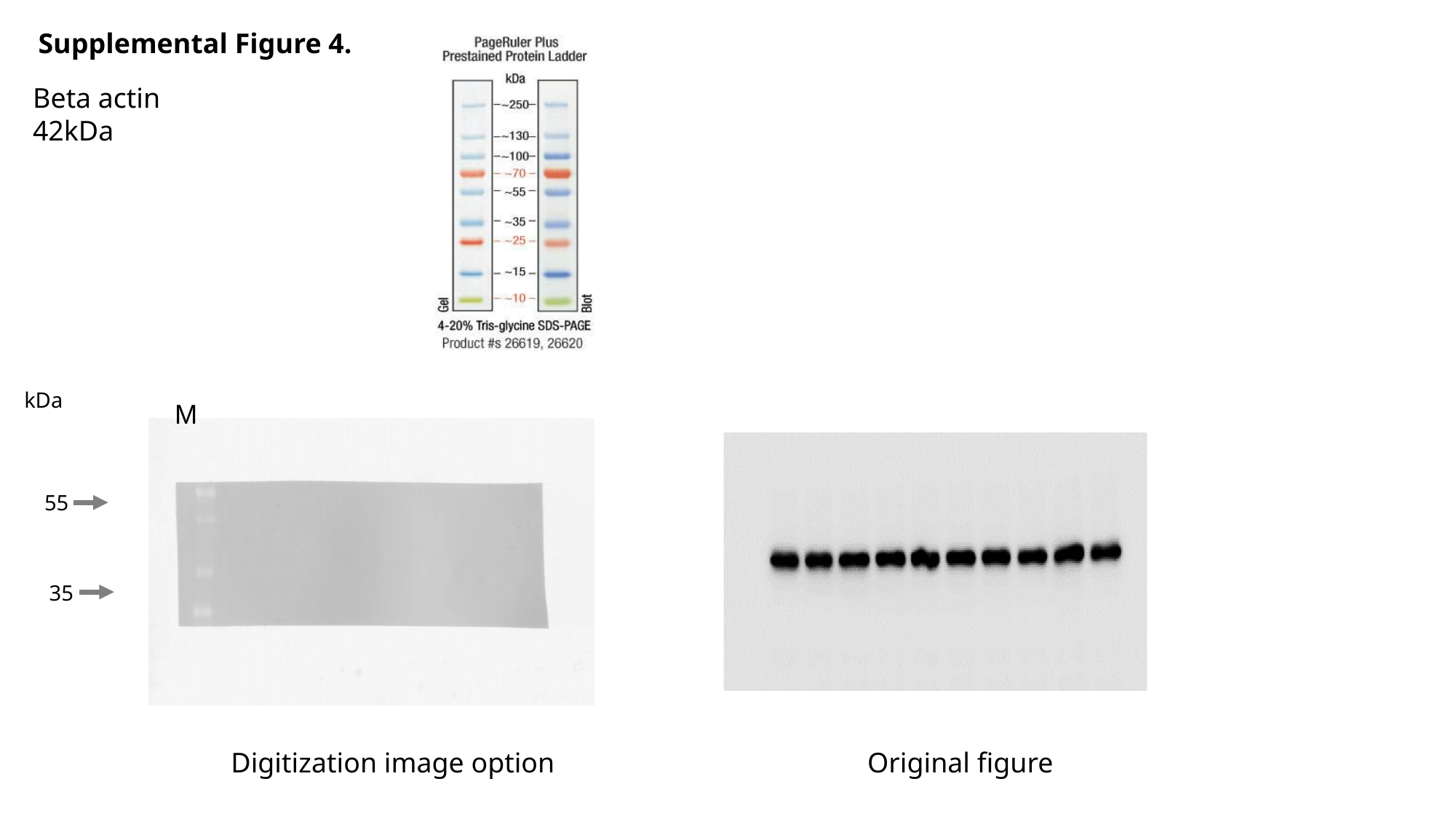

Supplemental Figure 4.
Beta actin
42kDa
kDa
M
55
35
Digitization image option
Original figure

## Slide 23
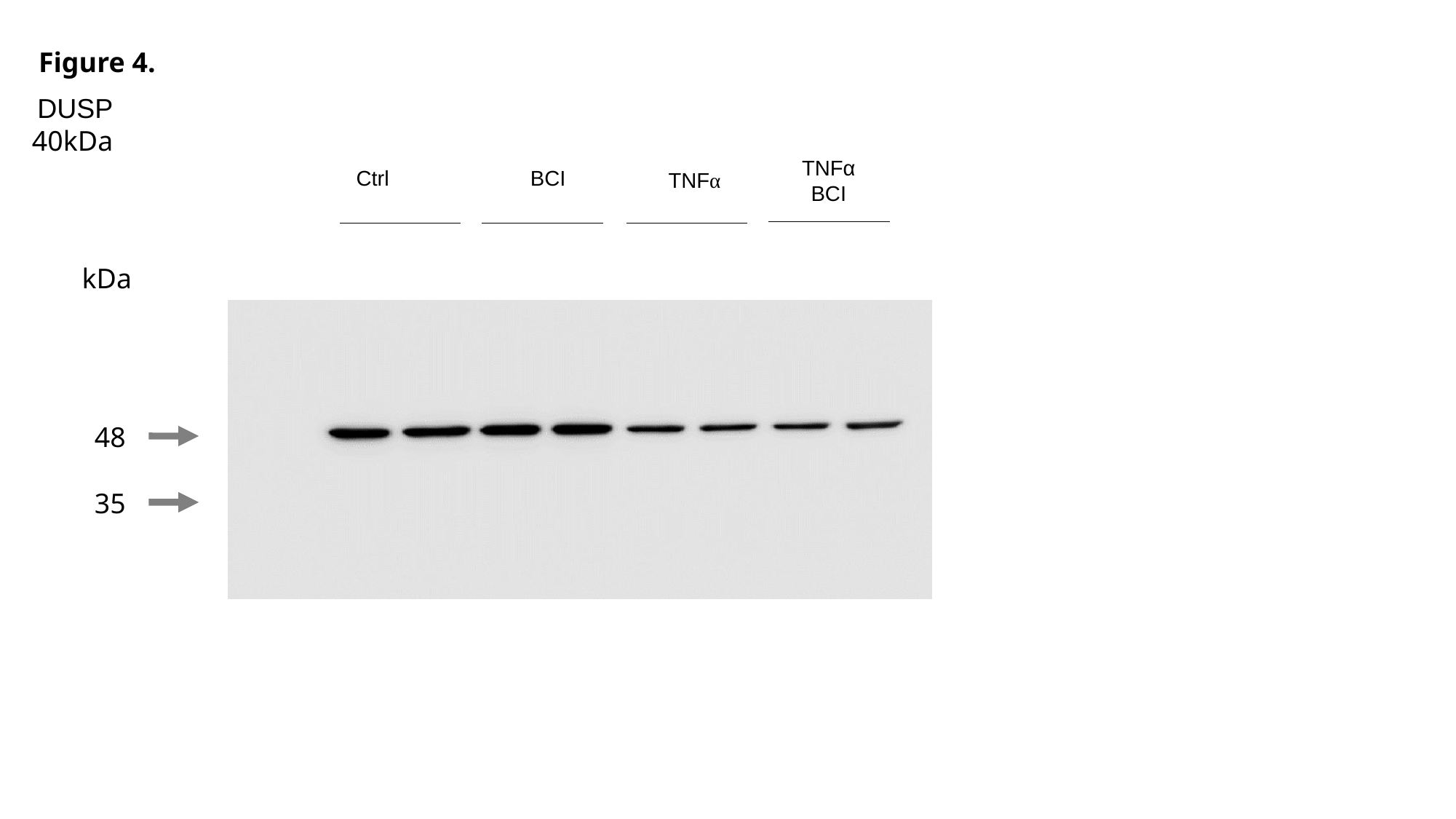

Figure 4.
DUSP
40kDa
TNFα
BCI
Ctrl
BCI
TNFα
kDa
48
35

## Slide 24
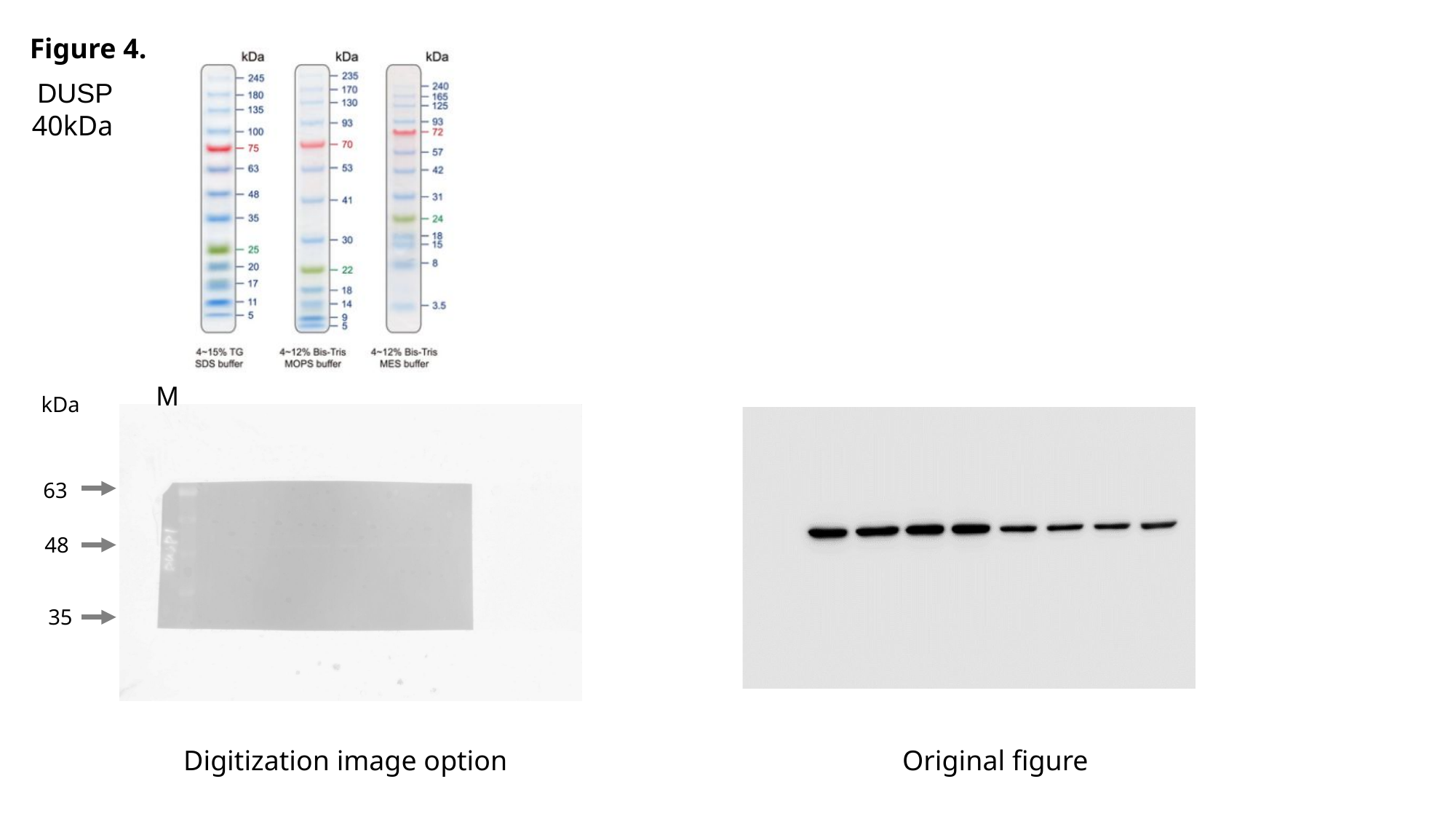

Figure 4.
DUSP
40kDa
M
kDa
63
48
35
Digitization image option
Original figure

## Slide 25
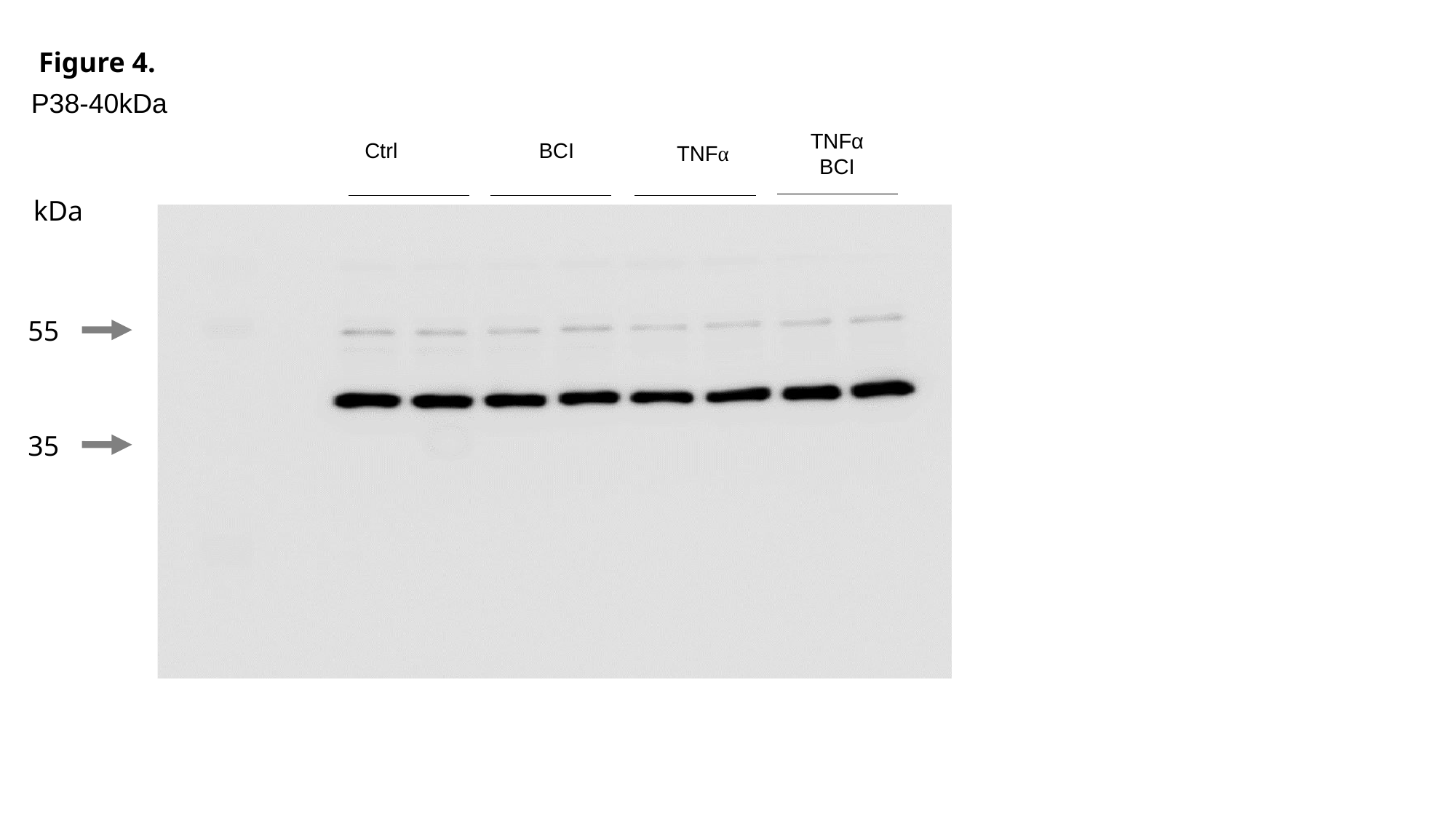

Figure 4.
P38-40kDa
TNFα
BCI
Ctrl
BCI
TNFα
kDa
55
35

## Slide 26
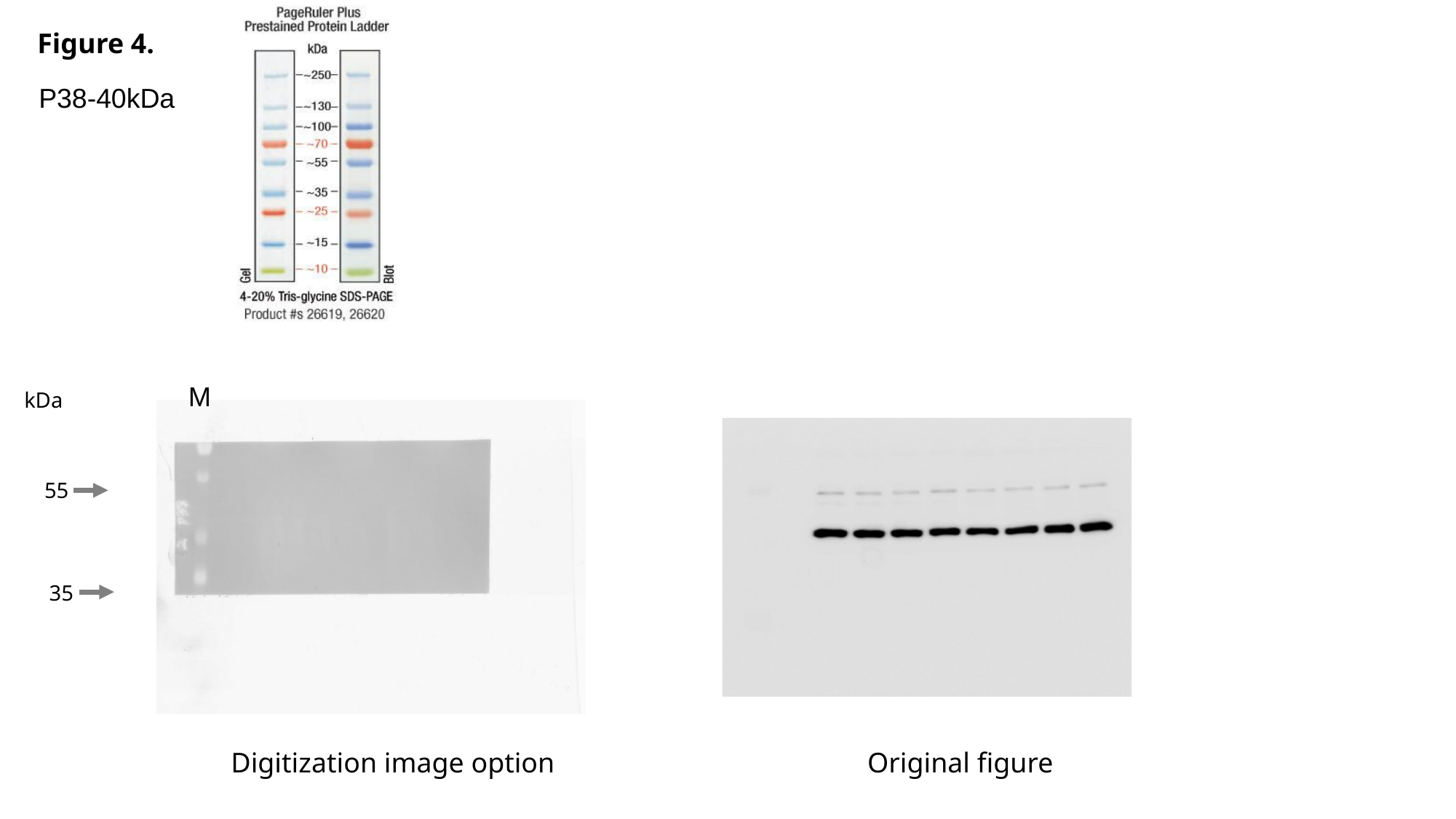

Figure 4.
P38-40kDa
M
kDa
55
35
Digitization image option
Original figure

## Slide 27
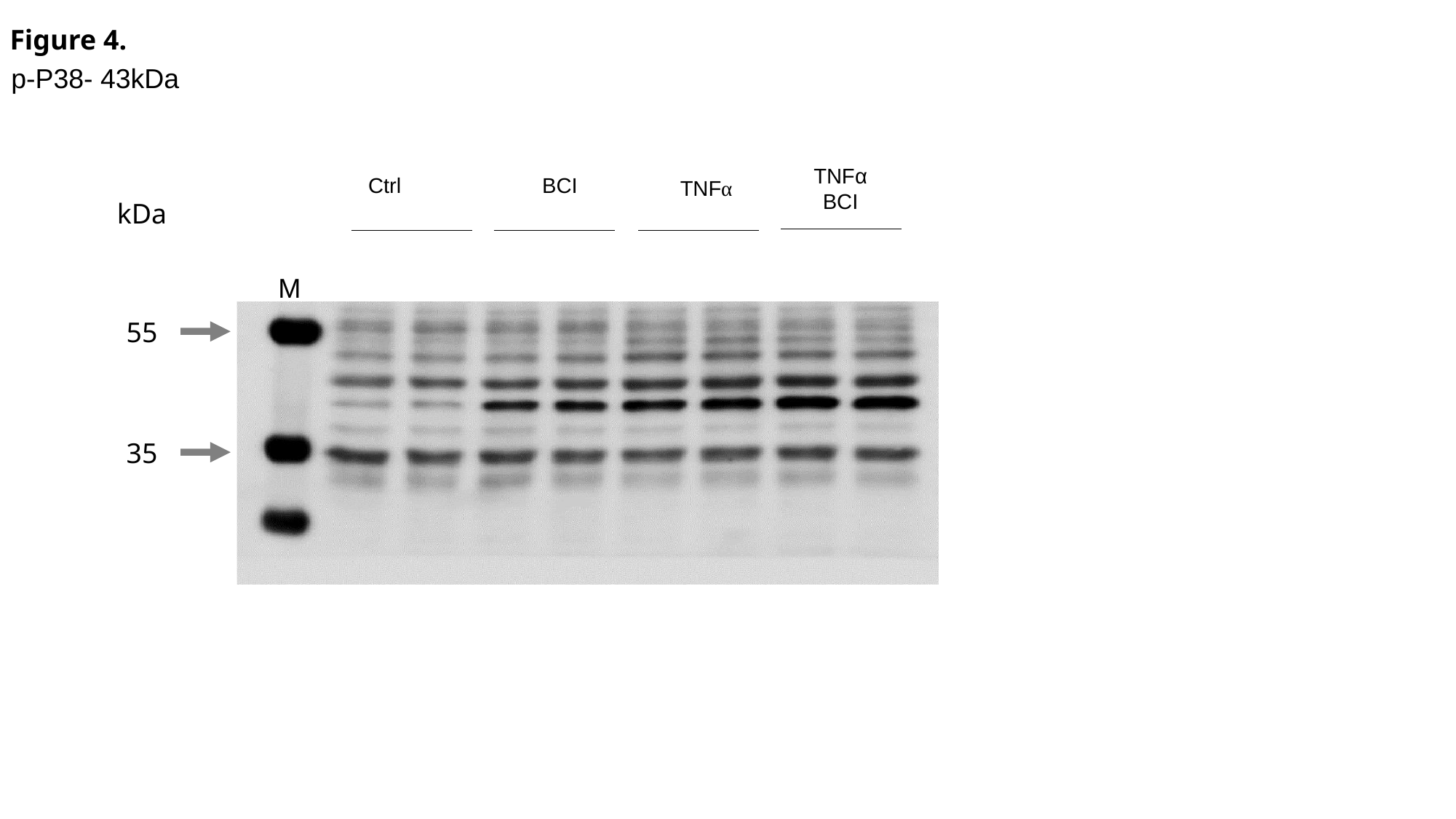

Figure 4.
p-P38- 43kDa
TNFα
BCI
Ctrl
BCI
TNFα
kDa
M
55
35

## Slide 28
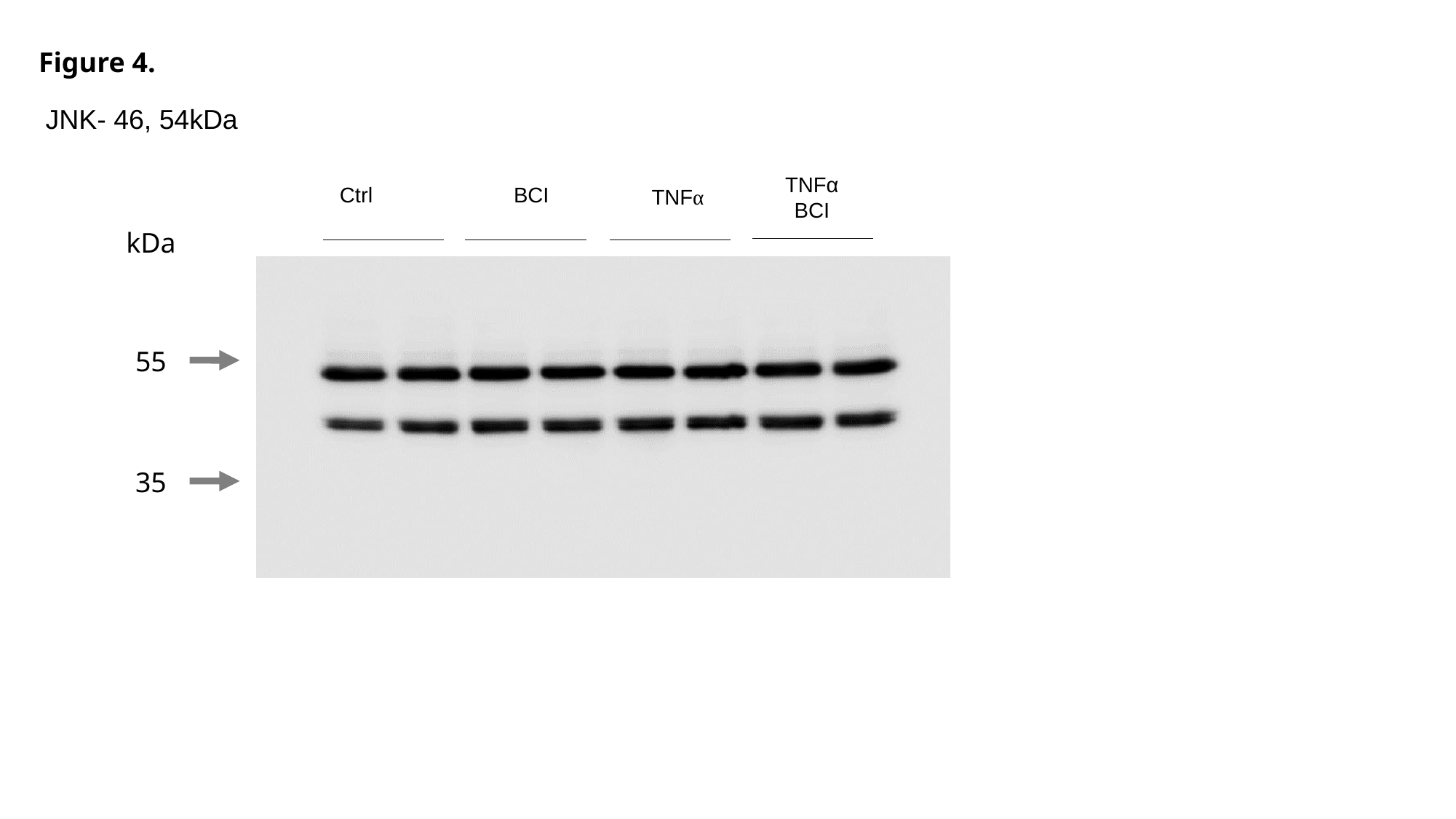

Figure 4.
JNK- 46, 54kDa
TNFα
BCI
Ctrl
BCI
TNFα
kDa
55
35

## Slide 29
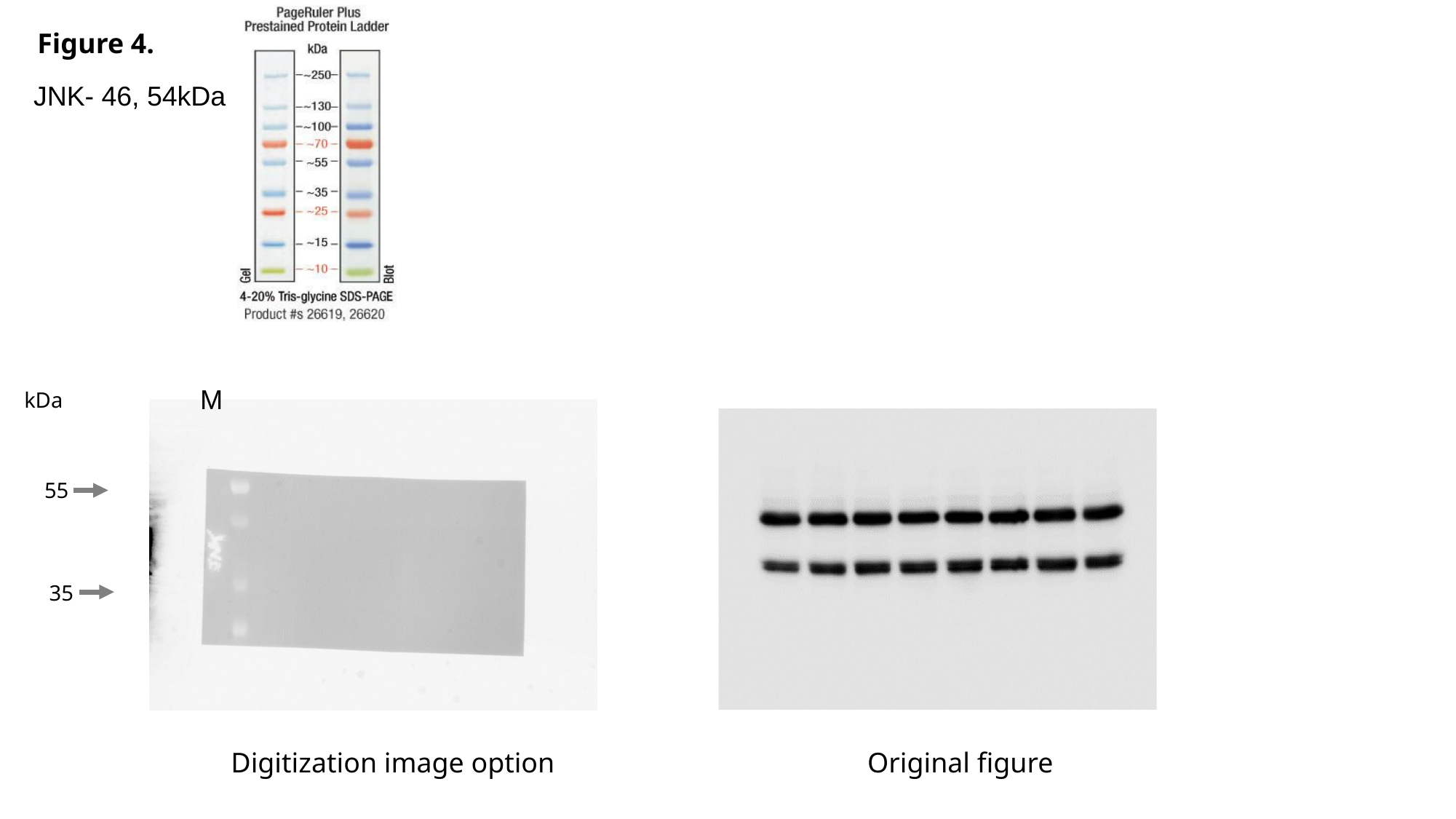

Figure 4.
JNK- 46, 54kDa
M
kDa
55
35
Digitization image option
Original figure

## Slide 30
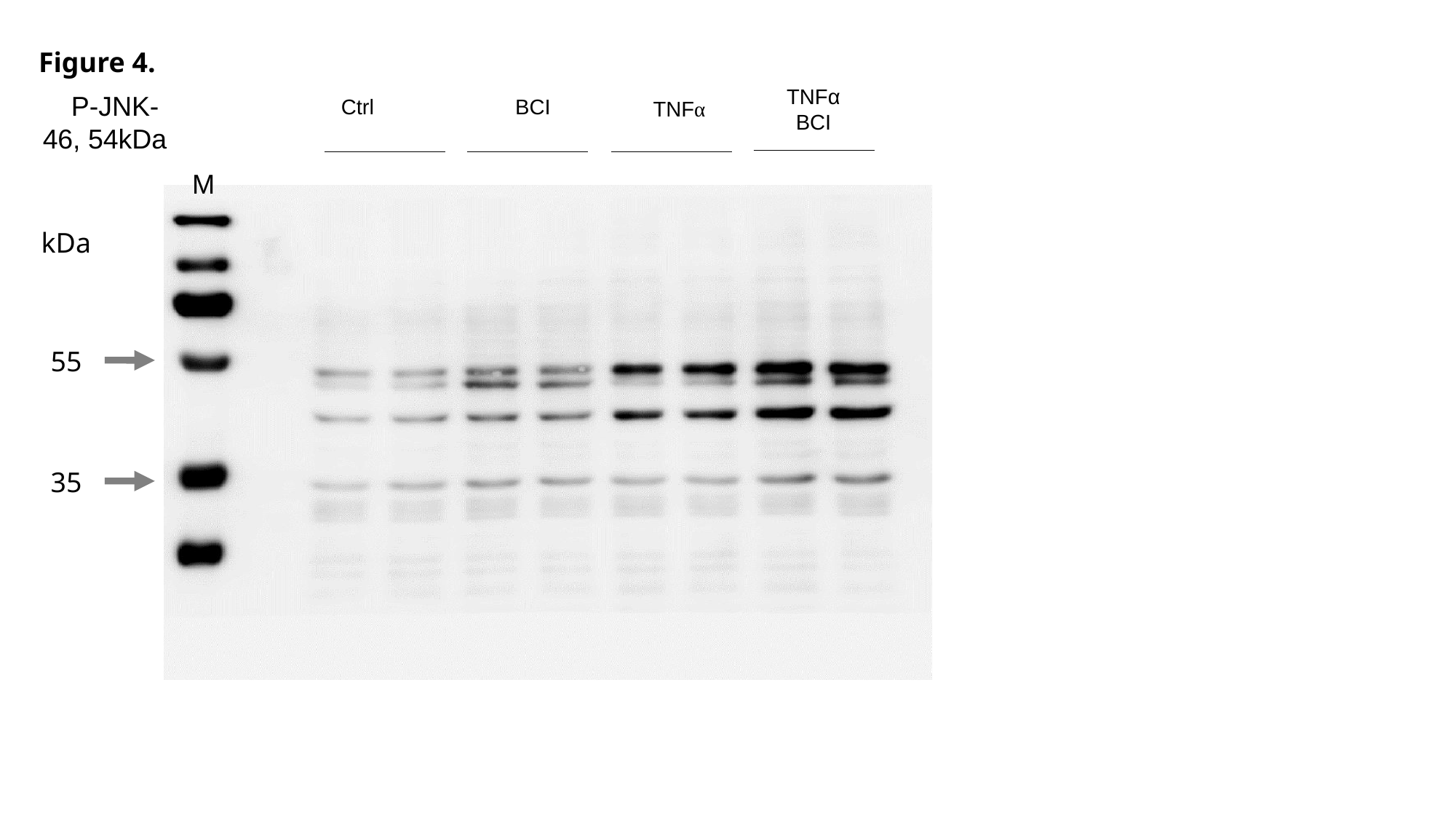

Figure 4.
TNFα
BCI
P-JNK-
46, 54kDa
Ctrl
BCI
TNFα
M
kDa
55
35

## Slide 31
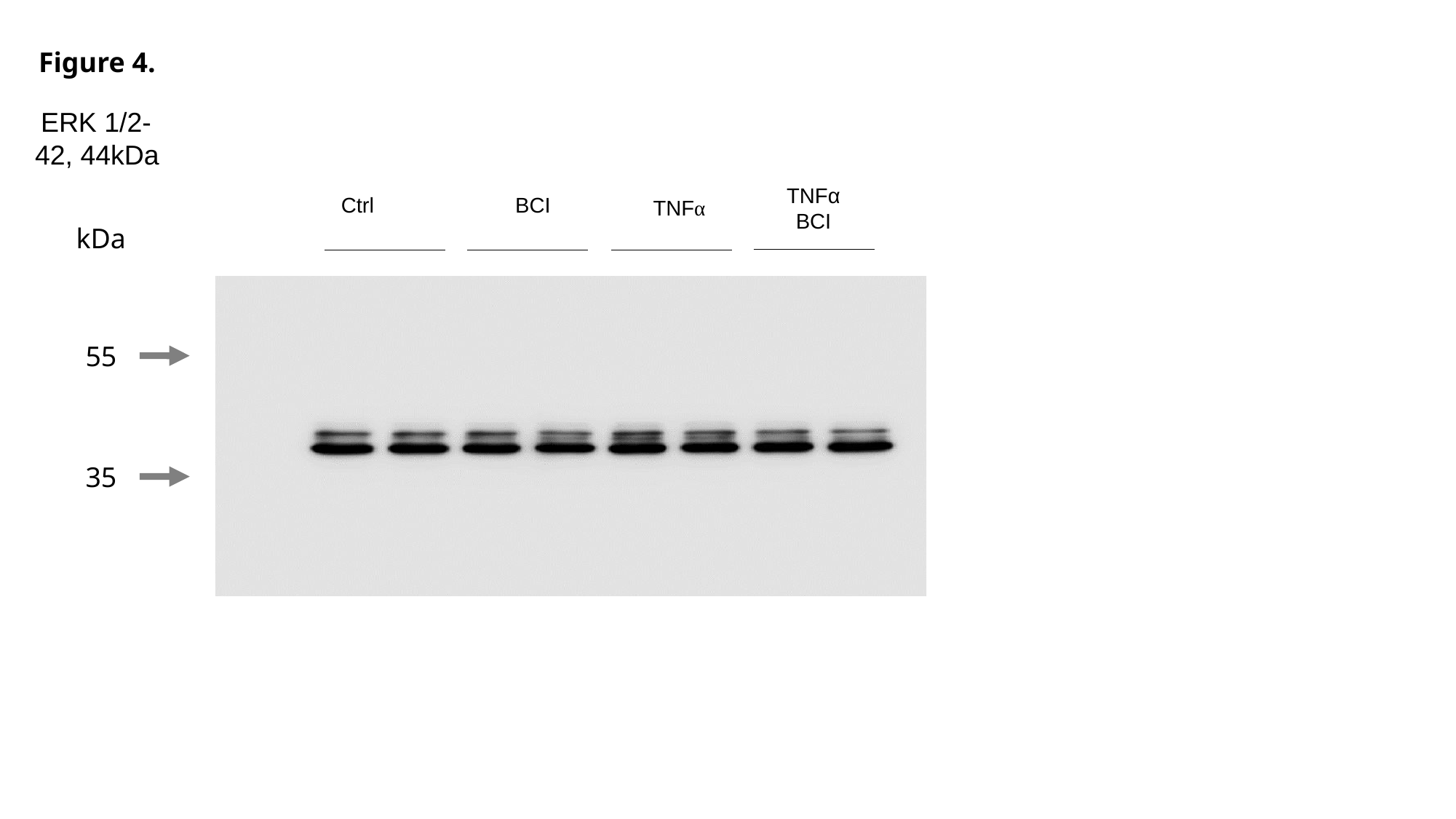

Figure 4.
ERK 1/2-
42, 44kDa
TNFα
BCI
Ctrl
BCI
TNFα
kDa
55
35

## Slide 32
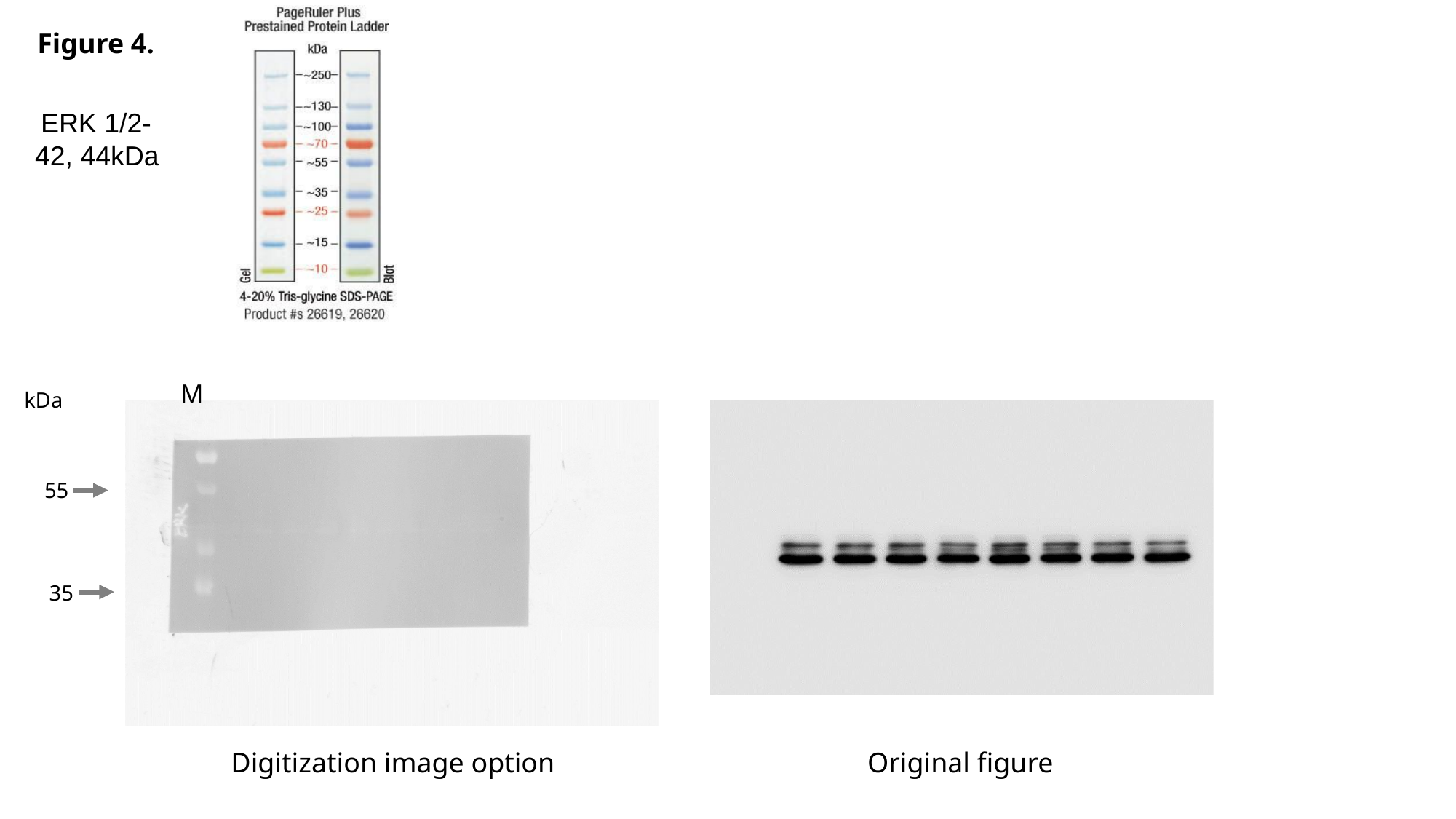

Figure 4.
ERK 1/2-
42, 44kDa
M
kDa
55
35
Digitization image option
Original figure

## Slide 33
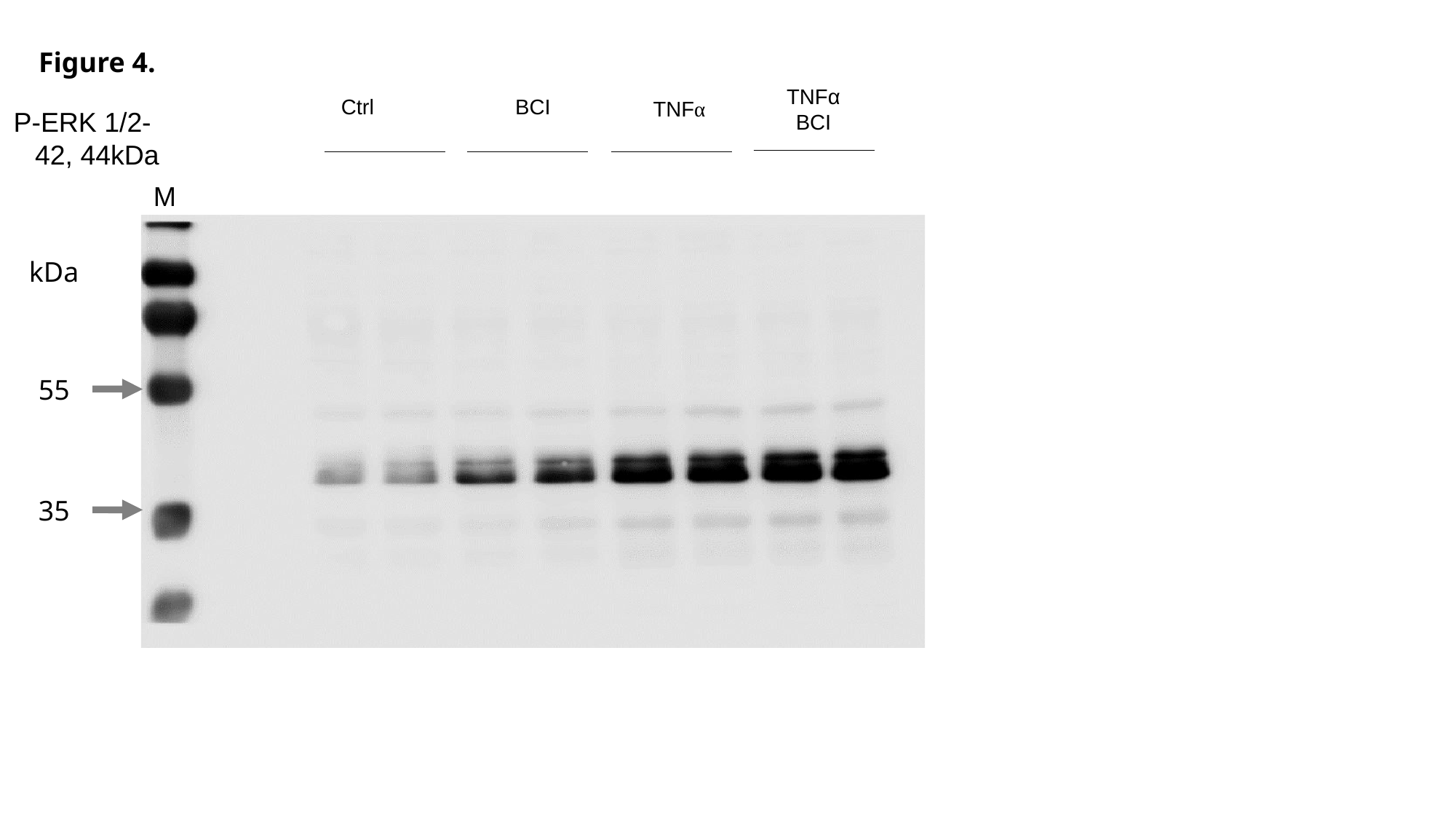

Figure 4.
TNFα
BCI
Ctrl
BCI
TNFα
P-ERK 1/2-
42, 44kDa
M
kDa
55
35

## Slide 34
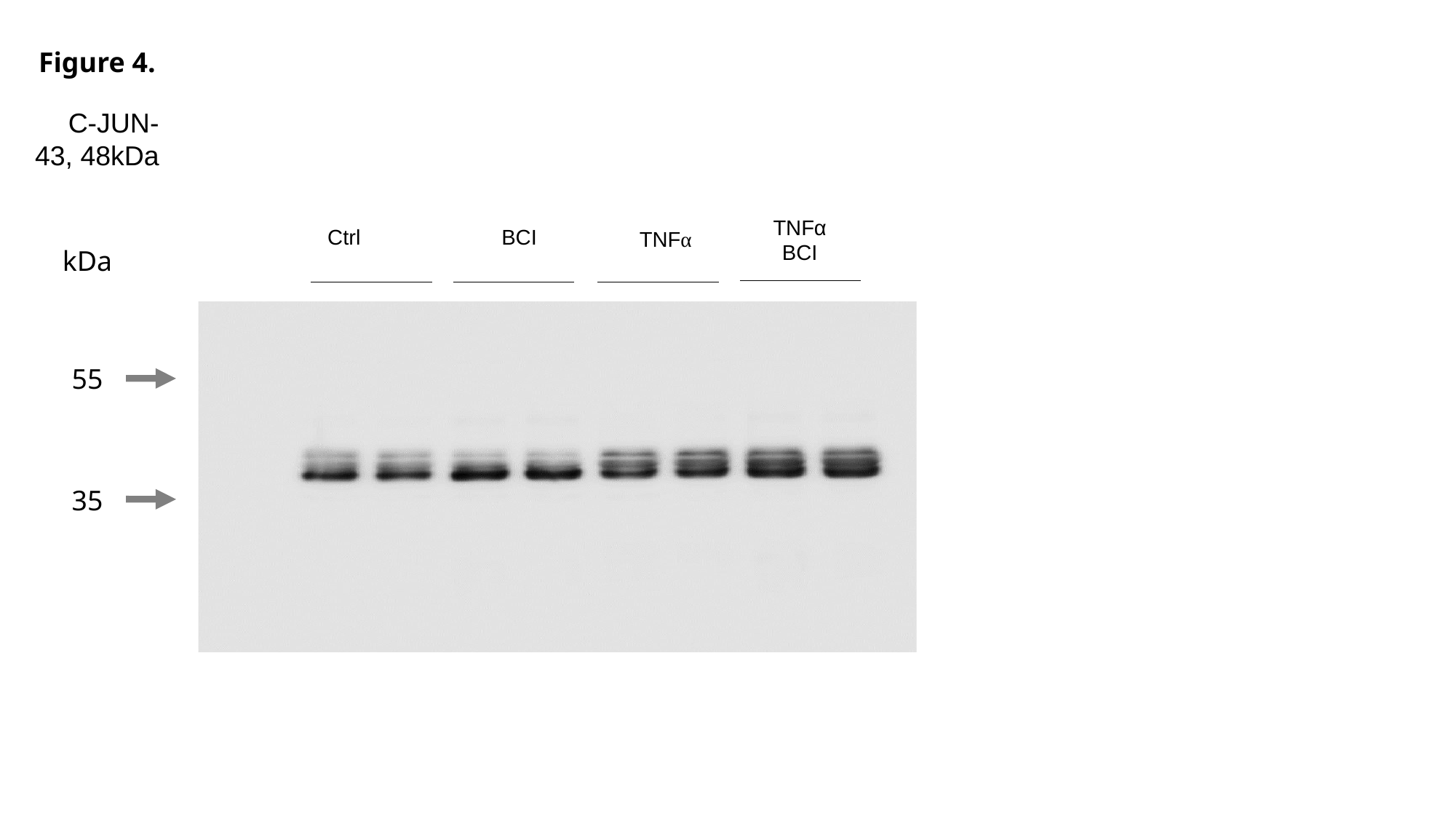

Figure 4.
C-JUN-
43, 48kDa
TNFα
BCI
Ctrl
BCI
TNFα
kDa
55
35

## Slide 35
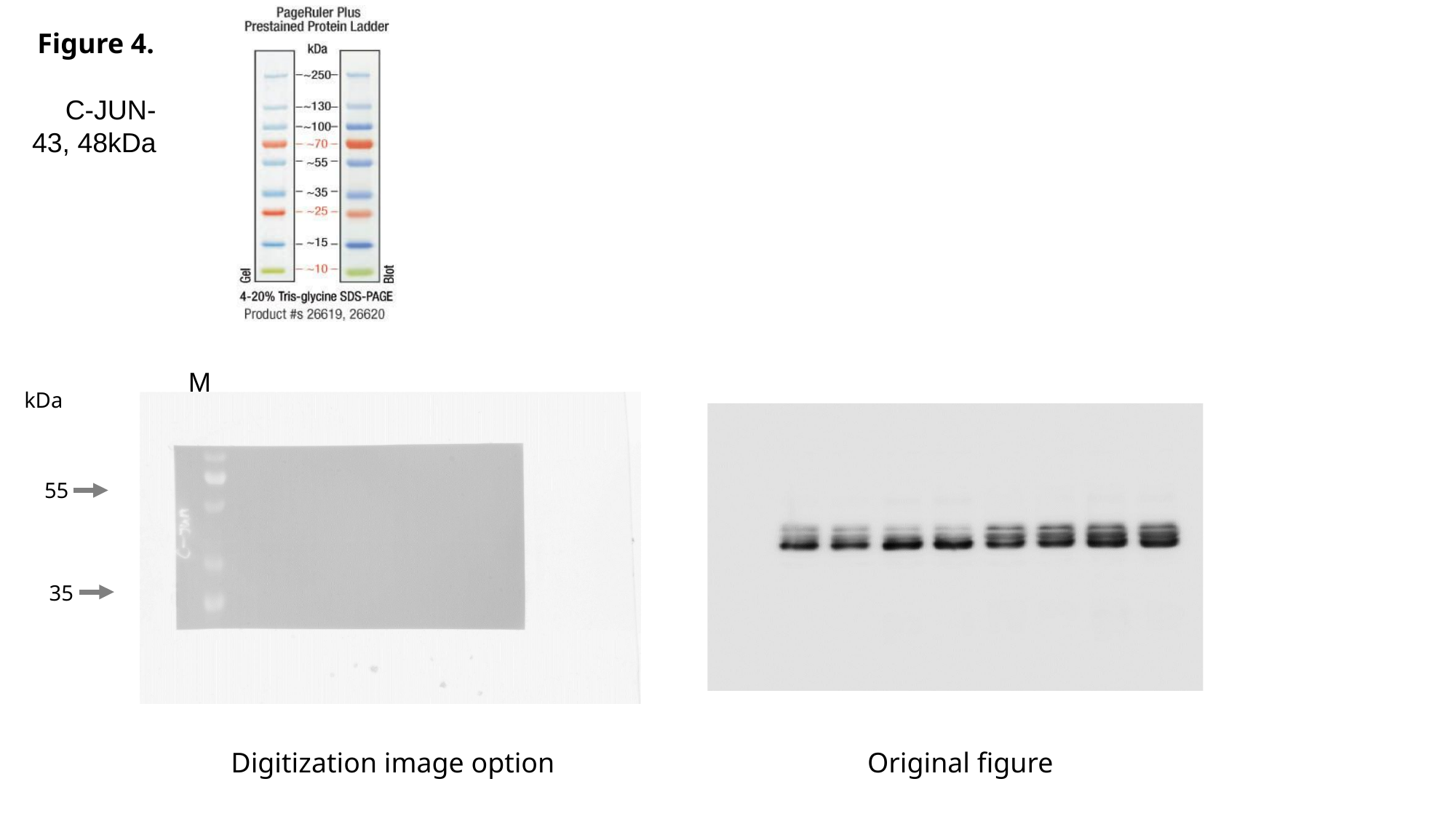

Figure 4.
C-JUN-
43, 48kDa
M
kDa
55
35
Digitization image option
Original figure

## Slide 36
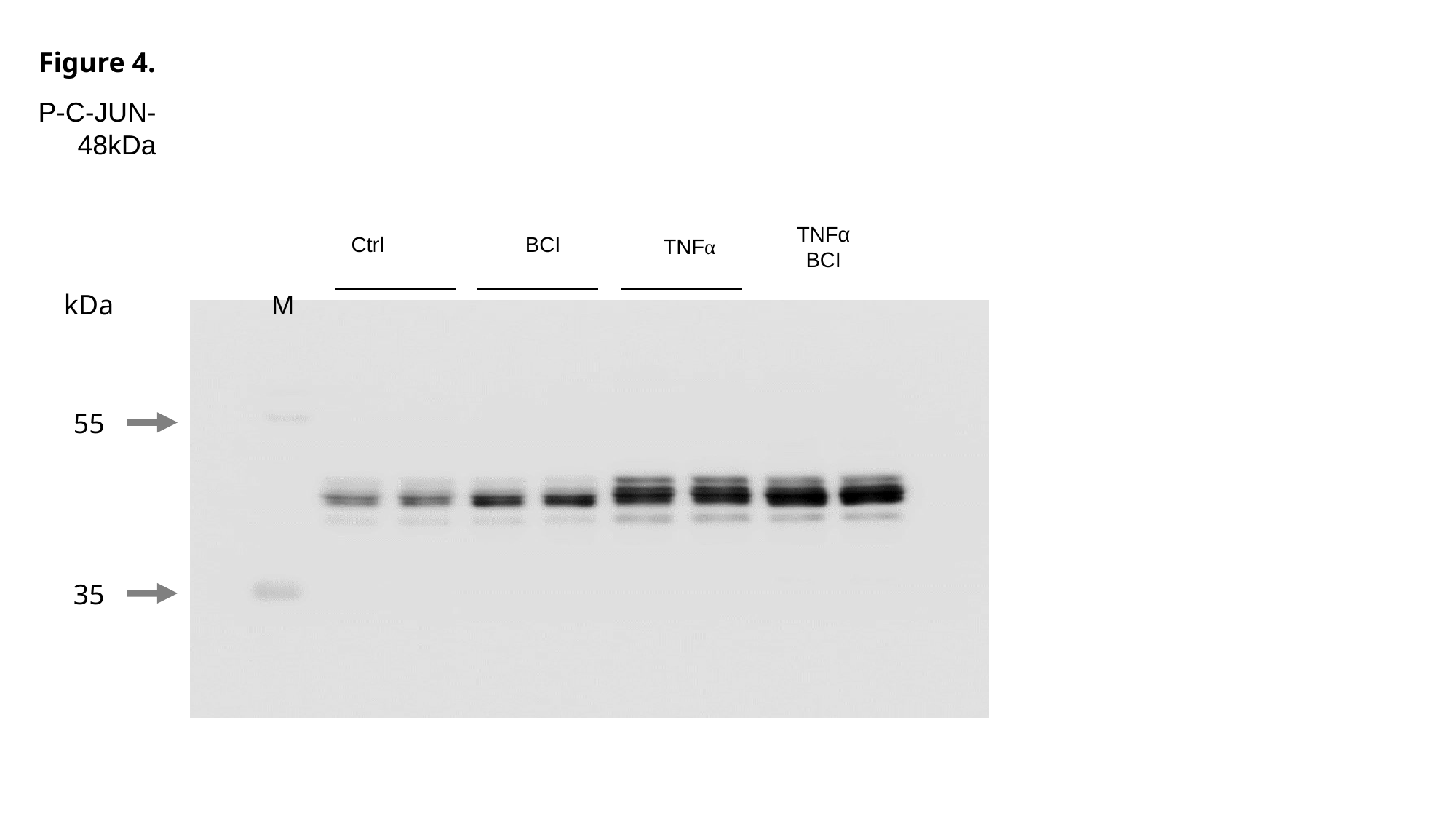

Figure 4.
P-C-JUN- 48kDa
TNFα
BCI
Ctrl
BCI
TNFα
kDa
M
55
35

## Slide 37
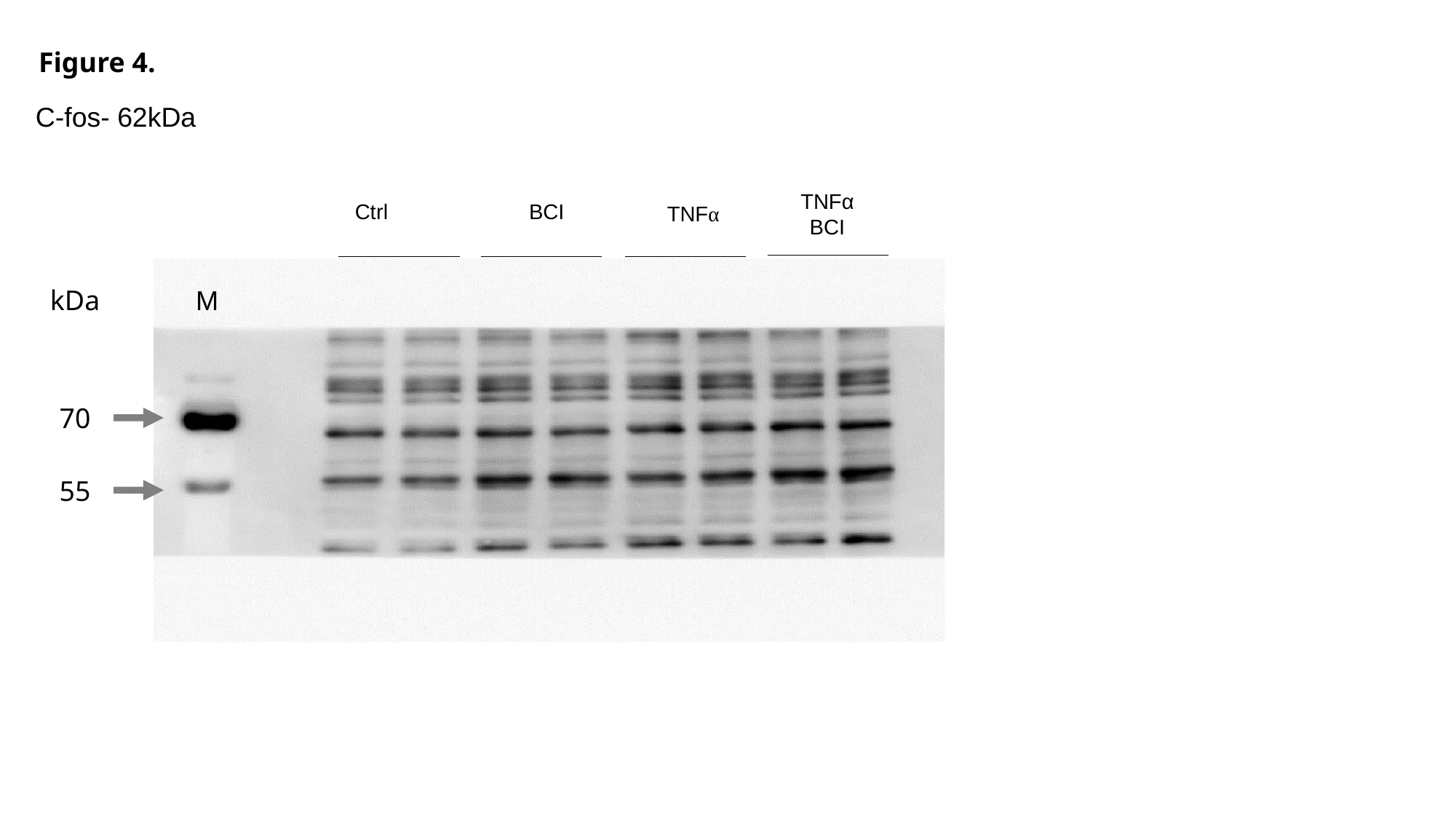

Figure 4.
C-fos- 62kDa
TNFα
BCI
Ctrl
BCI
TNFα
kDa
M
70
55

## Slide 38
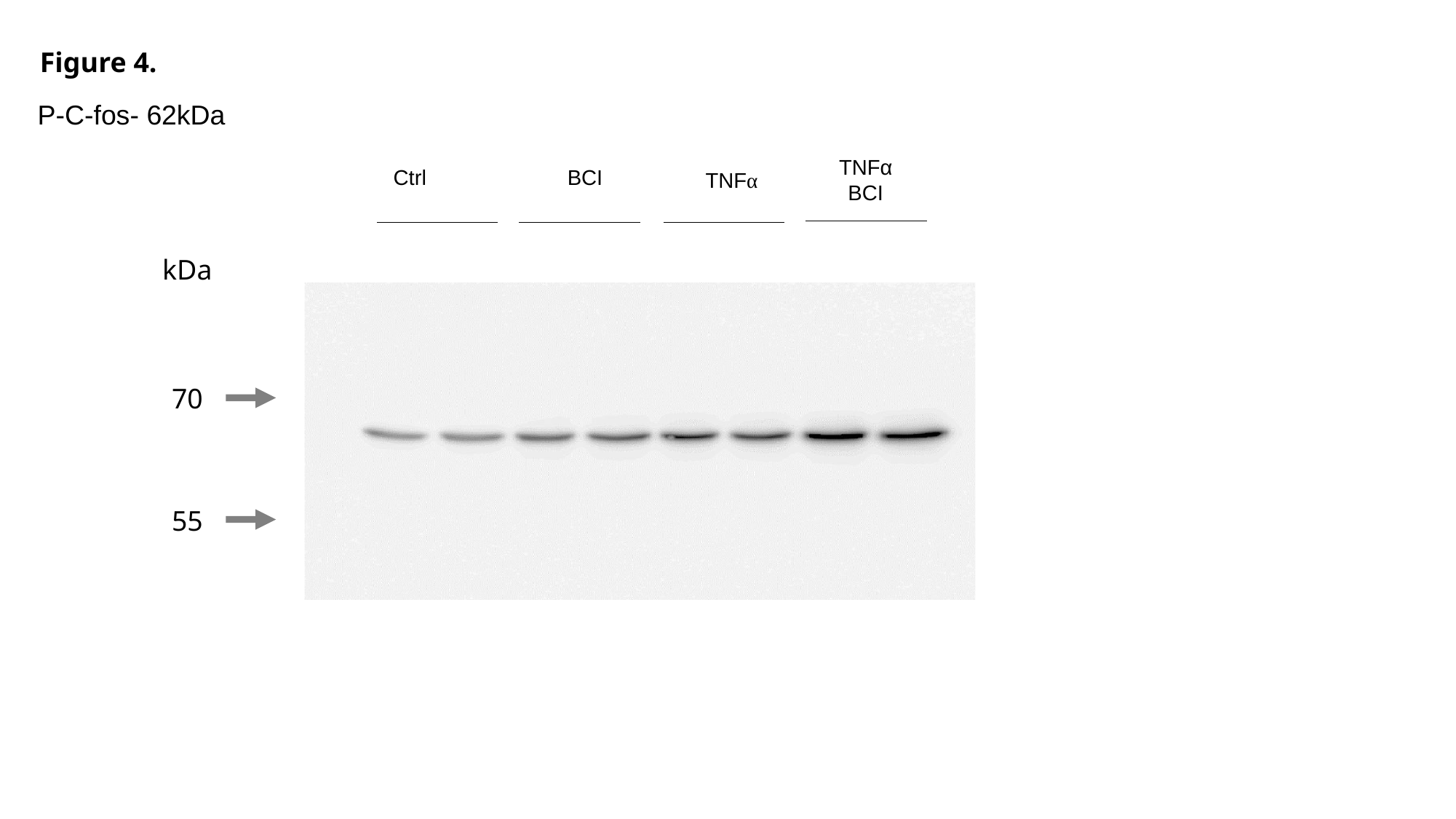

Figure 4.
P-C-fos- 62kDa
TNFα
BCI
Ctrl
BCI
TNFα
kDa
70
55

## Slide 39
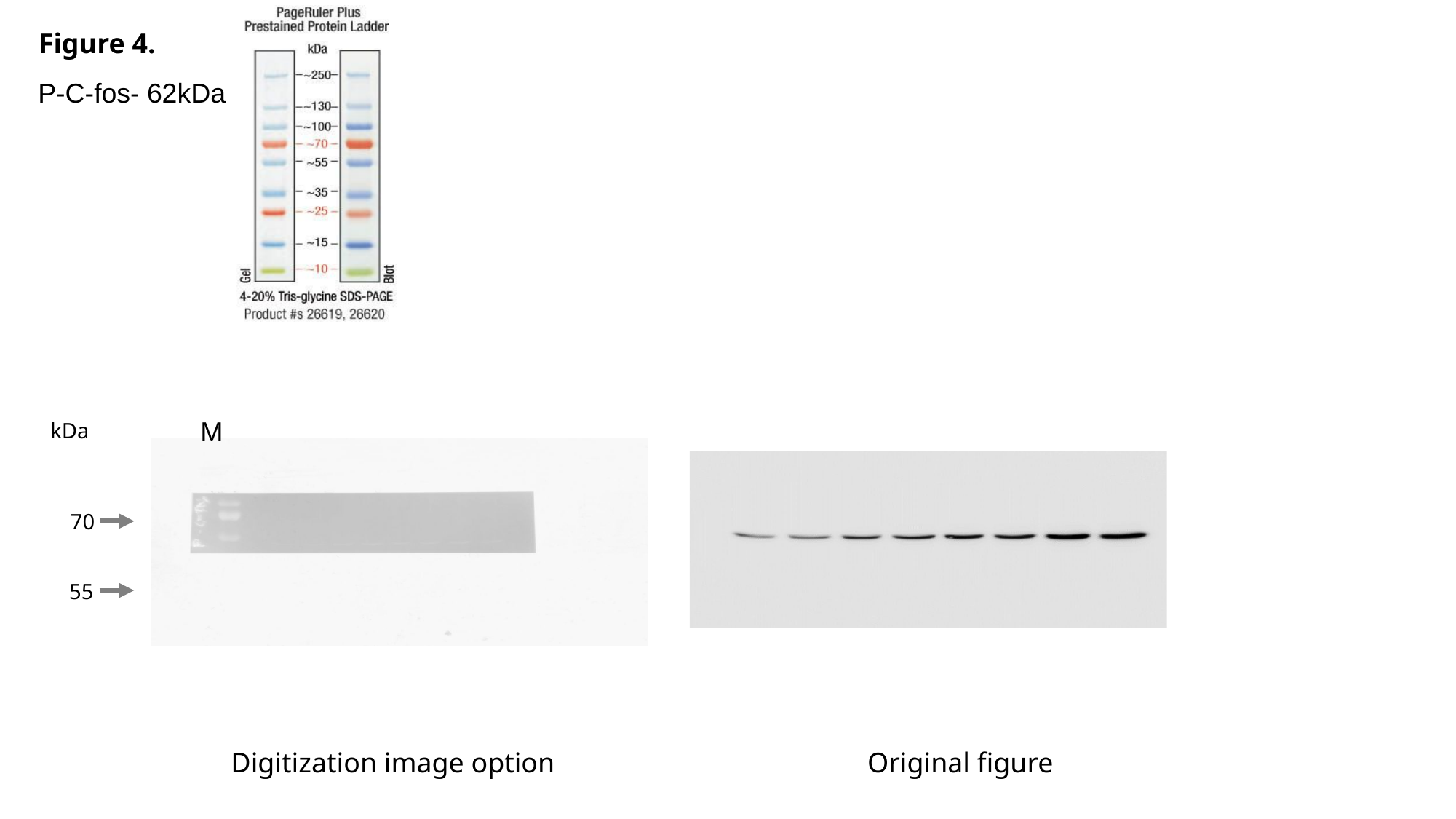

Figure 4.
P-C-fos- 62kDa
M
kDa
70
55
Digitization image option
Original figure

## Slide 40
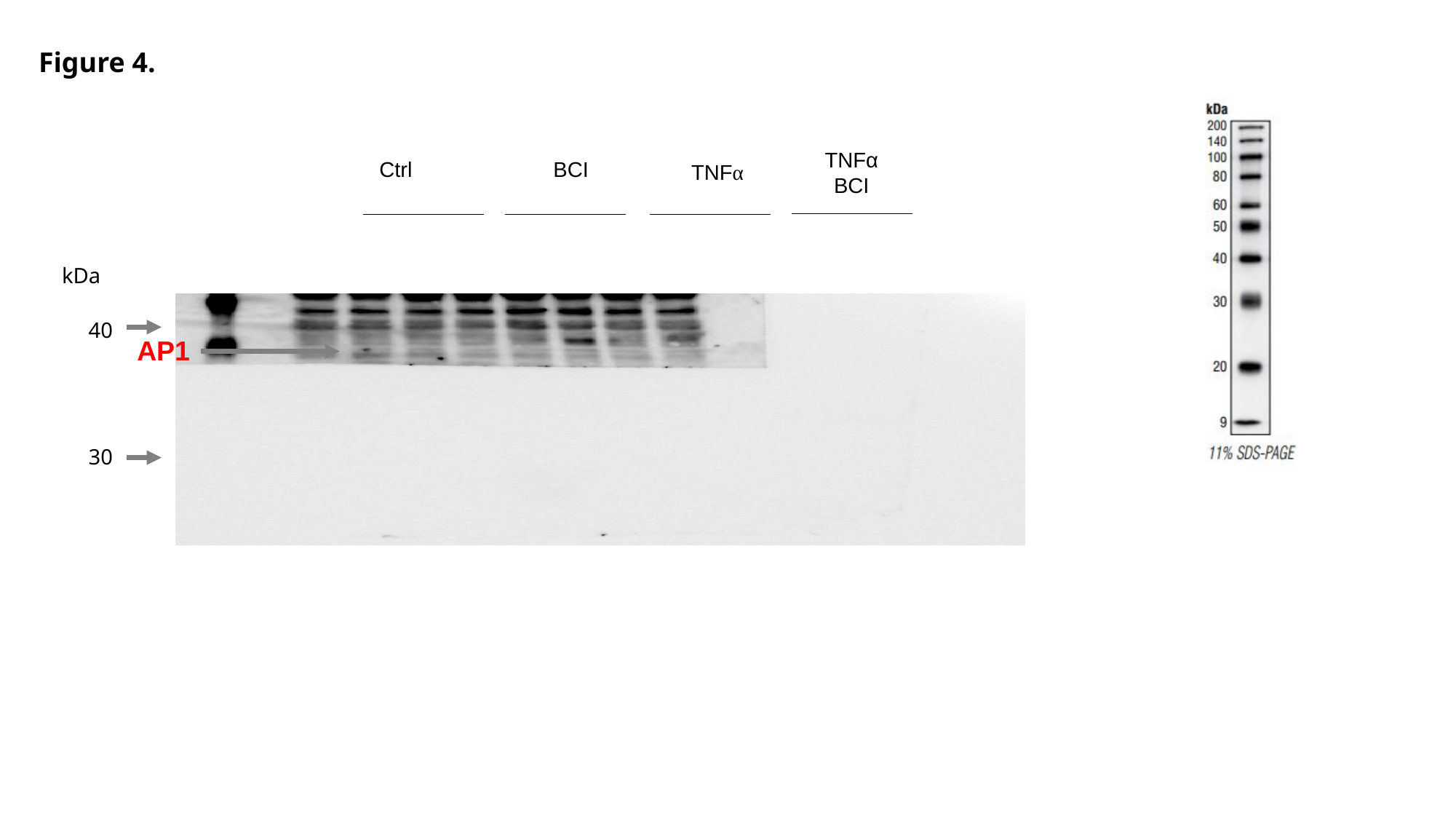

Figure 4.
TNFα
BCI
Ctrl
BCI
TNFα
kDa
40
AP1
30

## Slide 41
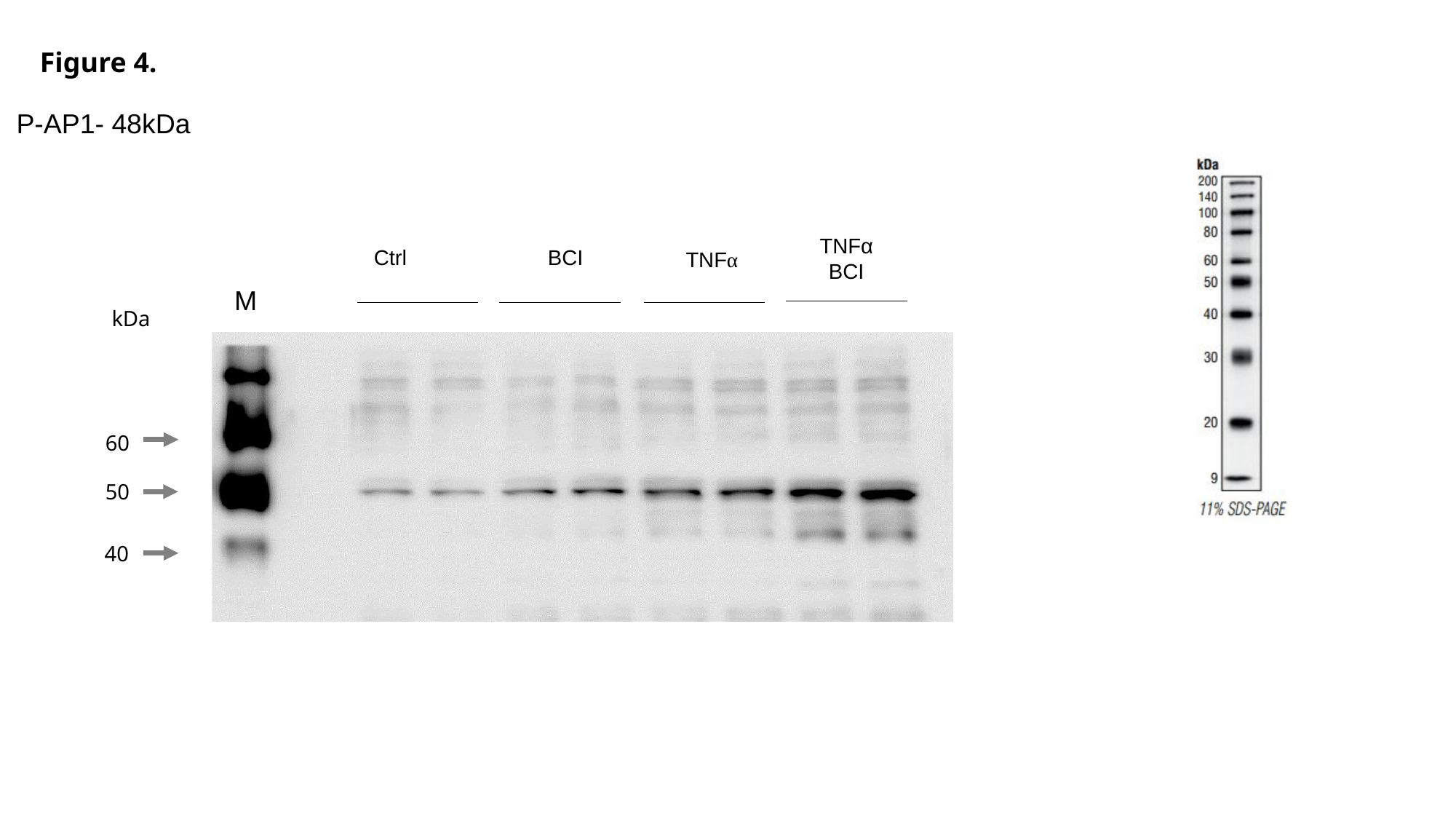

Figure 4.
P-AP1- 48kDa
TNFα
BCI
Ctrl
BCI
TNFα
M
kDa
60
50
40

## Slide 42
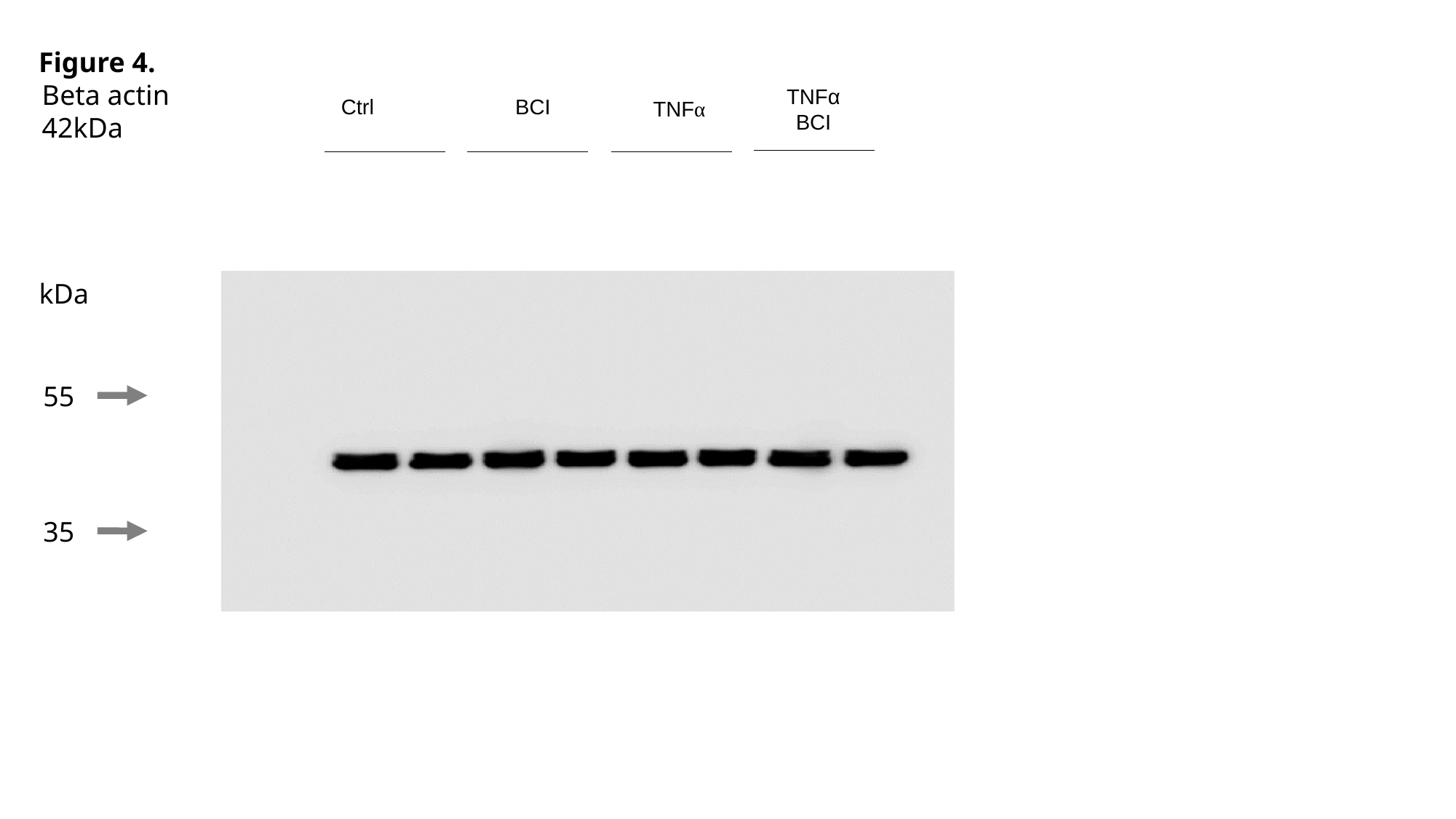

Figure 4.
Beta actin
42kDa
TNFα
BCI
Ctrl
BCI
TNFα
kDa
55
35

## Slide 43
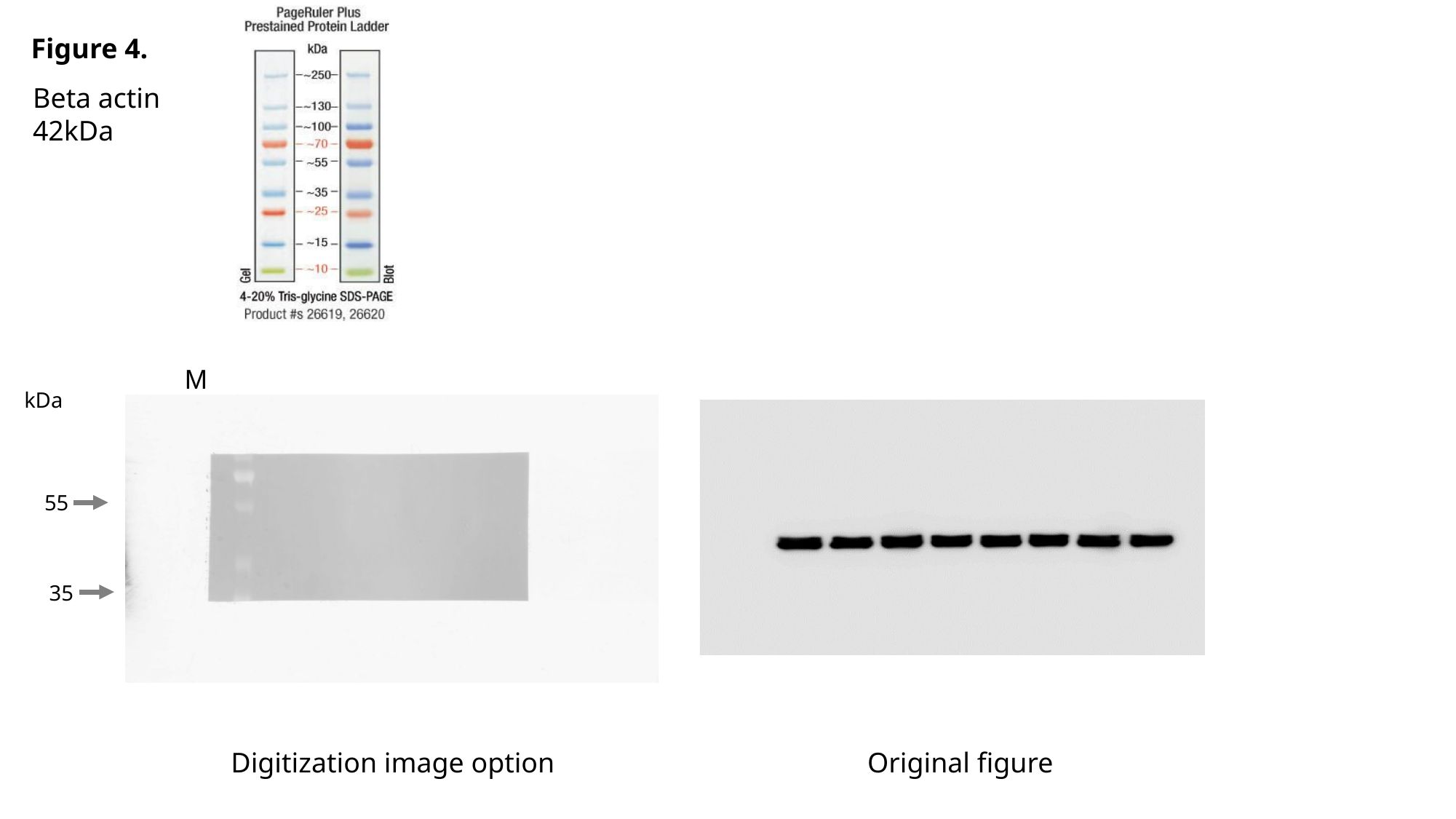

Figure 4.
Beta actin
42kDa
M
kDa
55
35
Digitization image option
Original figure

## Slide 44
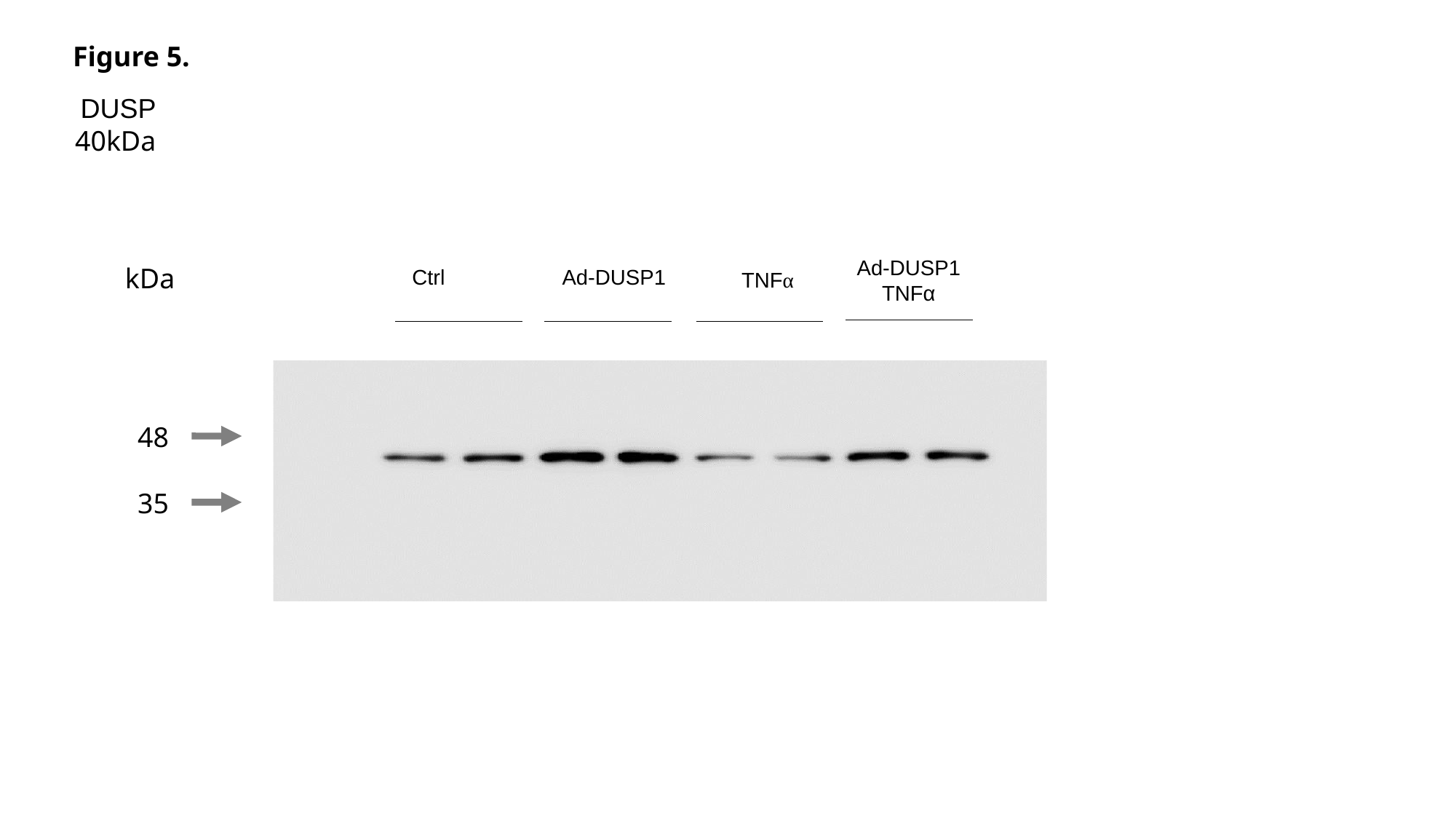

Figure 5.
DUSP
40kDa
Ad-DUSP1
TNFα
kDa
Ctrl
Ad-DUSP1
TNFα
48
35

## Slide 45
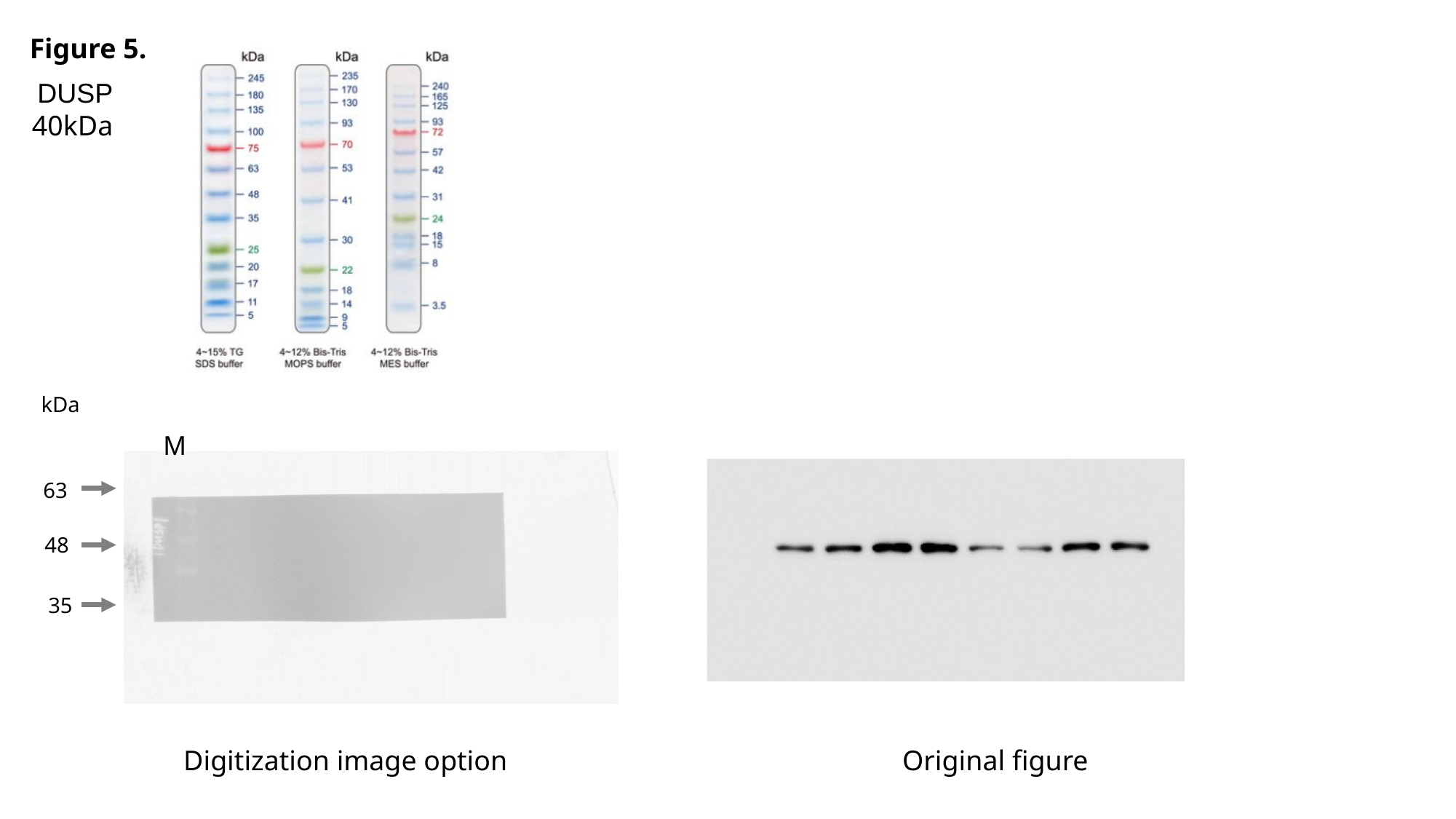

Figure 5.
DUSP
40kDa
kDa
M
63
48
35
Digitization image option
Original figure

## Slide 46
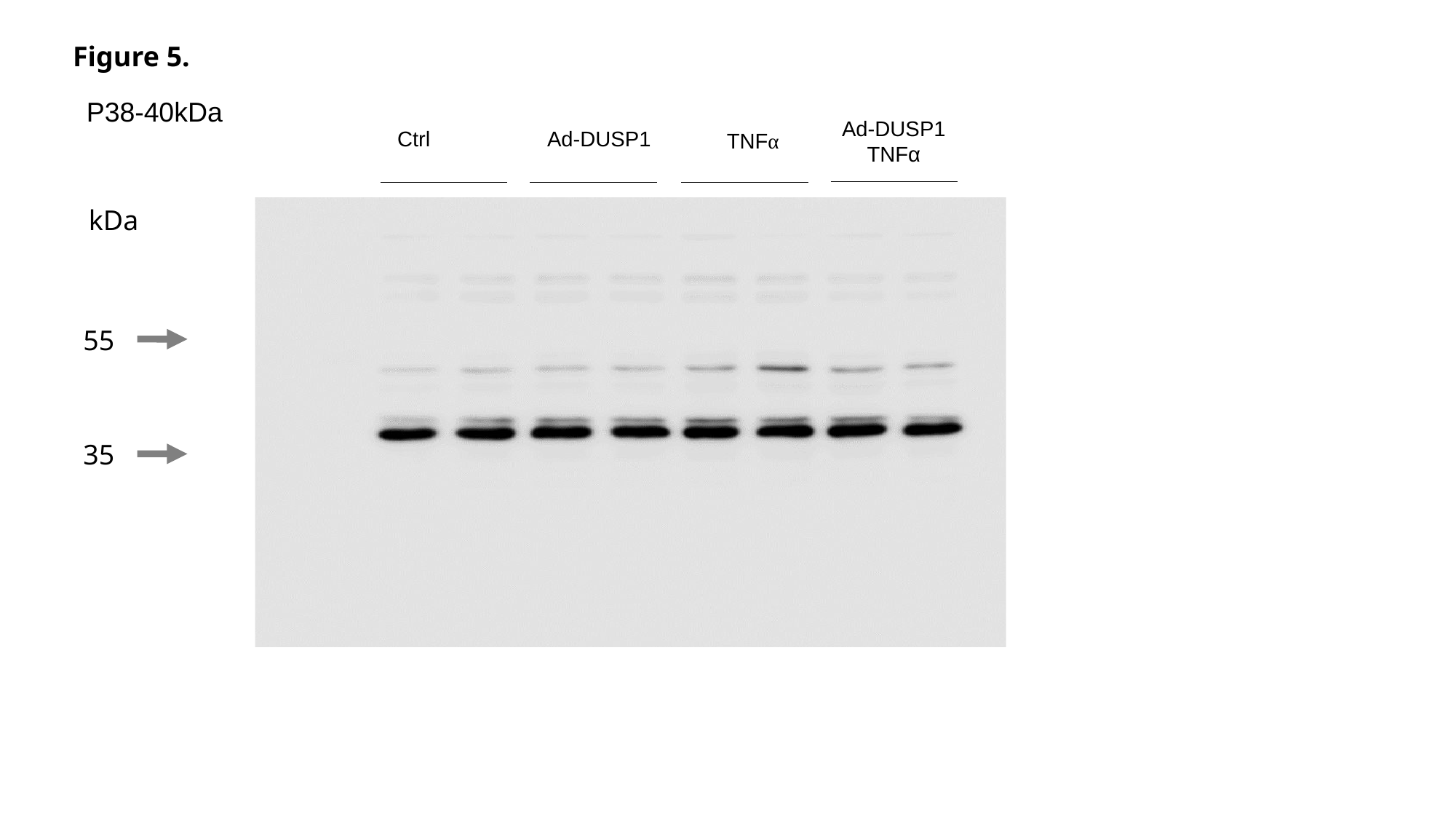

Figure 5.
P38-40kDa
Ad-DUSP1
TNFα
Ctrl
Ad-DUSP1
TNFα
kDa
55
35

## Slide 47
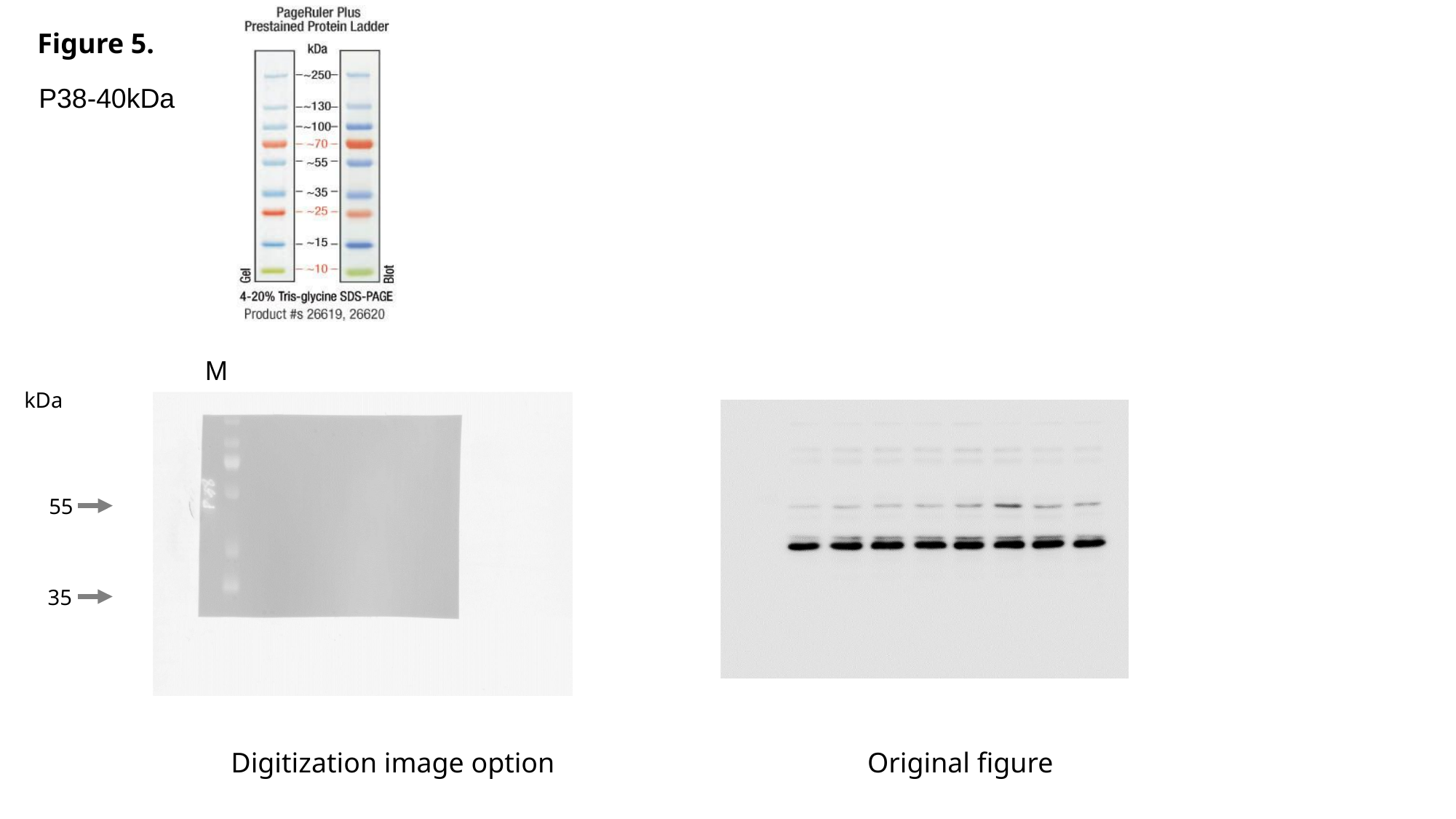

Figure 5.
P38-40kDa
M
kDa
55
35
Digitization image option
Original figure

## Slide 48
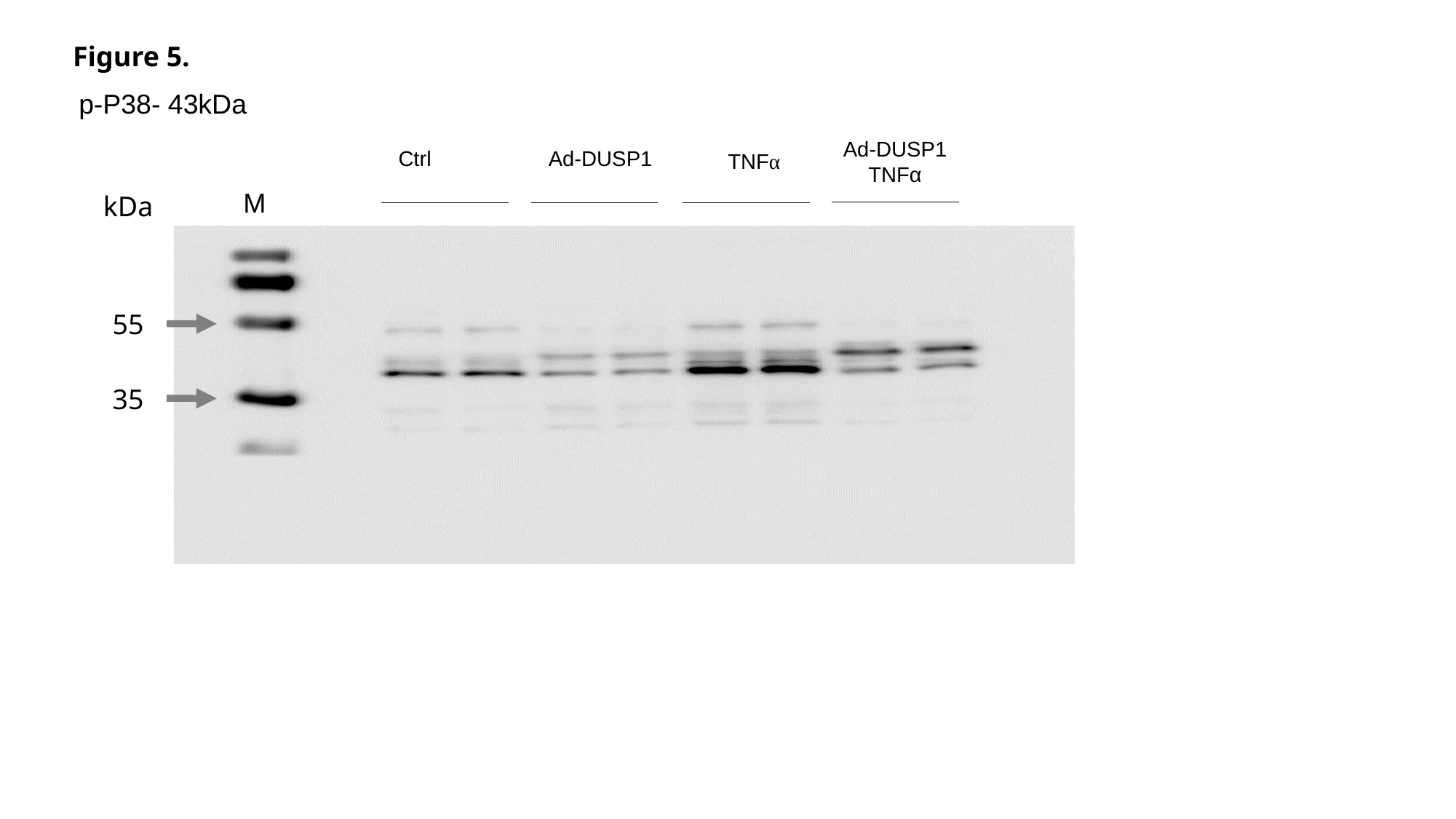

Figure 5.
p-P38- 43kDa
Ad-DUSP1
TNFα
Ctrl
Ad-DUSP1
TNFα
M
kDa
55
35

## Slide 49
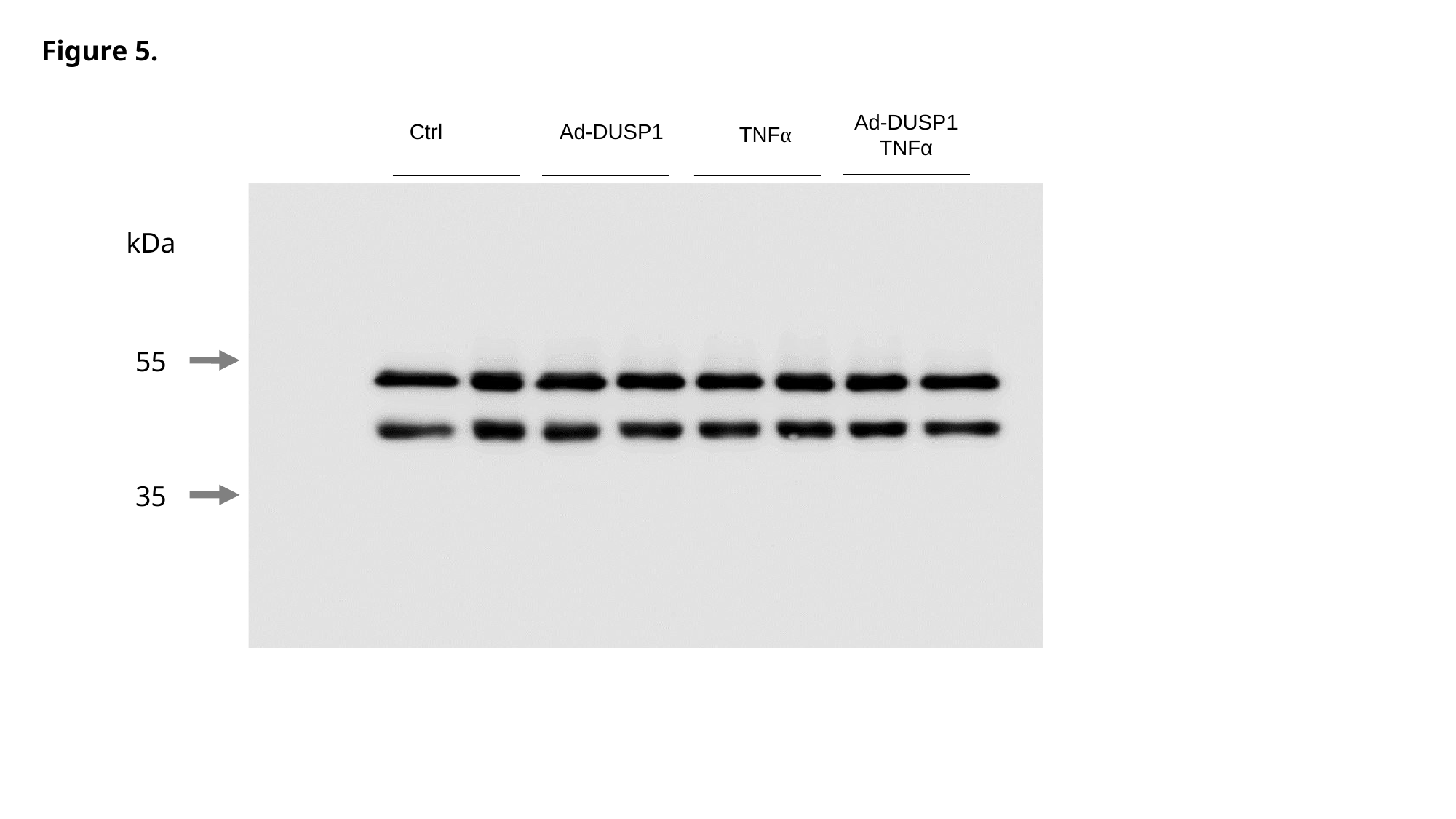

Figure 5.
Ad-DUSP1
TNFα
Ctrl
Ad-DUSP1
TNFα
kDa
55
35

## Slide 50
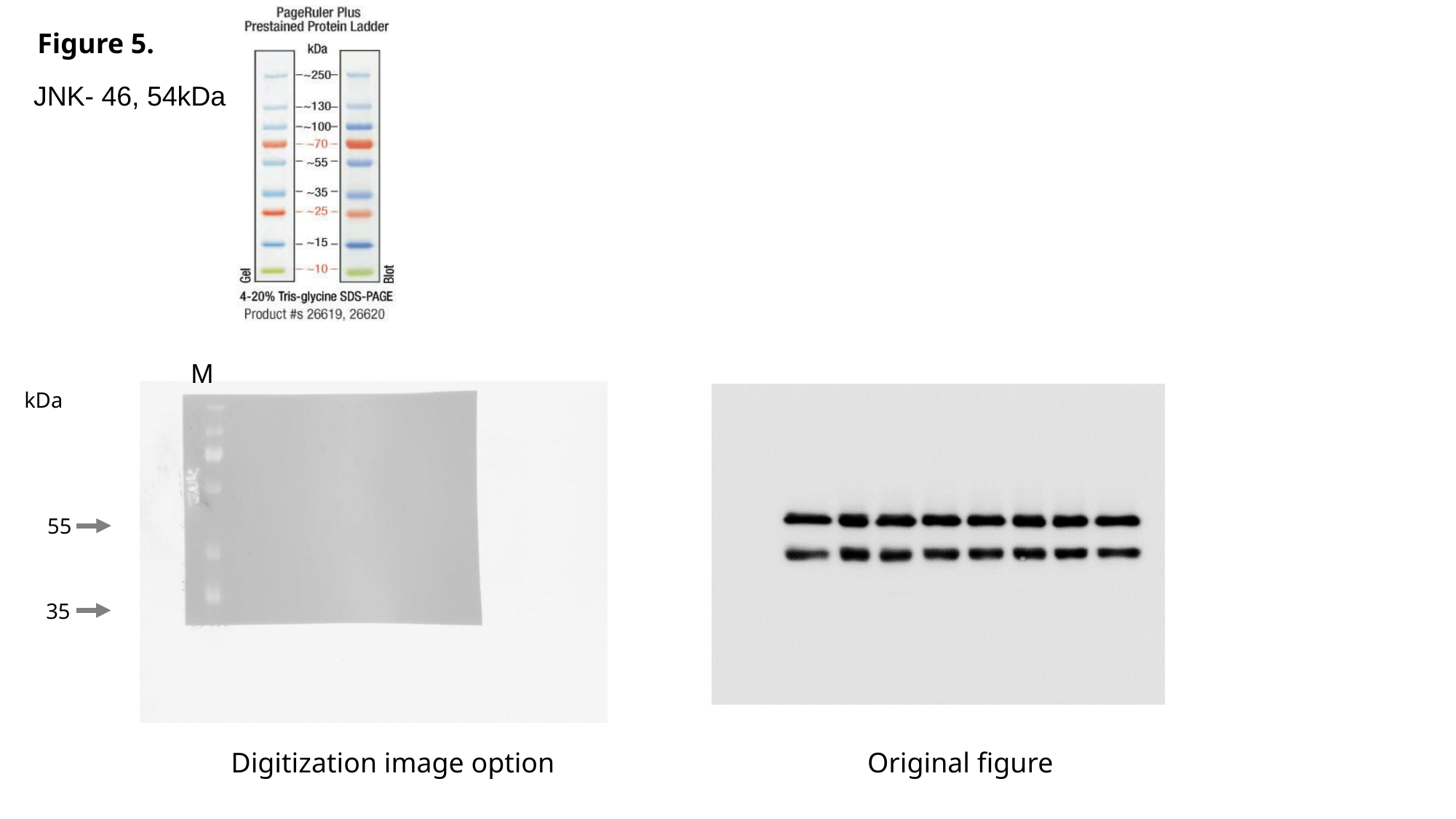

Figure 5.
JNK- 46, 54kDa
M
kDa
55
35
Digitization image option
Original figure

## Slide 51
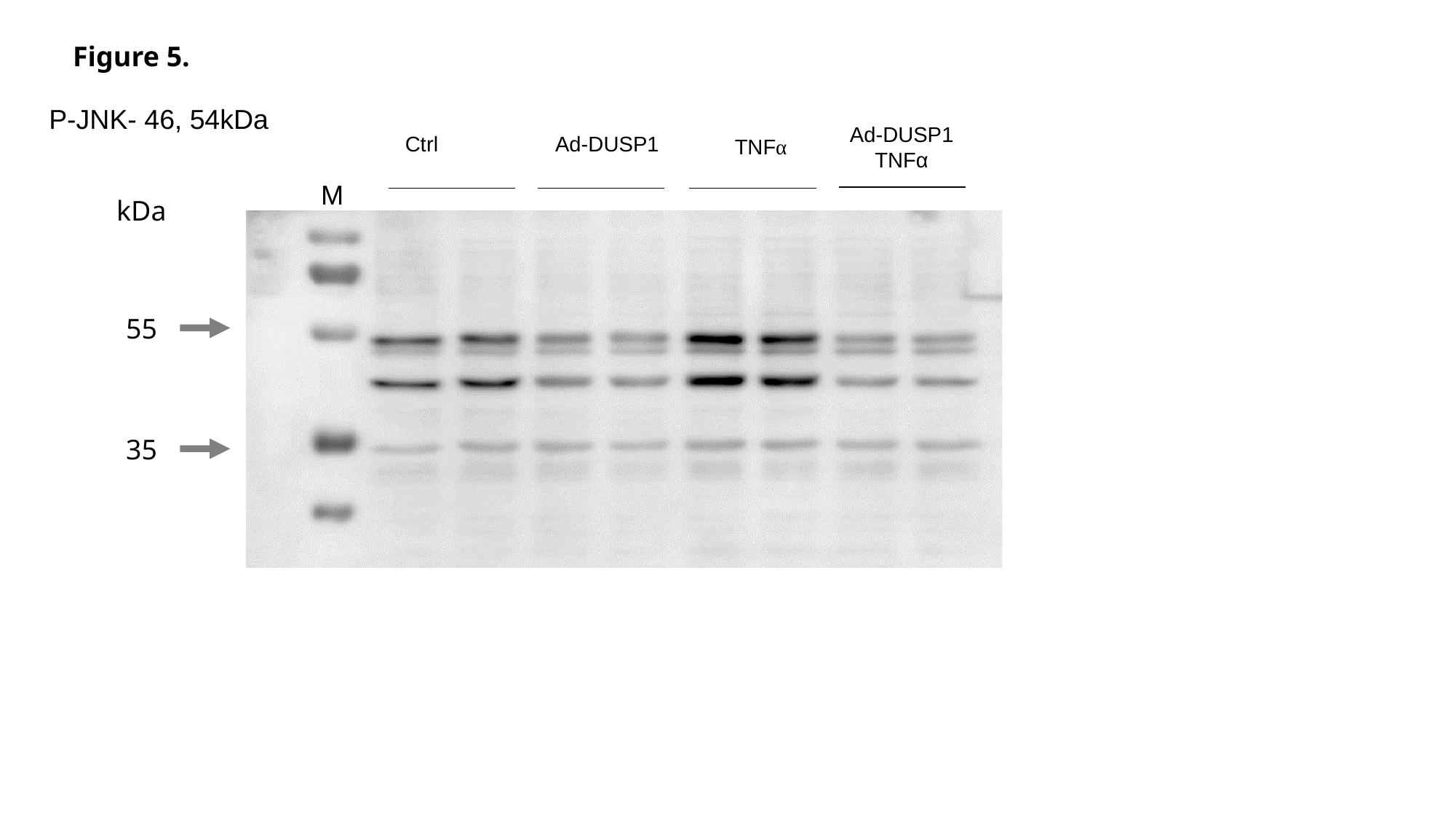

Figure 5.
P-JNK- 46, 54kDa
Ad-DUSP1
TNFα
Ctrl
Ad-DUSP1
TNFα
M
kDa
55
35

## Slide 52
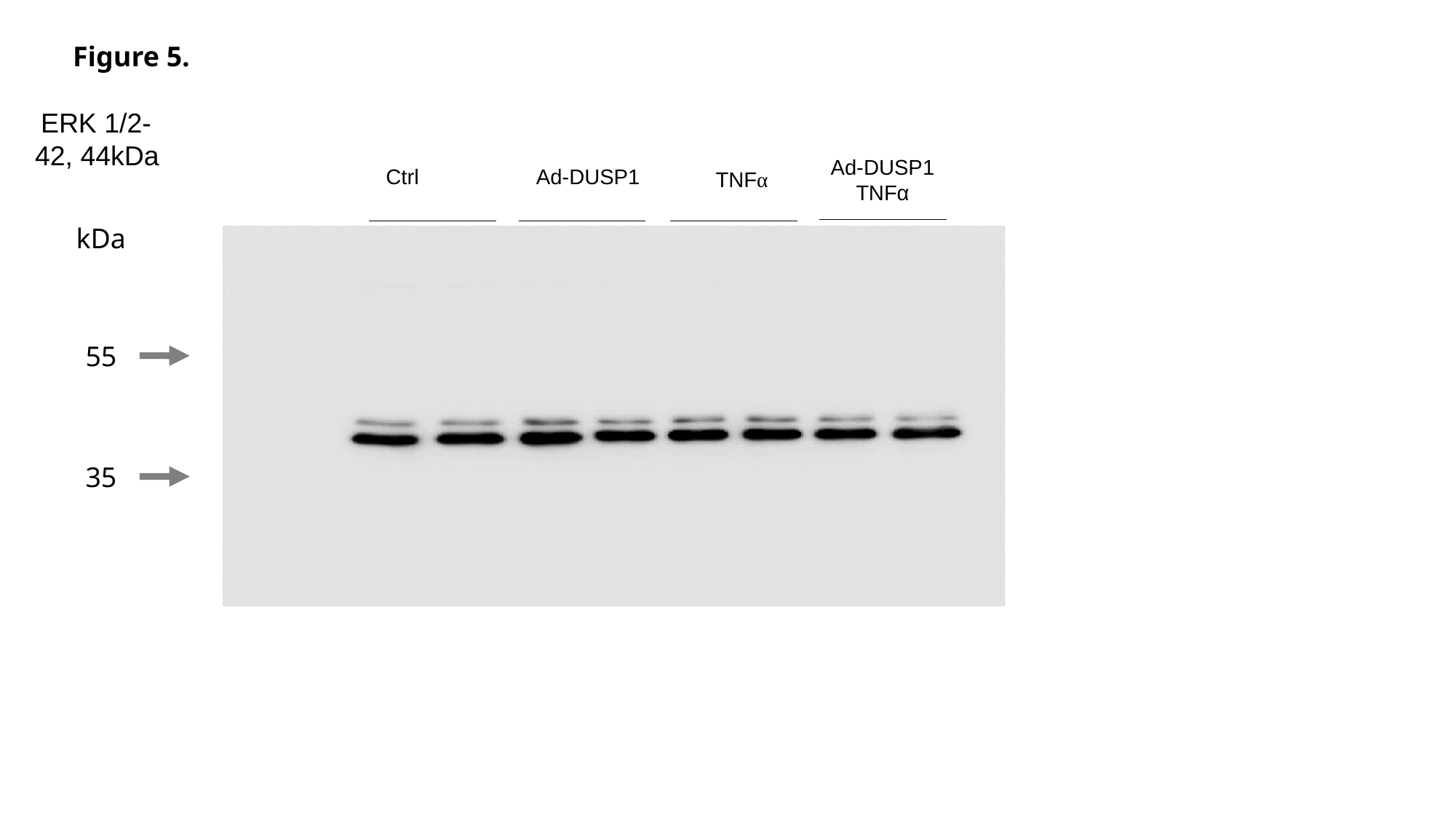

Figure 5.
ERK 1/2-
42, 44kDa
Ad-DUSP1
TNFα
Ctrl
Ad-DUSP1
TNFα
kDa
55
35

## Slide 53
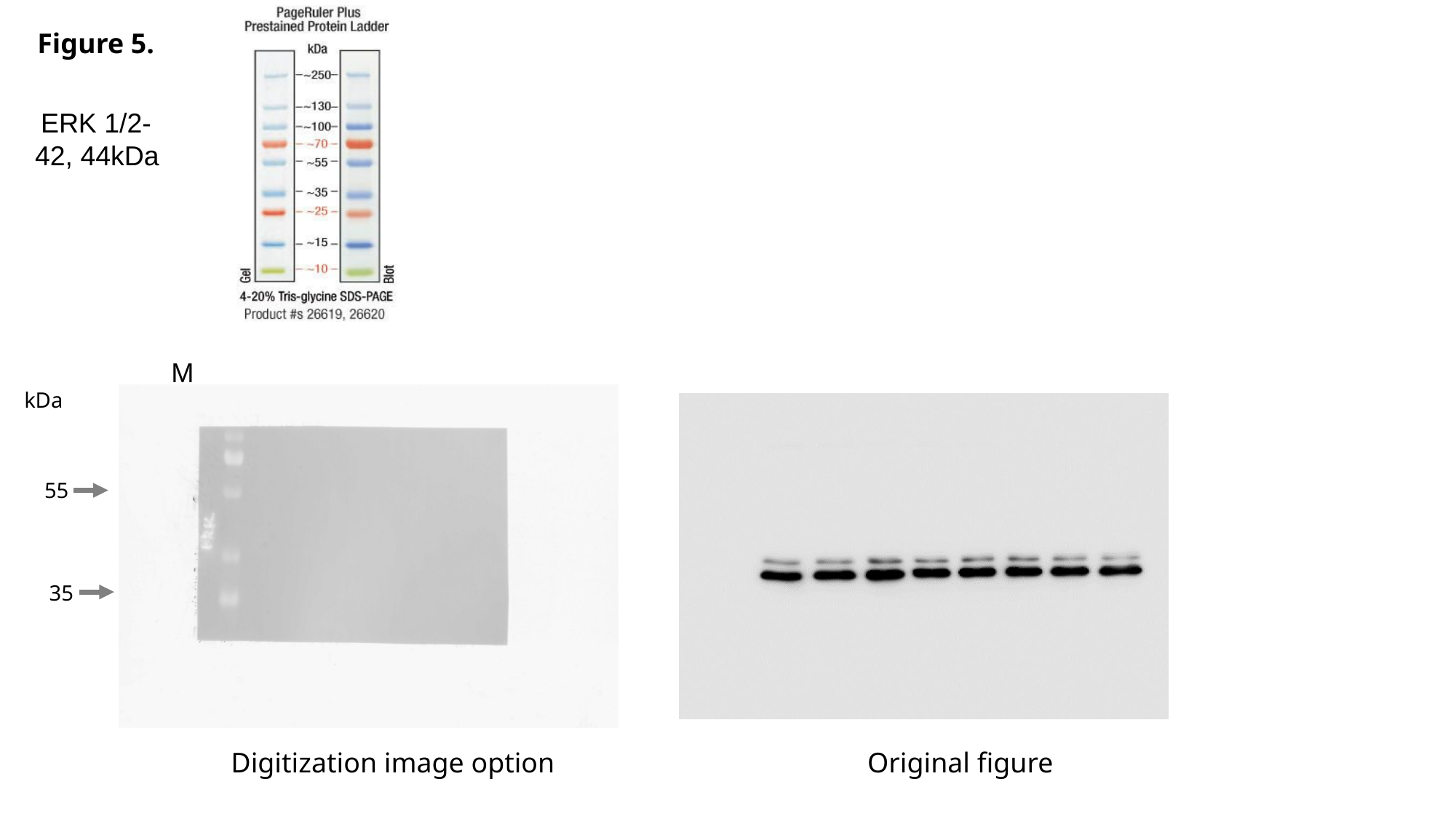

Figure 5.
ERK 1/2-
42, 44kDa
M
kDa
55
35
Digitization image option
Original figure

## Slide 54
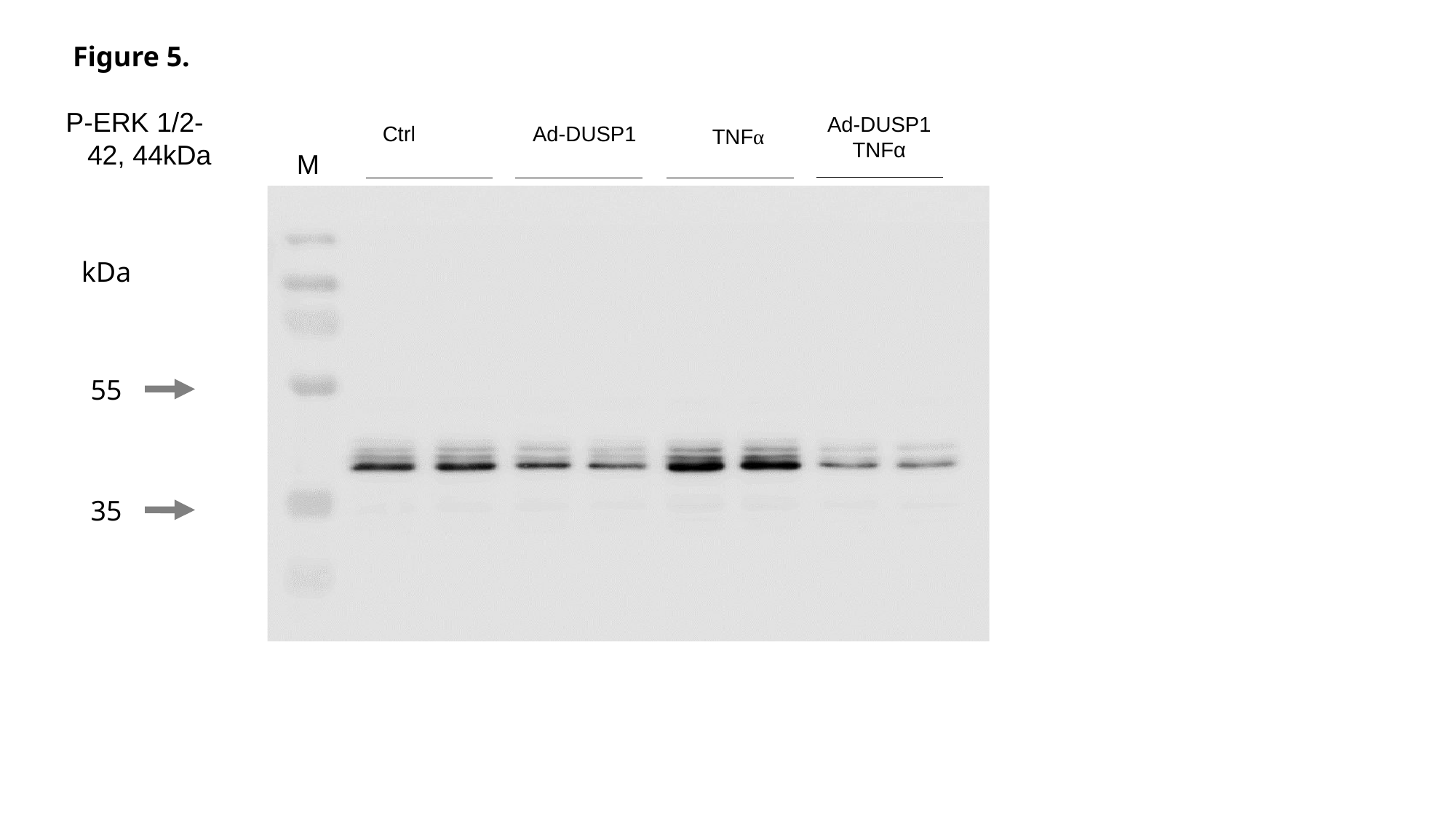

Figure 5.
P-ERK 1/2-
42, 44kDa
Ad-DUSP1
TNFα
Ctrl
Ad-DUSP1
TNFα
M
kDa
55
35

## Slide 55
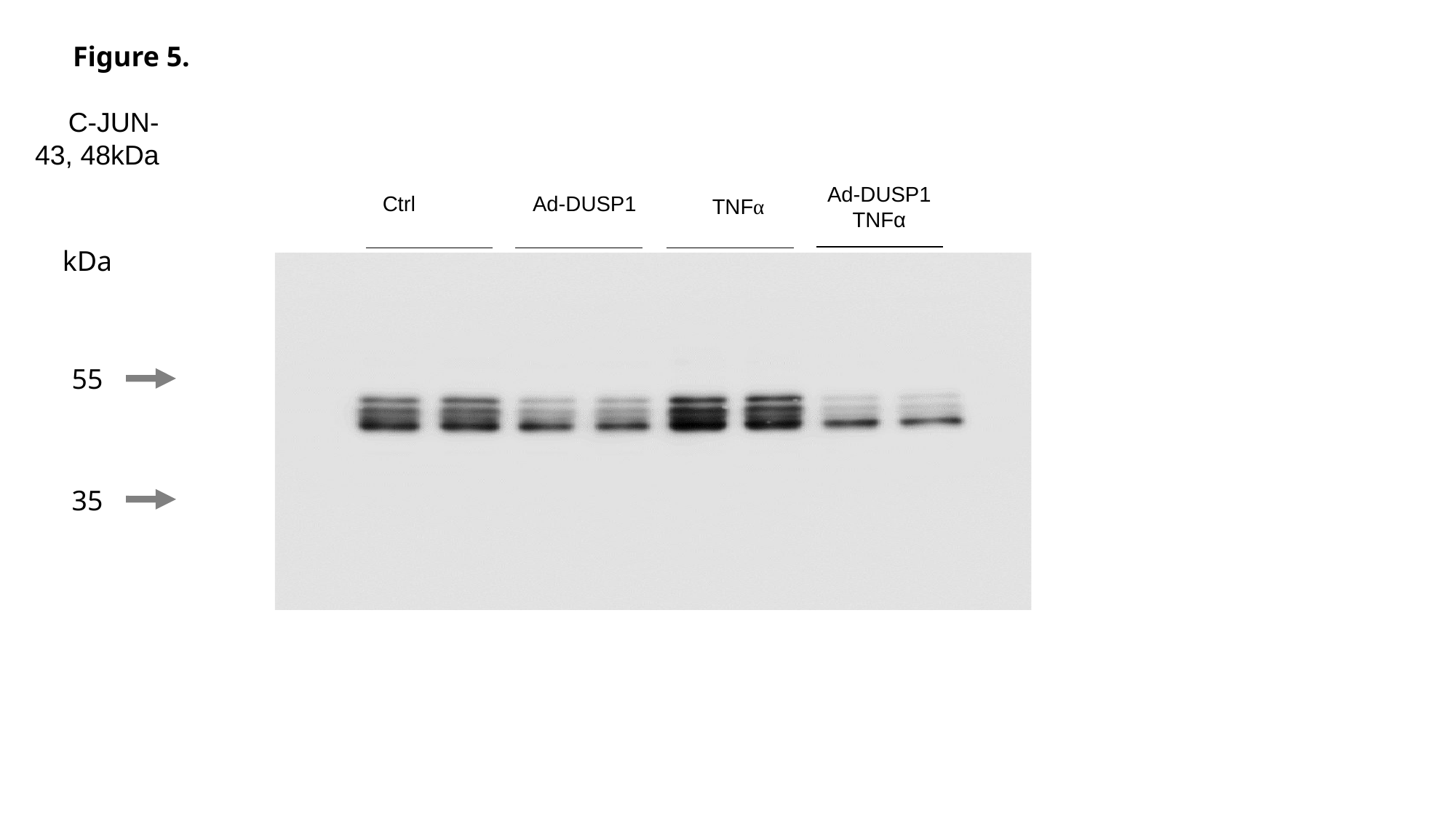

Figure 5.
C-JUN-
43, 48kDa
Ad-DUSP1
TNFα
Ctrl
Ad-DUSP1
TNFα
kDa
55
35

## Slide 56
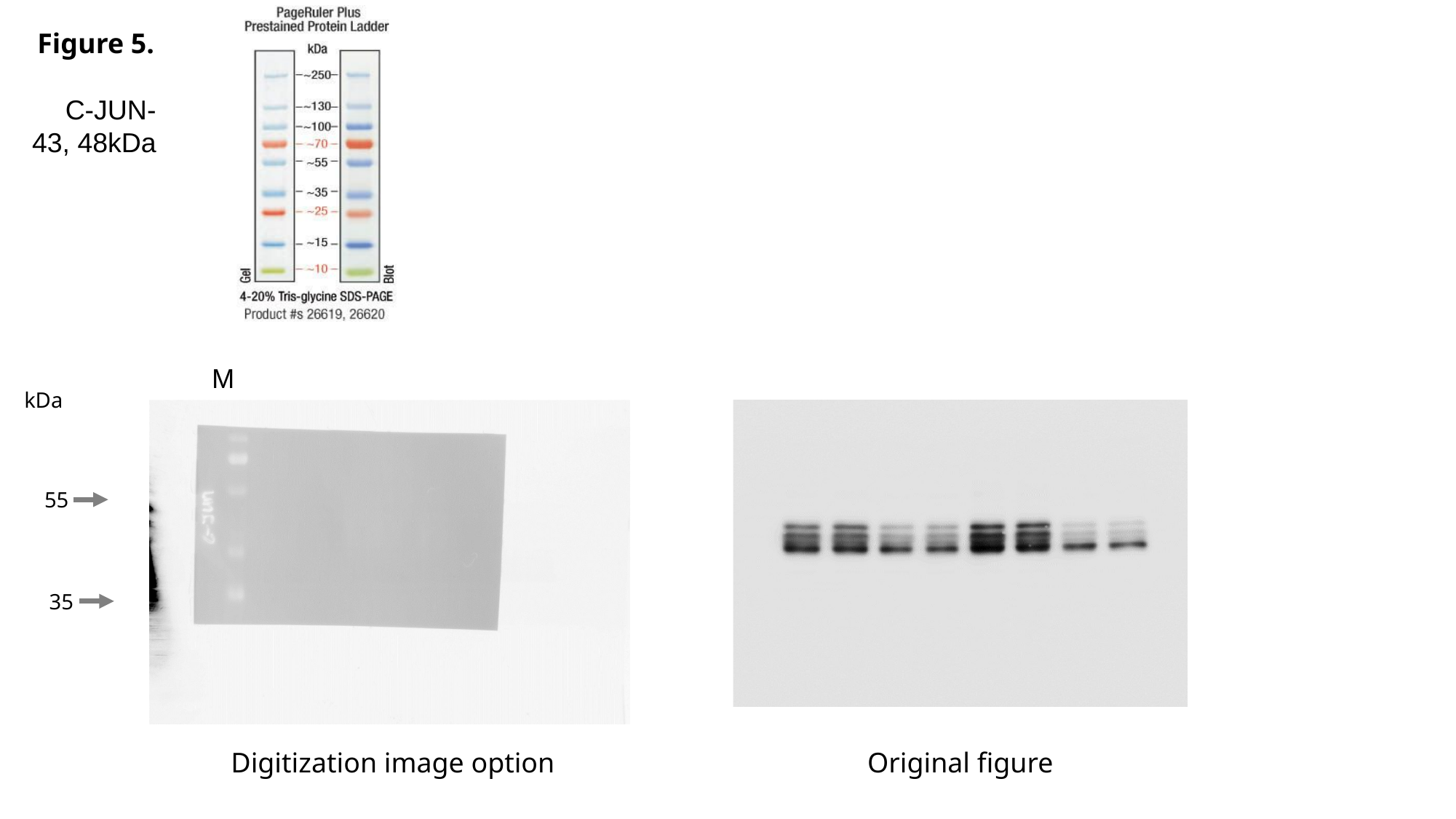

Figure 5.
C-JUN-
43, 48kDa
M
kDa
55
35
Digitization image option
Original figure

## Slide 57
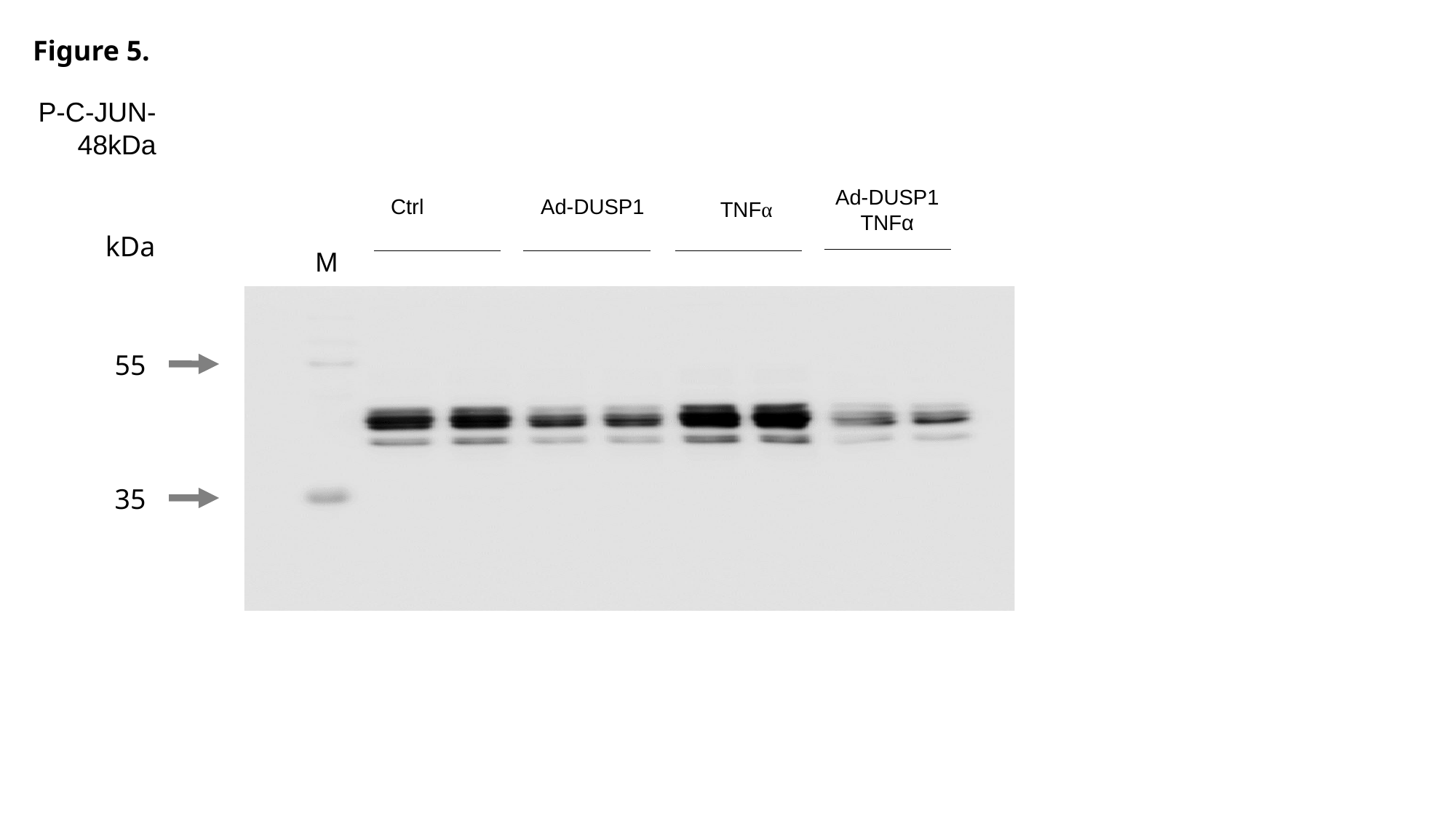

Figure 5.
P-C-JUN- 48kDa
Ad-DUSP1
TNFα
Ctrl
Ad-DUSP1
TNFα
kDa
M
55
35

## Slide 58
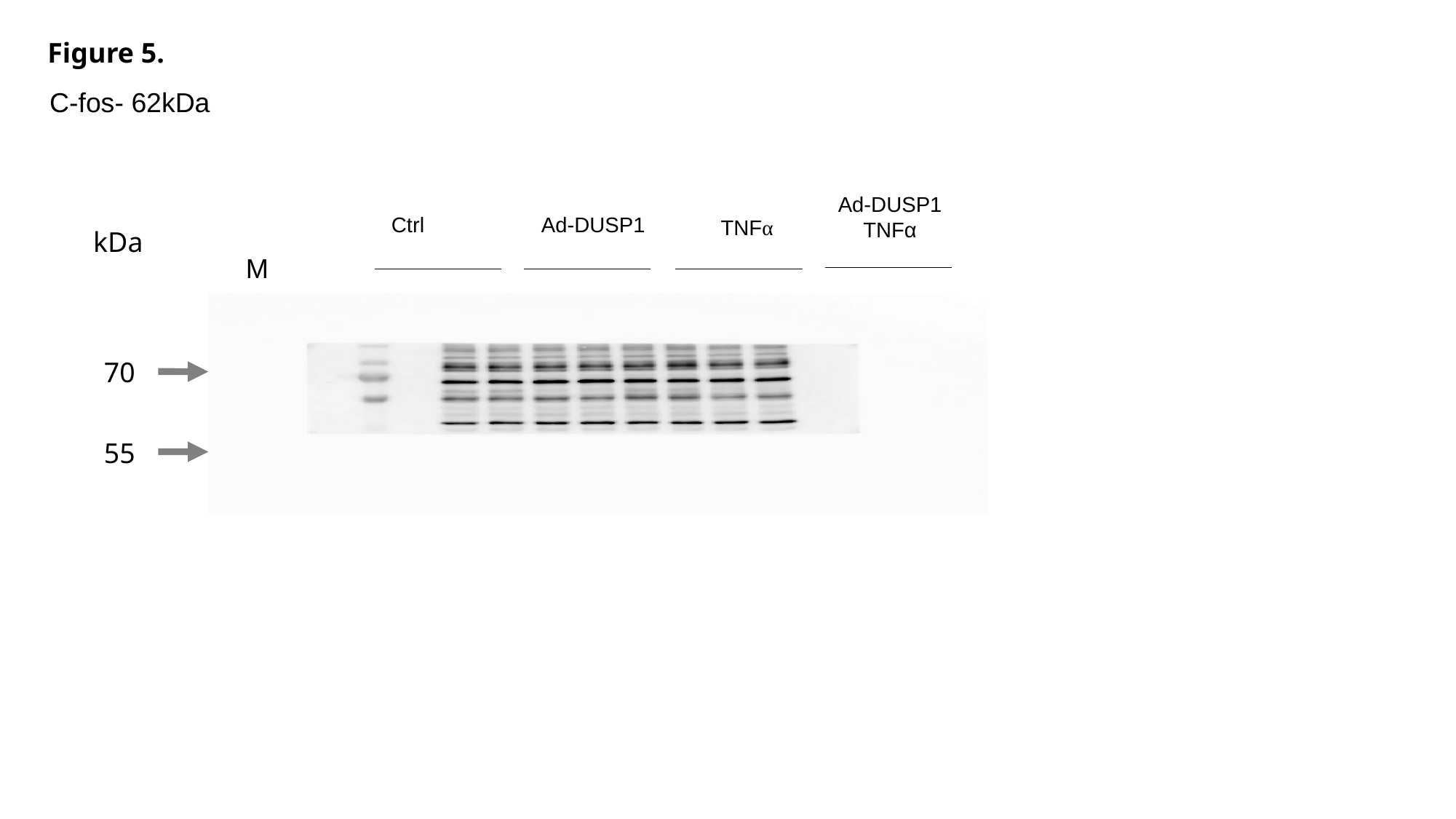

Figure 5.
C-fos- 62kDa
Ad-DUSP1
TNFα
Ctrl
Ad-DUSP1
TNFα
kDa
M
70
55

## Slide 59
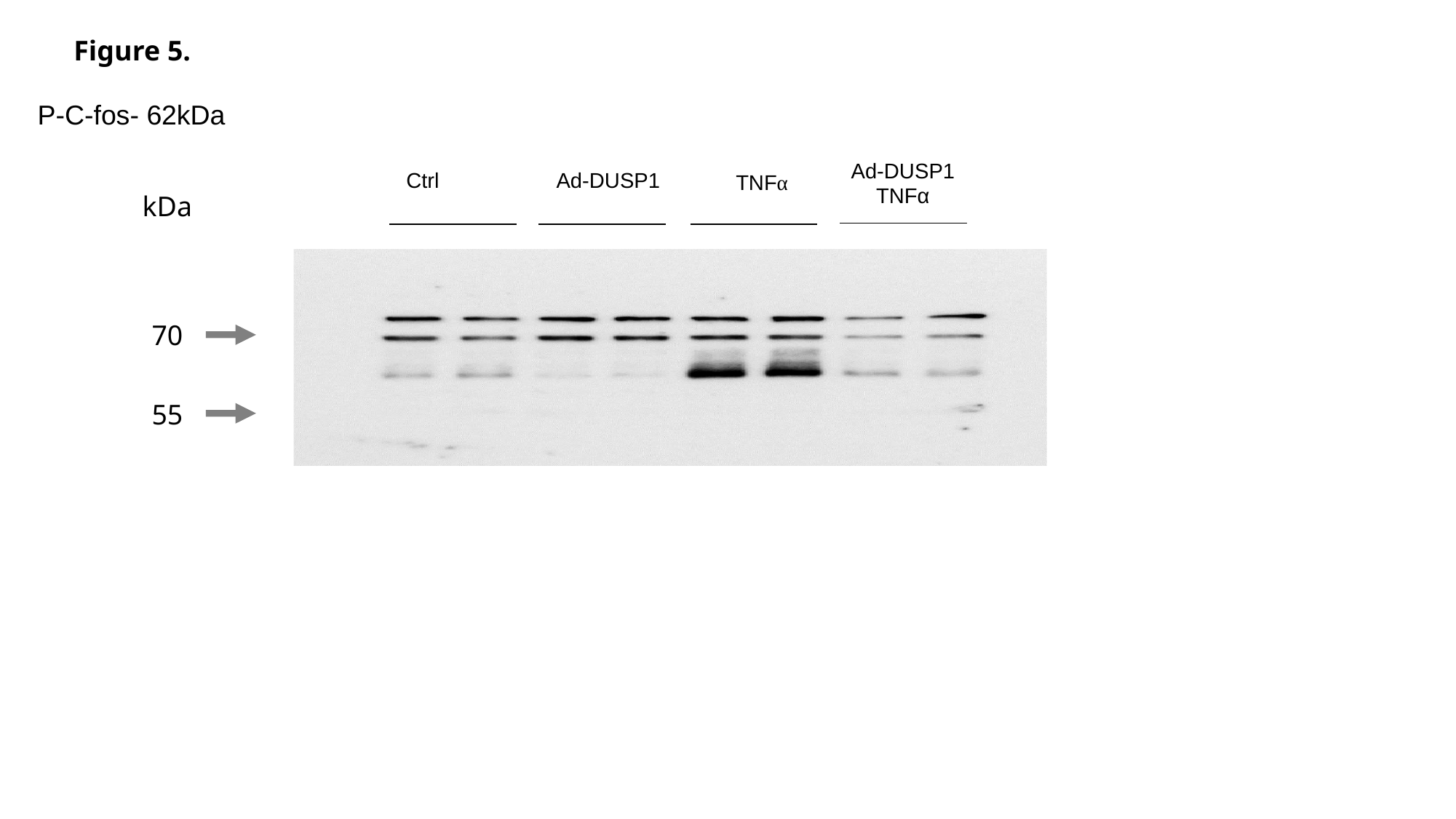

Figure 5.
P-C-fos- 62kDa
Ad-DUSP1
TNFα
Ctrl
Ad-DUSP1
TNFα
kDa
70
55

## Slide 60
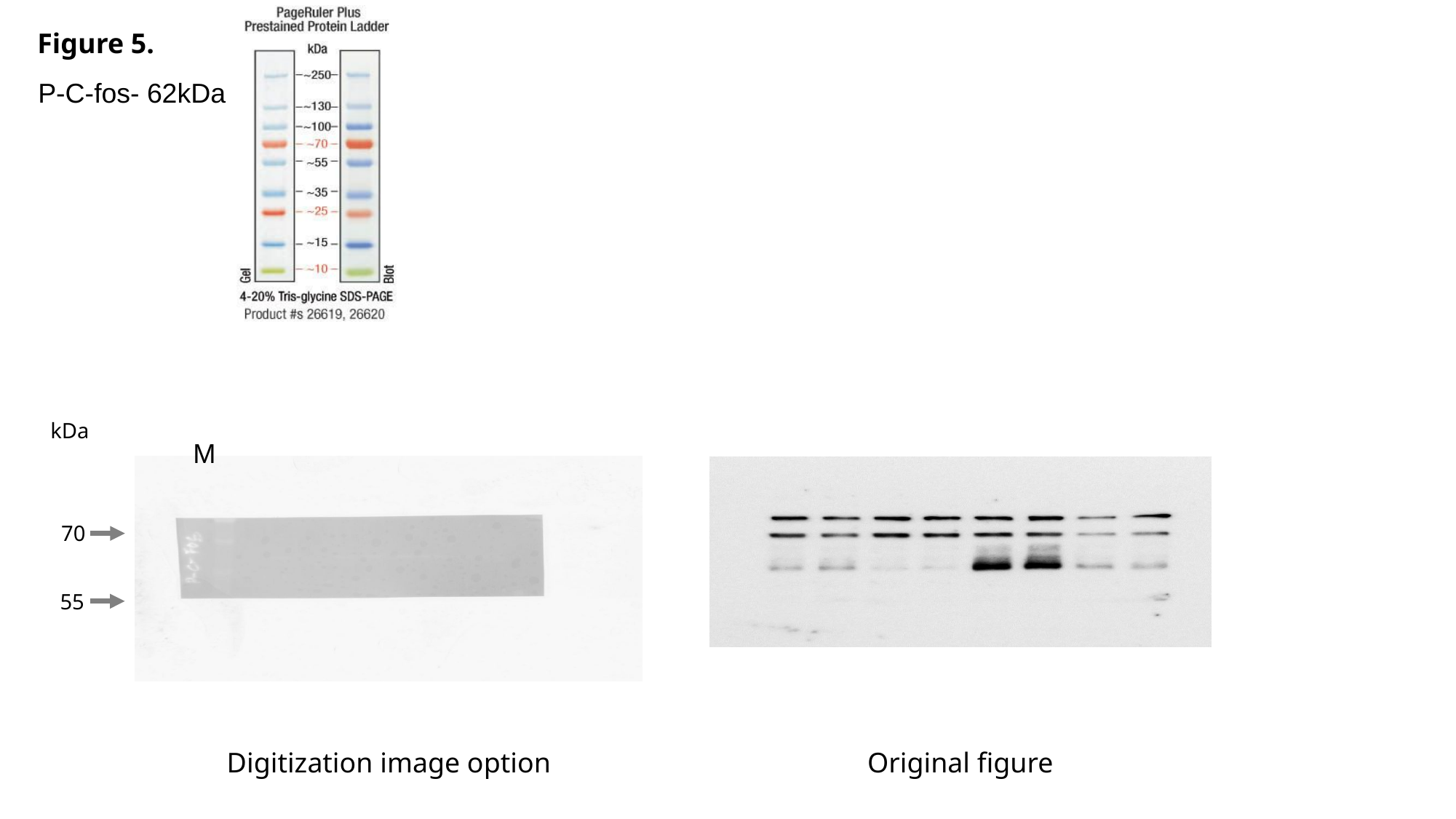

Figure 5.
P-C-fos- 62kDa
kDa
M
70
55
Digitization image option
Original figure

## Slide 61
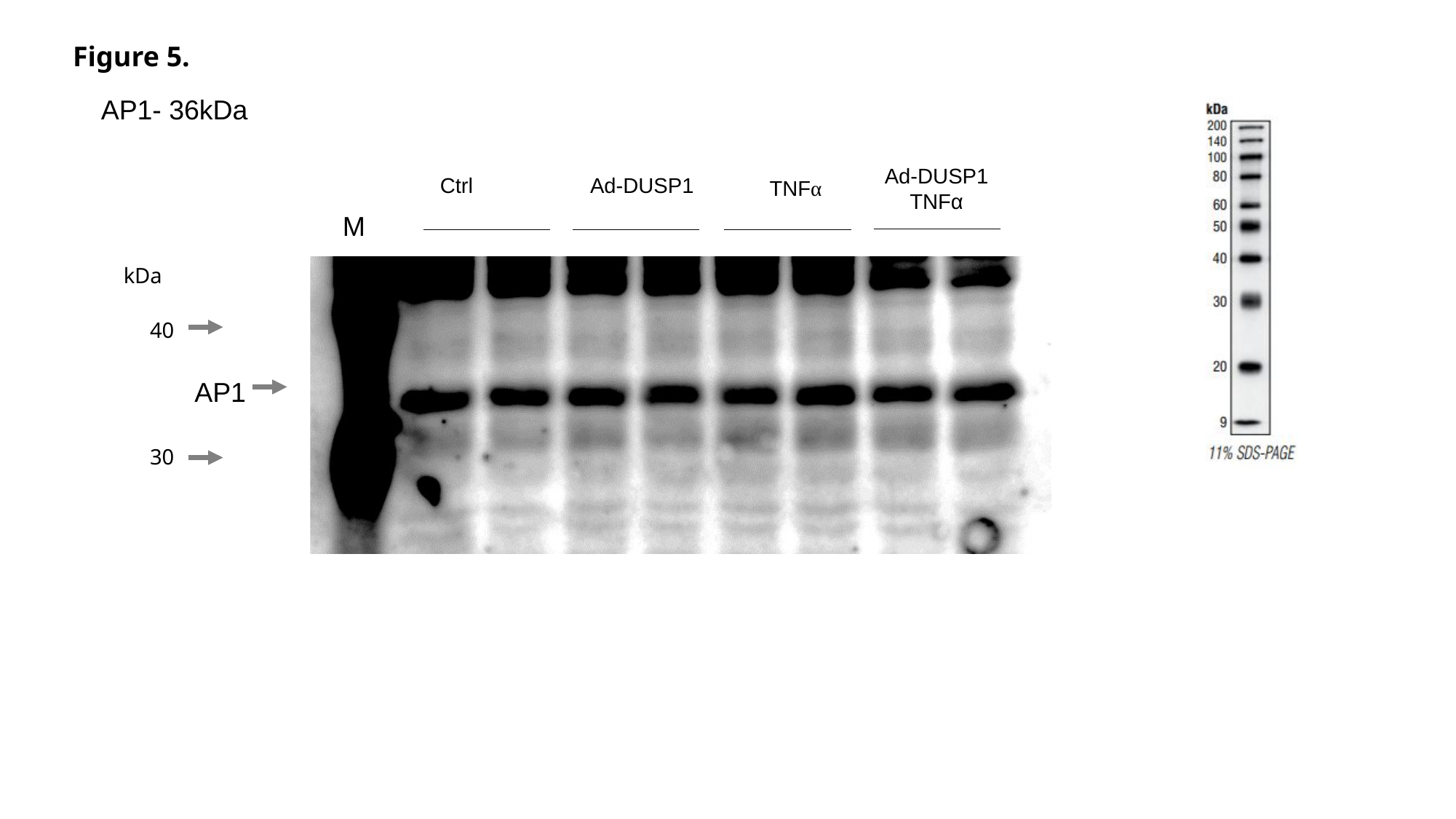

Figure 5.
AP1- 36kDa
Ad-DUSP1
TNFα
Ctrl
Ad-DUSP1
TNFα
M
kDa
40
AP1
30

## Slide 62
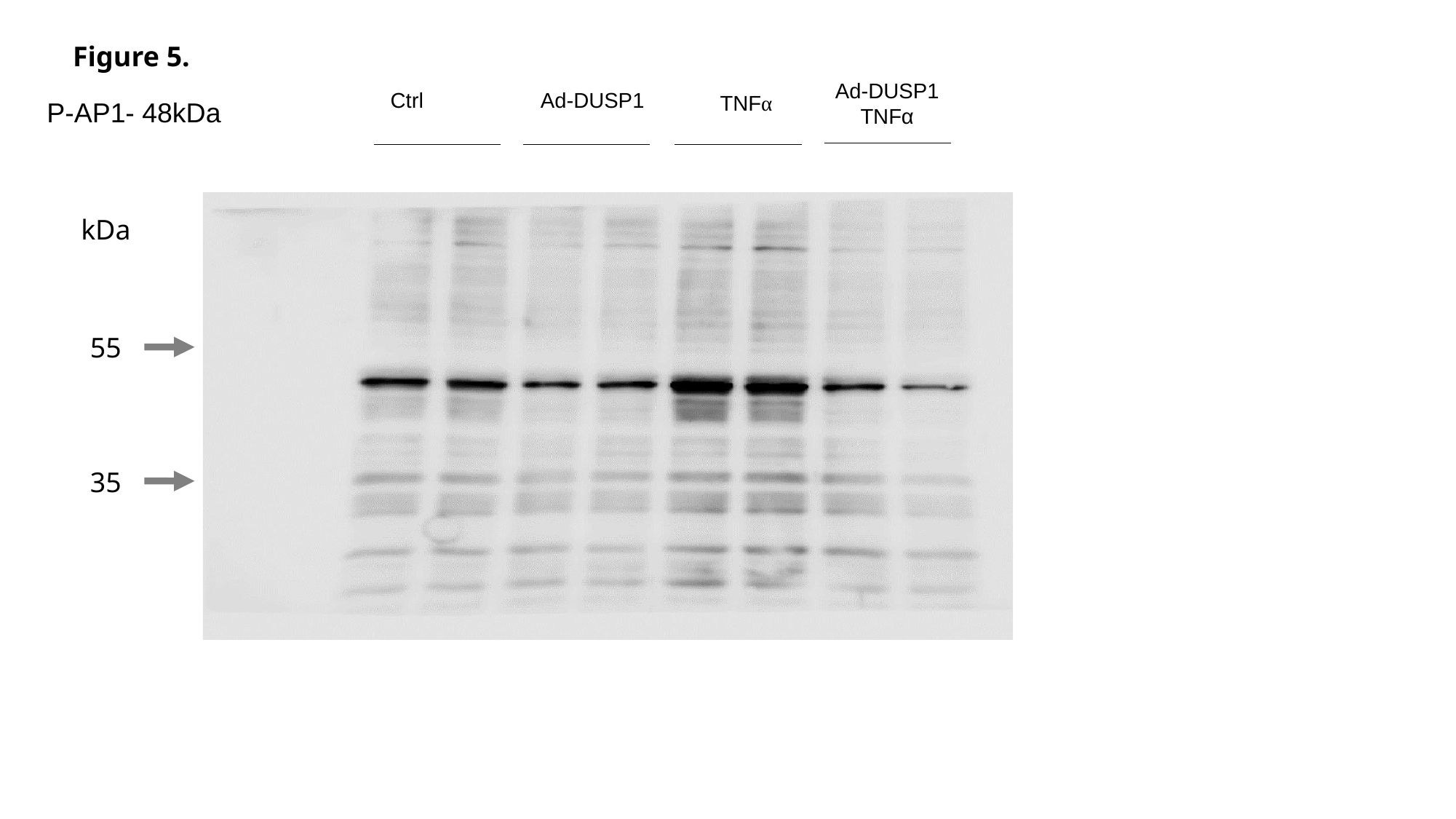

Figure 5.
Ad-DUSP1
TNFα
Ctrl
Ad-DUSP1
TNFα
P-AP1- 48kDa
kDa
55
35

## Slide 63
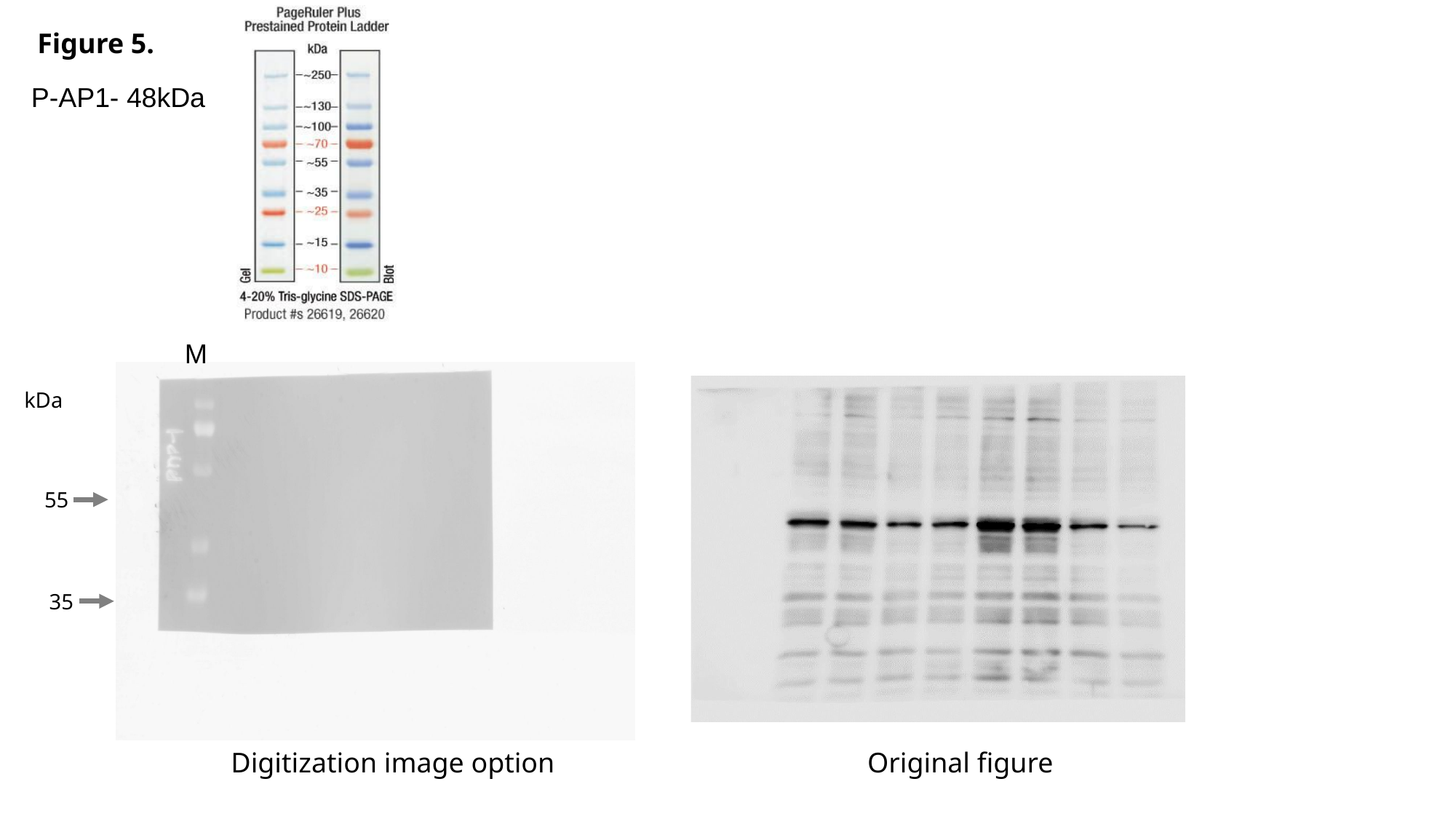

Figure 5.
P-AP1- 48kDa
M
kDa
55
35
Digitization image option
Original figure

## Slide 64
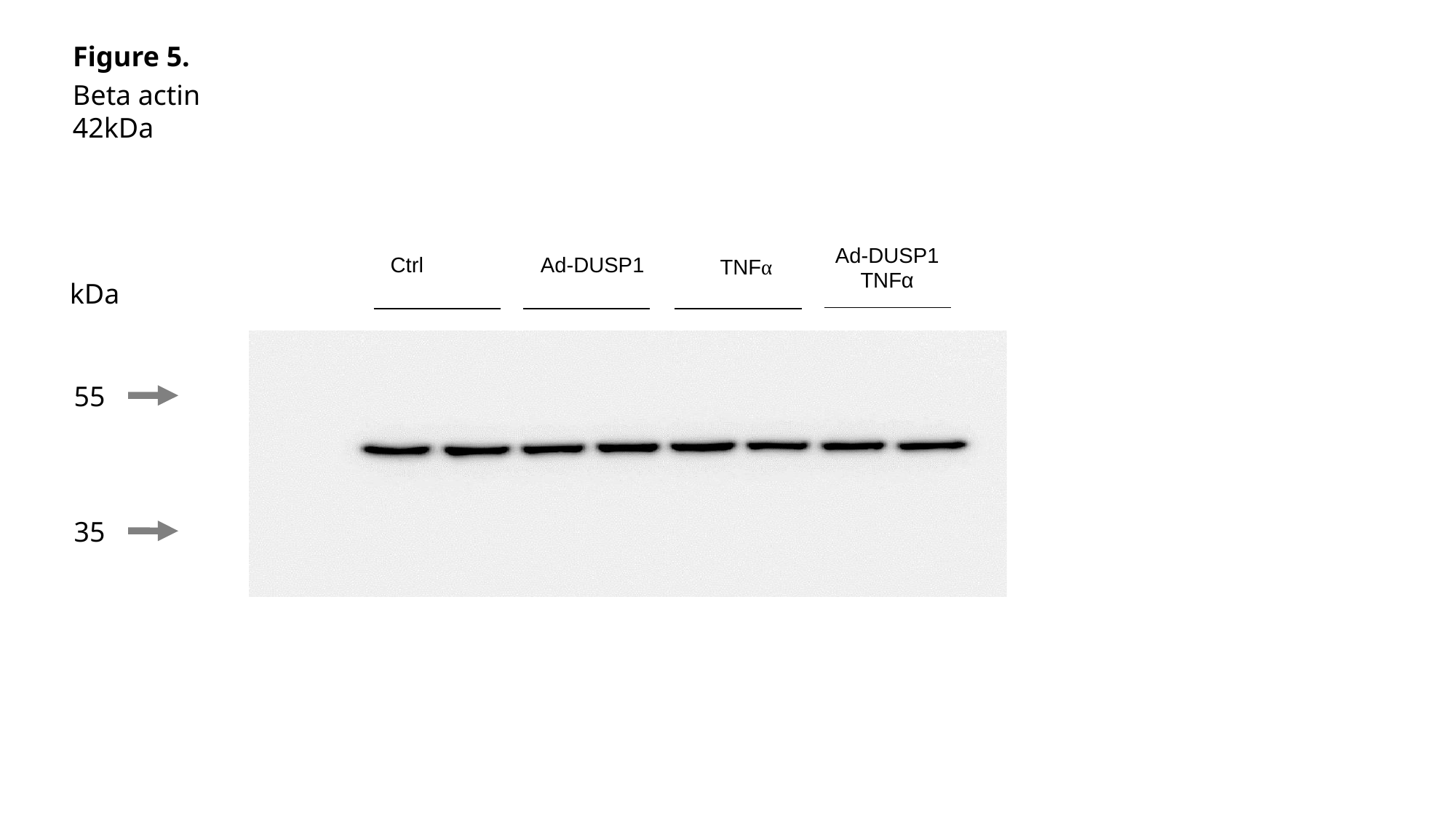

Figure 5.
Beta actin
42kDa
Ad-DUSP1
TNFα
Ctrl
Ad-DUSP1
TNFα
kDa
55
35

## Slide 65
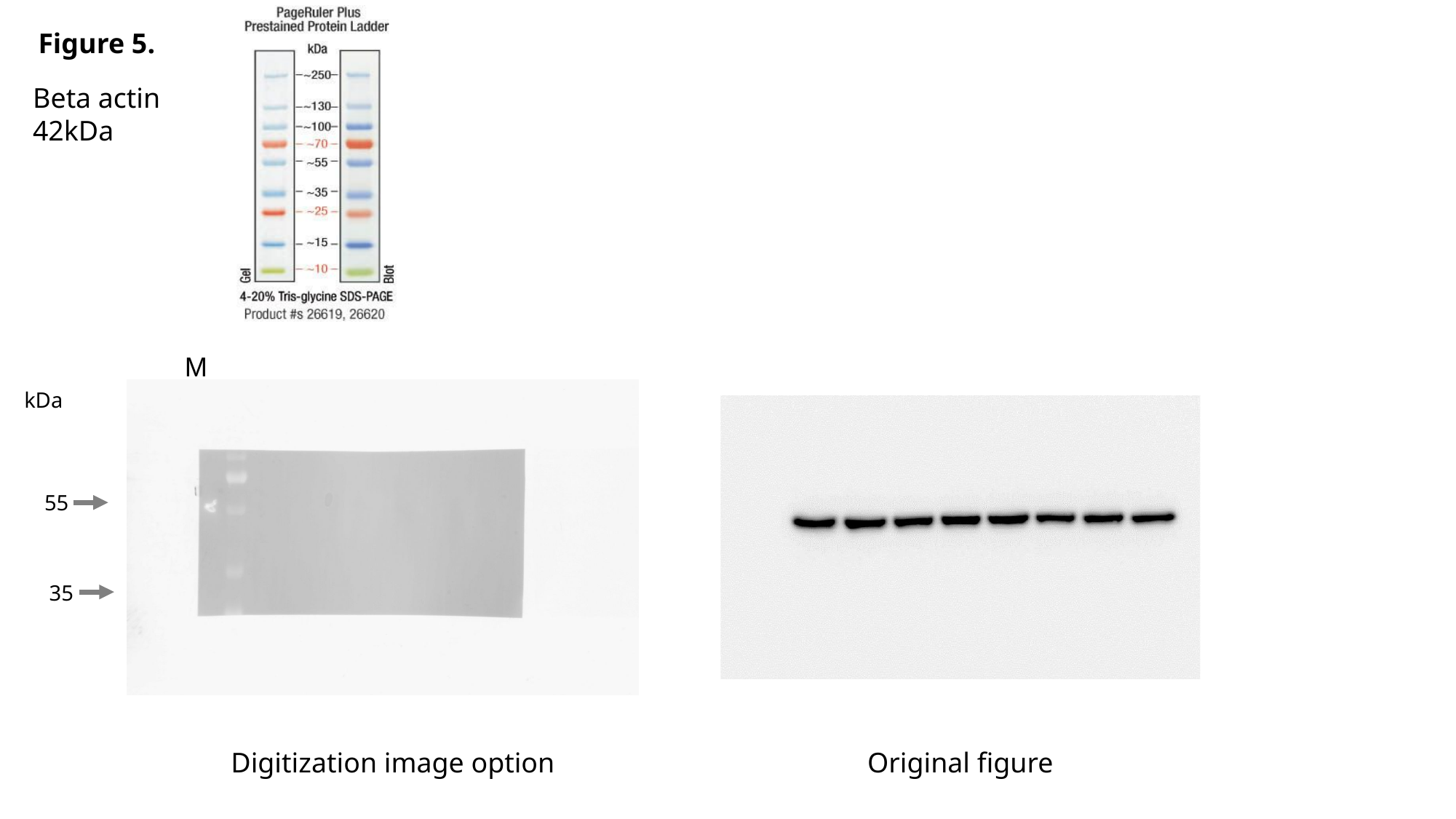

Figure 5.
Beta actin
42kDa
M
kDa
55
35
Digitization image option
Original figure

## Slide 66
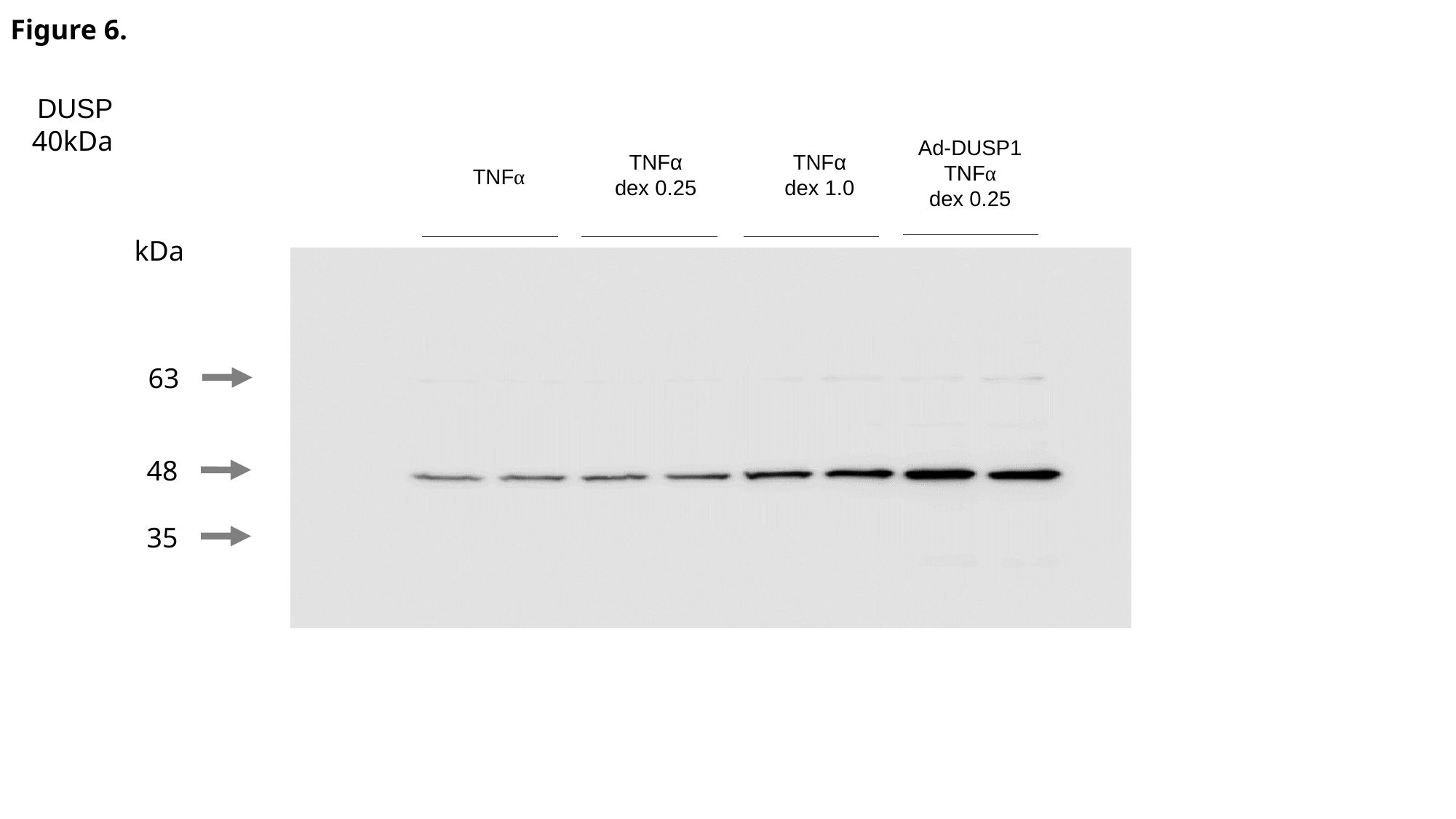

Figure 6.
DUSP
40kDa
Ad-DUSP1
TNFα
dex 0.25
TNFα
dex 0.25
TNFα
dex 1.0
TNFα
kDa
63
48
35

## Slide 67
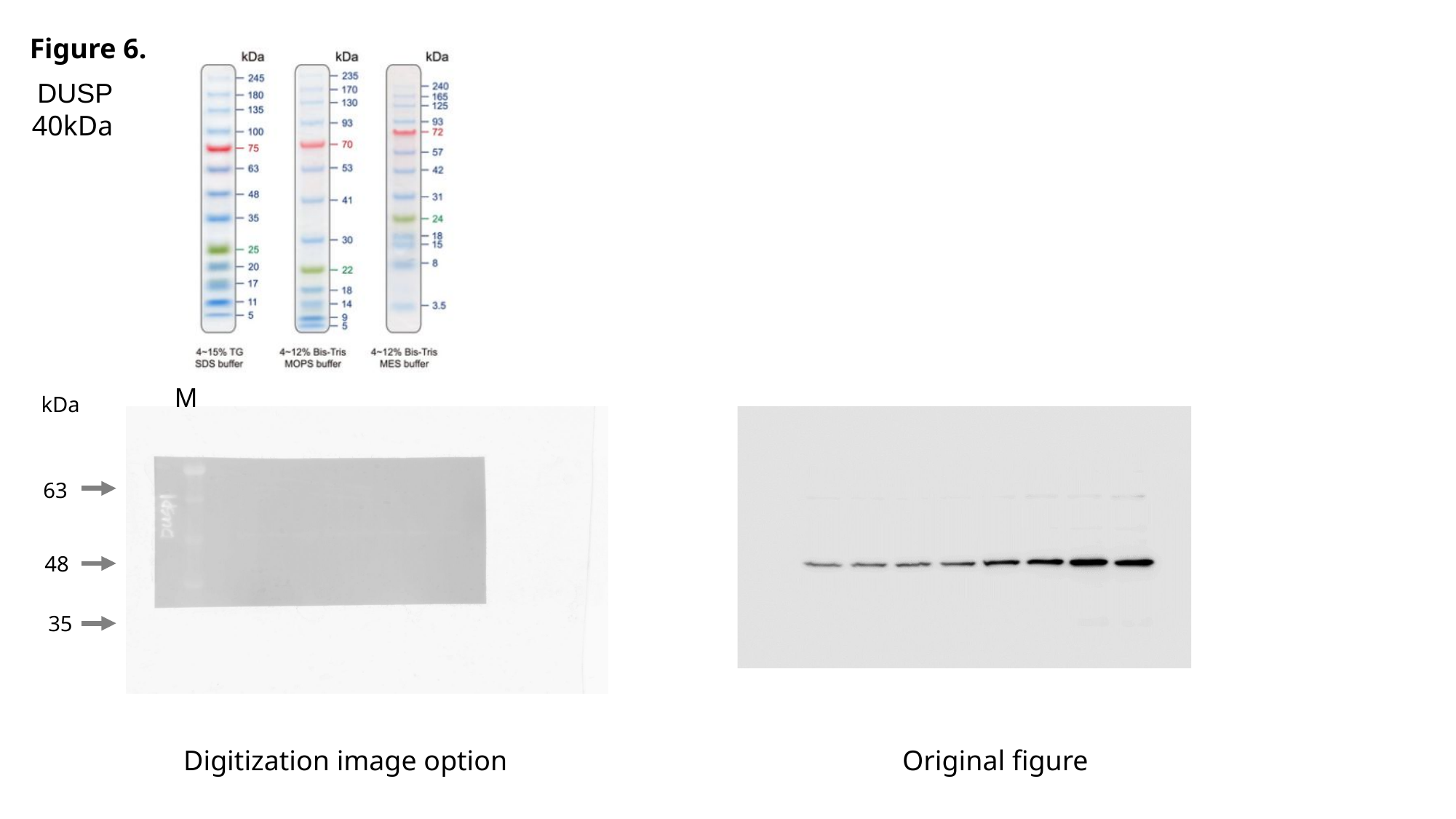

Figure 6.
DUSP
40kDa
M
kDa
63
48
35
Digitization image option
Original figure

## Slide 68
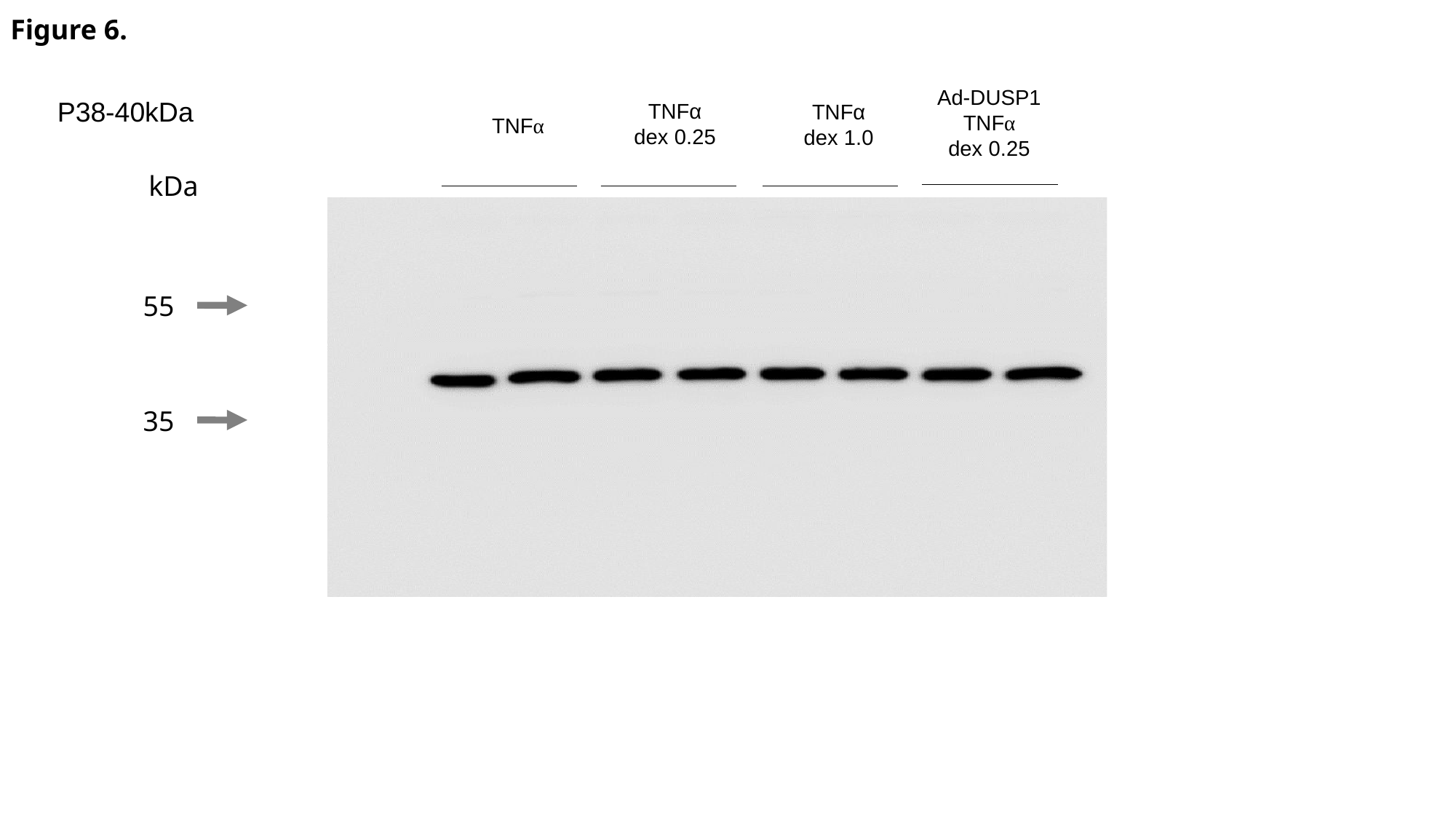

Figure 6.
Ad-DUSP1
TNFα
dex 0.25
P38-40kDa
TNFα
dex 0.25
TNFα
dex 1.0
TNFα
kDa
55
35

## Slide 69
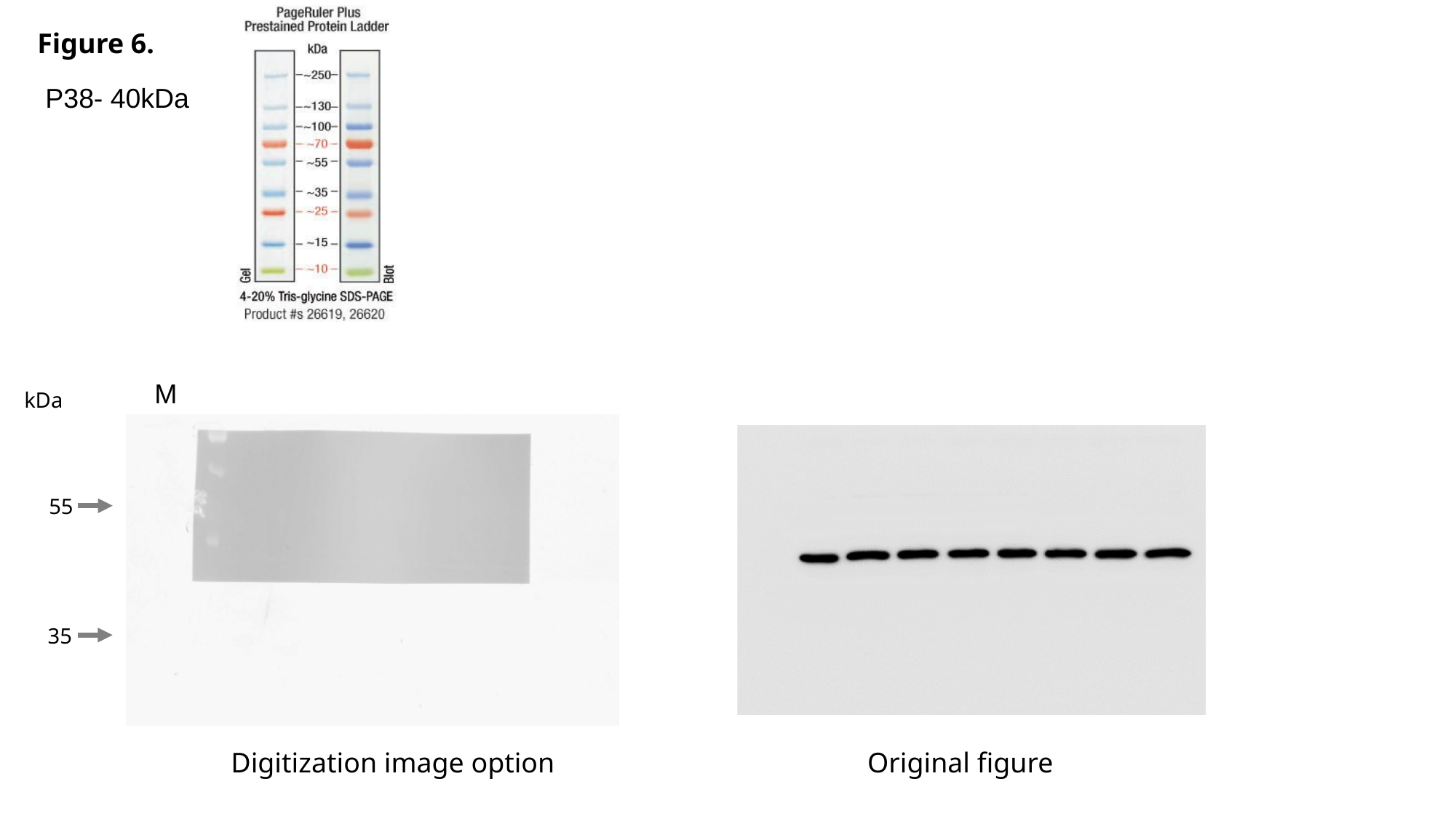

Figure 6.
P38- 40kDa
M
kDa
55
35
Digitization image option
Original figure

## Slide 70
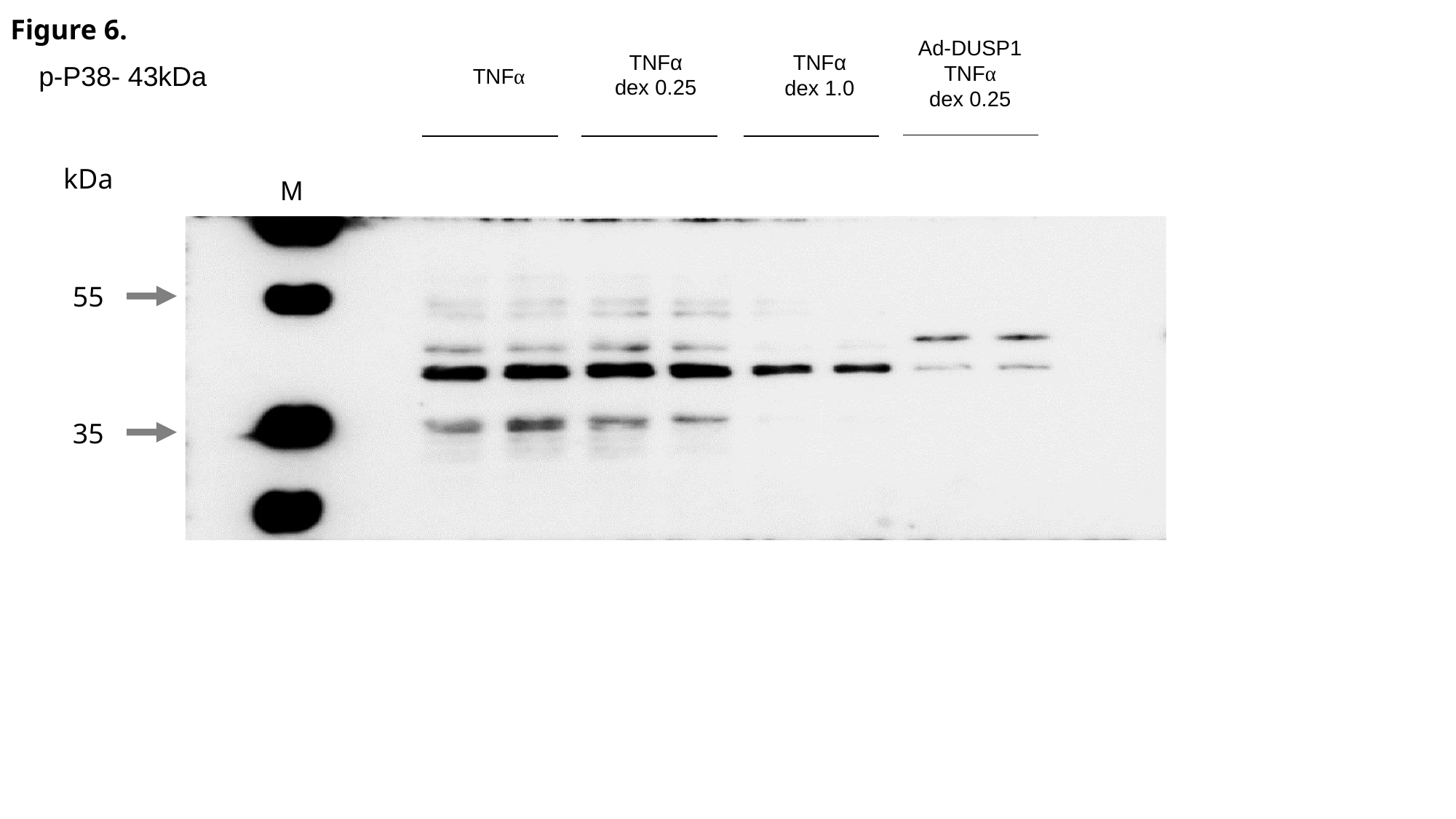

Figure 6.
Ad-DUSP1
TNFα
dex 0.25
TNFα
dex 0.25
TNFα
dex 1.0
p-P38- 43kDa
TNFα
kDa
M
55
35

## Slide 71
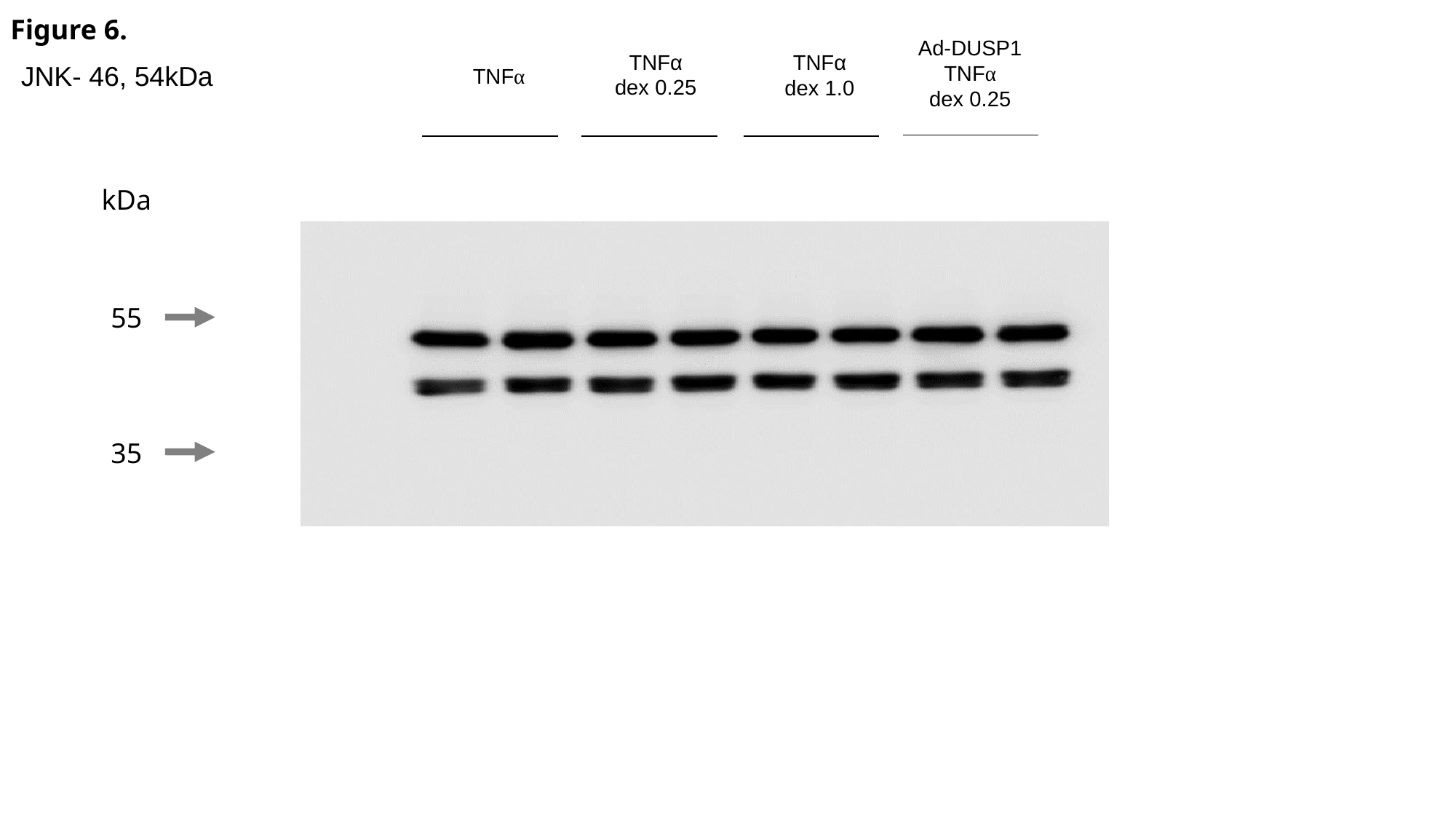

Figure 6.
Ad-DUSP1
TNFα
dex 0.25
TNFα
dex 0.25
TNFα
dex 1.0
JNK- 46, 54kDa
TNFα
kDa
55
35

## Slide 72
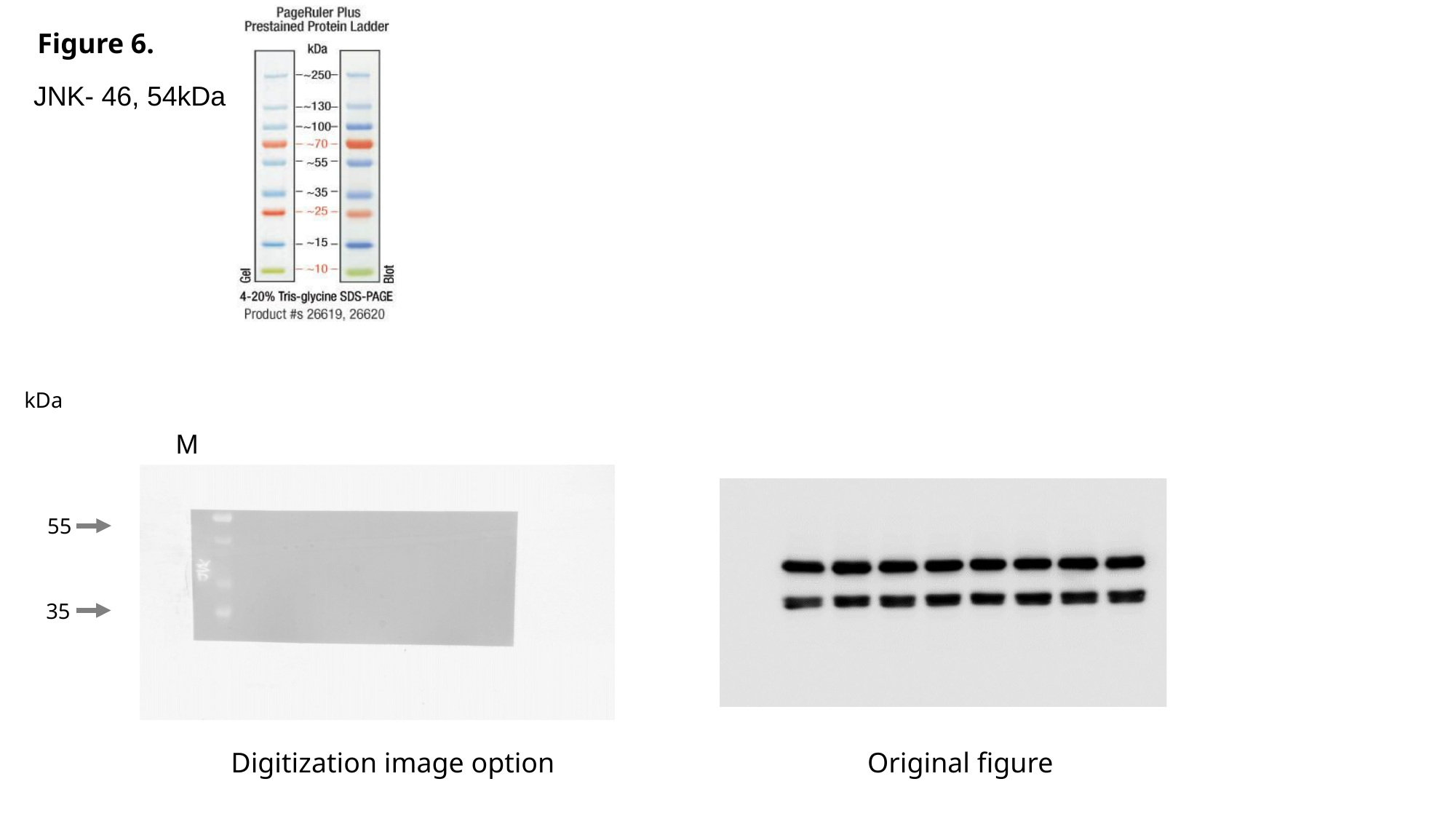

Figure 6.
JNK- 46, 54kDa
kDa
M
55
35
Digitization image option
Original figure

## Slide 73
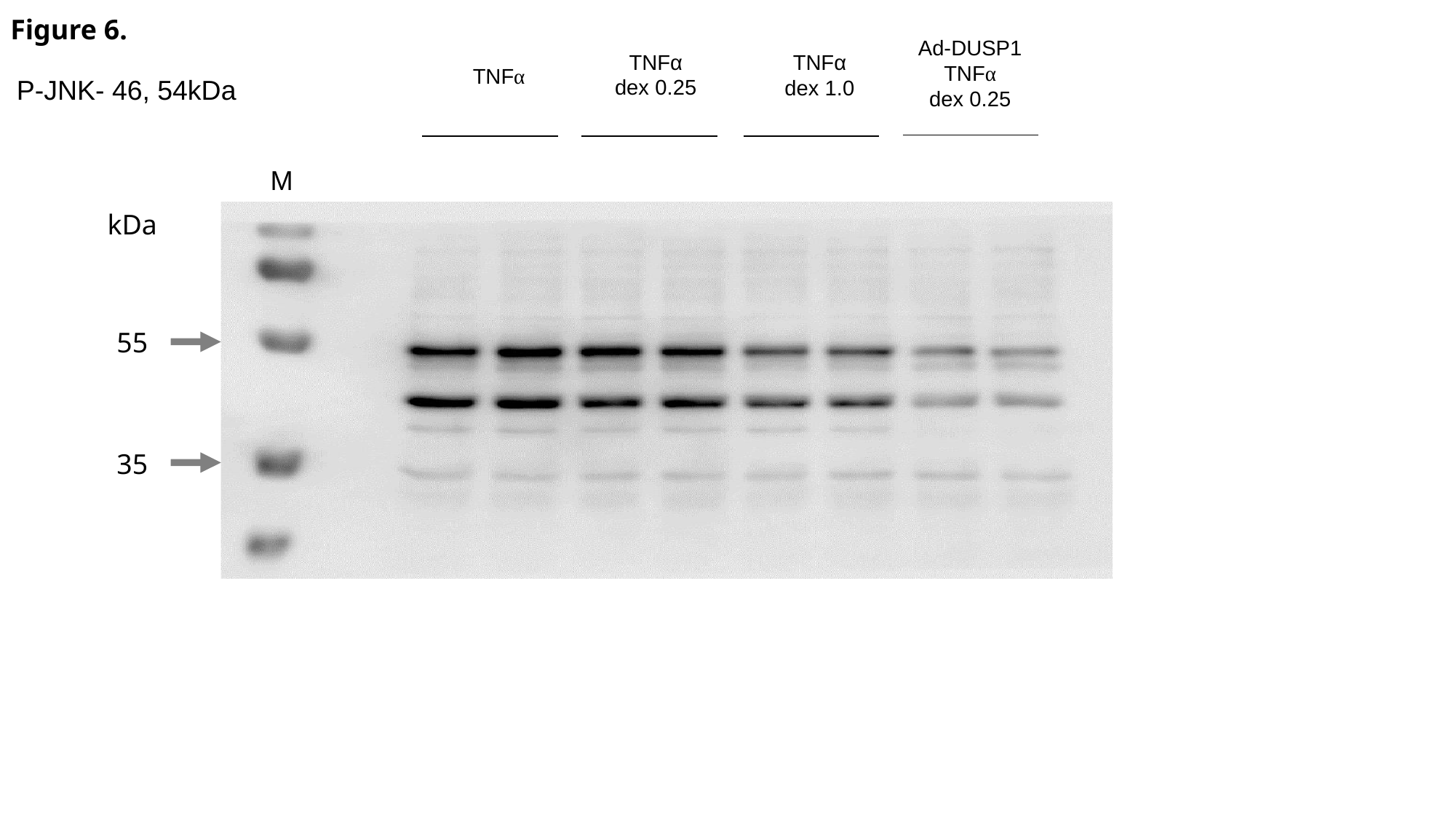

Figure 6.
Ad-DUSP1
TNFα
dex 0.25
TNFα
dex 0.25
TNFα
dex 1.0
TNFα
P-JNK- 46, 54kDa
M
kDa
55
35

## Slide 74
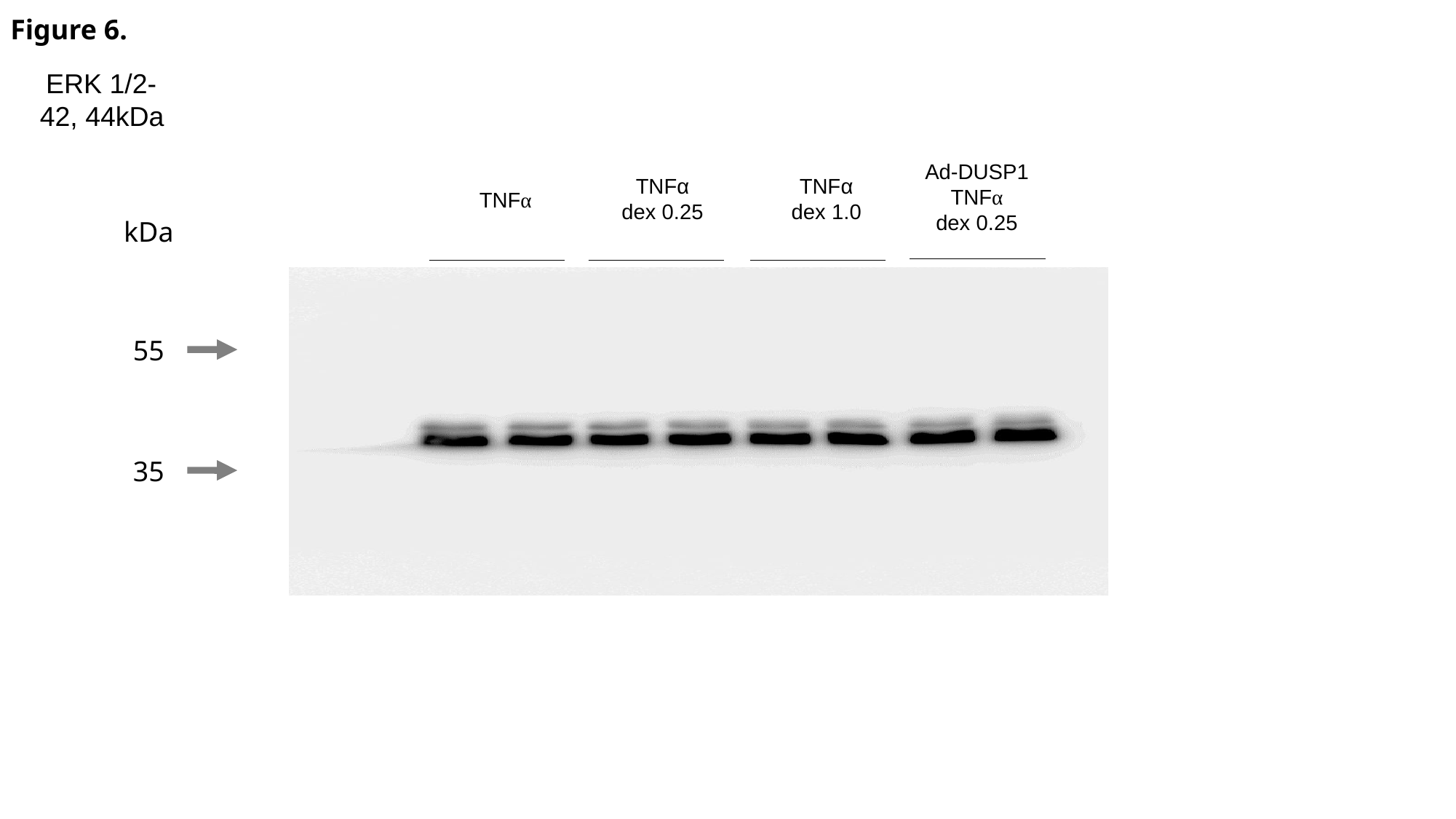

Figure 6.
ERK 1/2-
42, 44kDa
Ad-DUSP1
TNFα
dex 0.25
TNFα
dex 0.25
TNFα
dex 1.0
TNFα
kDa
55
35

## Slide 75
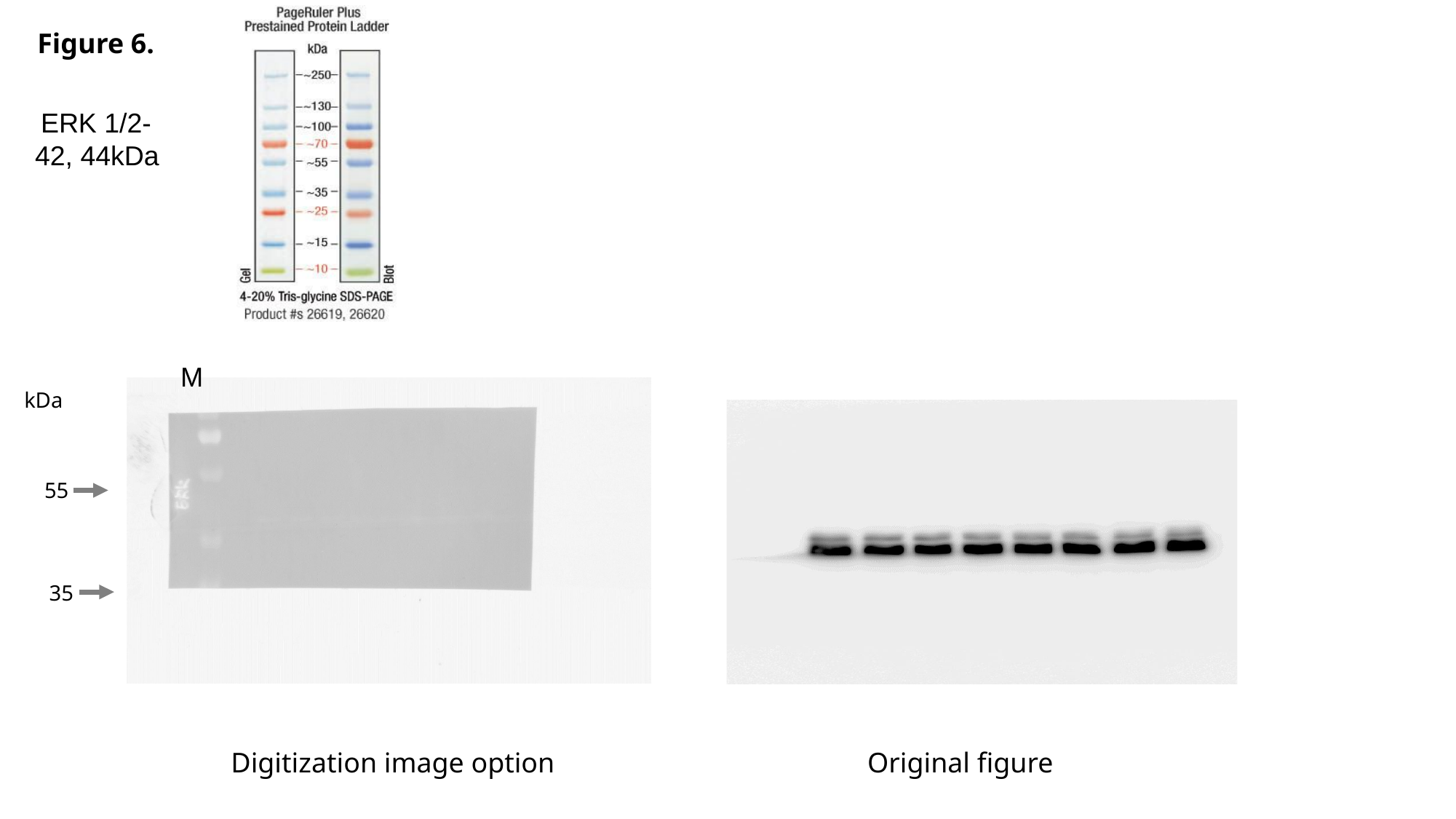

Figure 6.
ERK 1/2-
42, 44kDa
M
kDa
55
35
Digitization image option
Original figure

## Slide 76
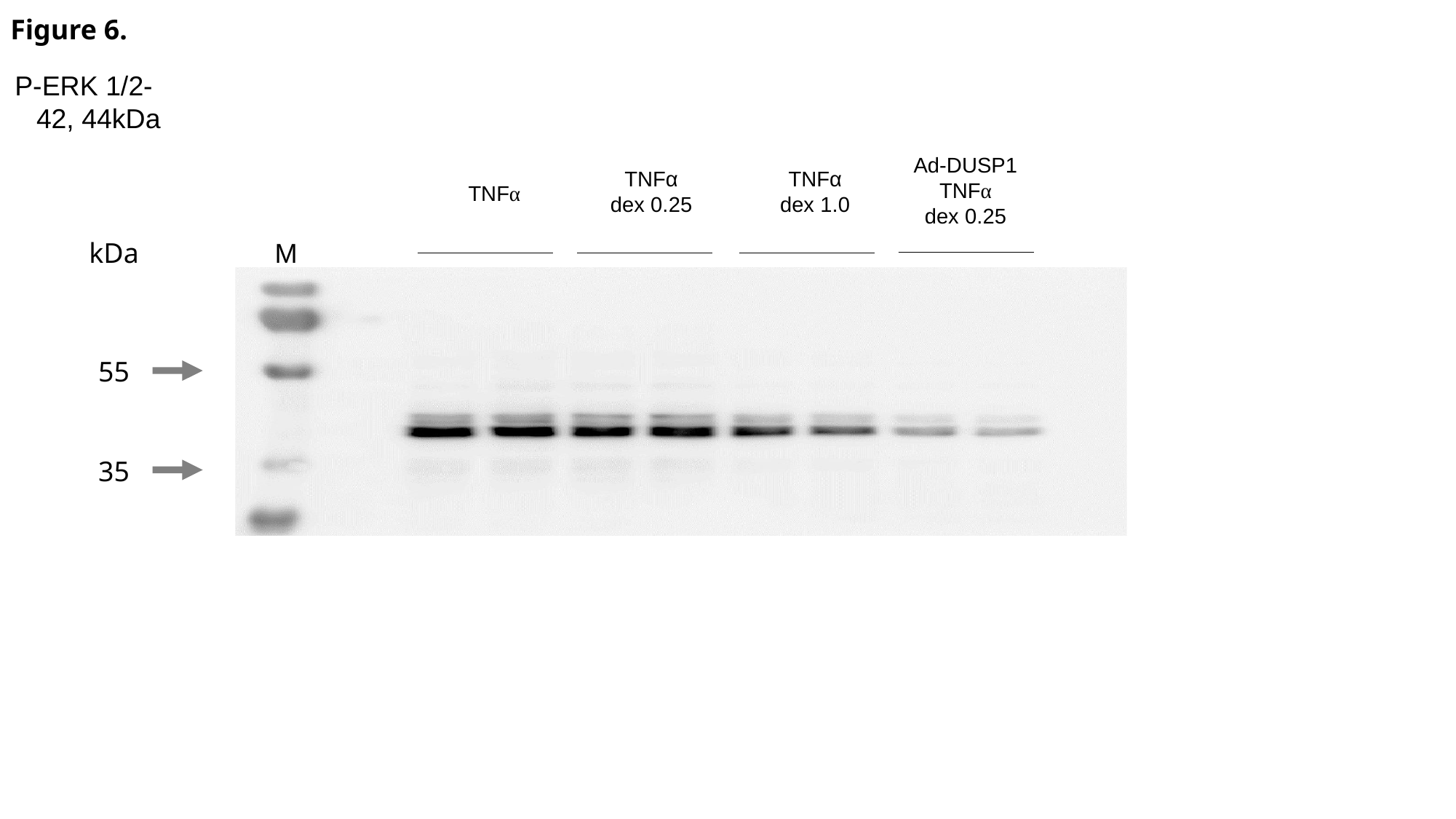

Figure 6.
P-ERK 1/2-
42, 44kDa
Ad-DUSP1
TNFα
dex 0.25
TNFα
dex 0.25
TNFα
dex 1.0
TNFα
kDa
M
55
35

## Slide 77
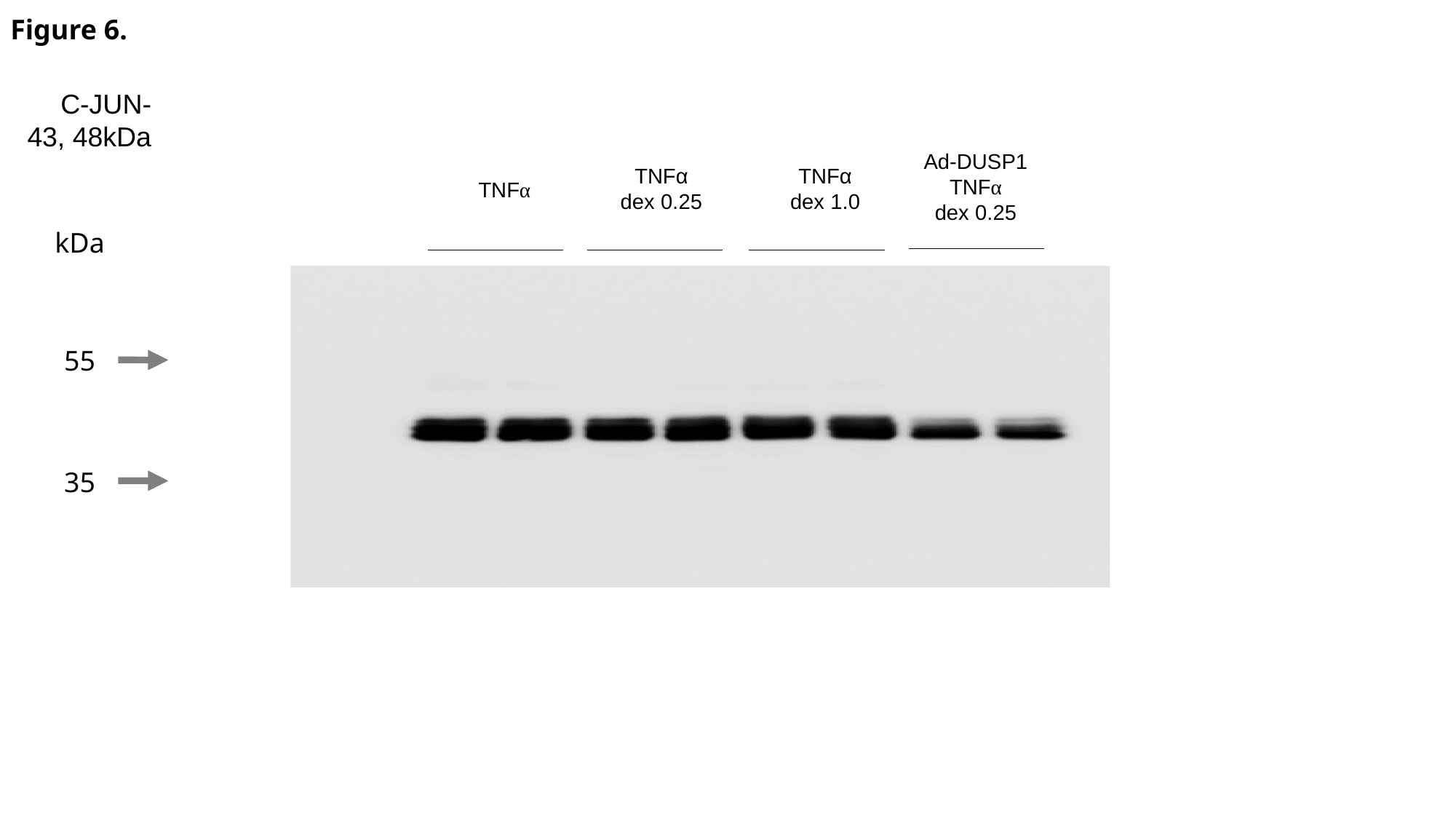

Figure 6.
C-JUN-
43, 48kDa
Ad-DUSP1
TNFα
dex 0.25
TNFα
dex 0.25
TNFα
dex 1.0
TNFα
kDa
55
35

## Slide 78
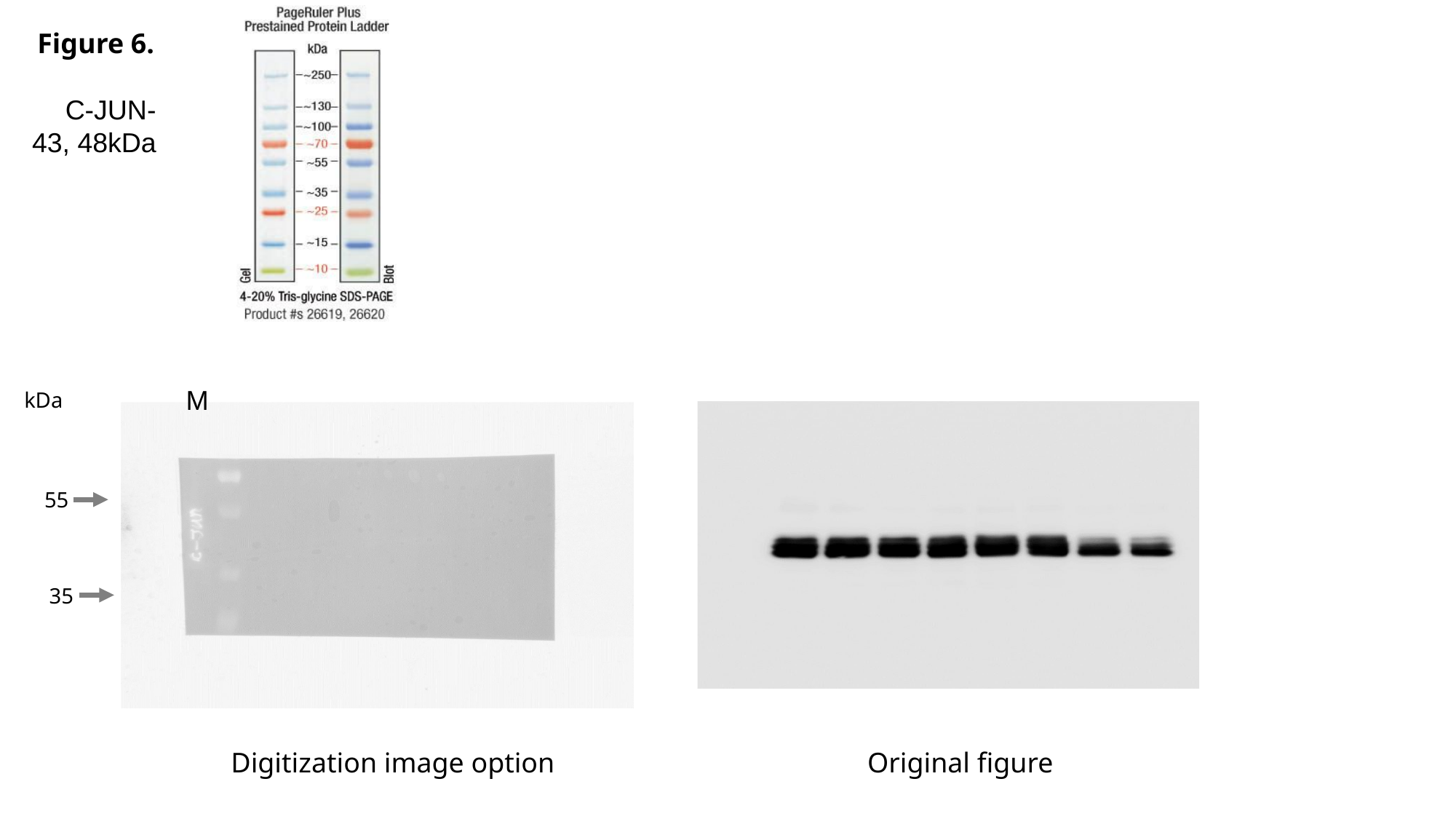

Figure 6.
C-JUN-
43, 48kDa
M
kDa
55
35
Digitization image option
Original figure

## Slide 79
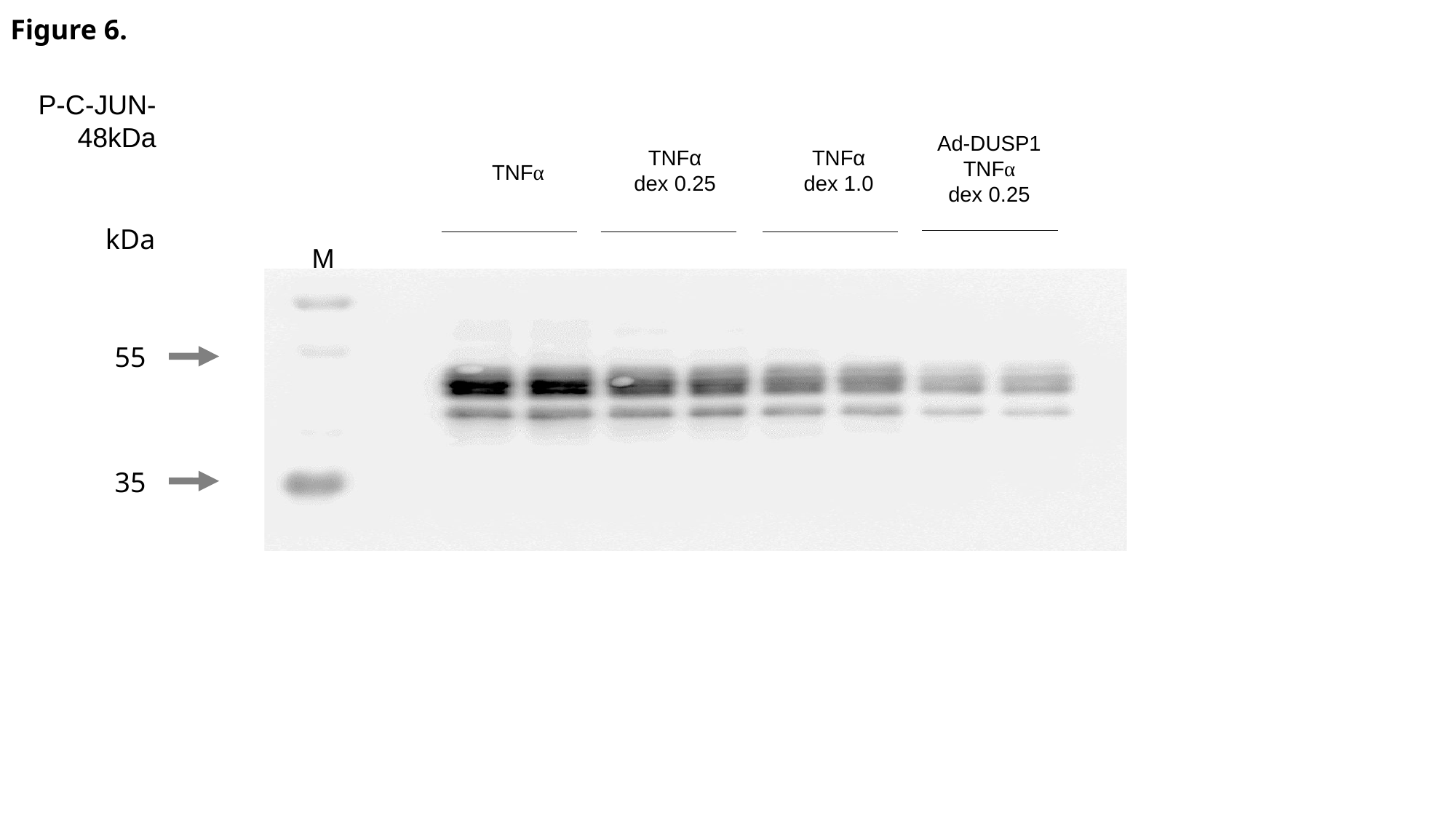

Figure 6.
P-C-JUN- 48kDa
Ad-DUSP1
TNFα
dex 0.25
TNFα
dex 0.25
TNFα
dex 1.0
TNFα
kDa
M
55
35

## Slide 80
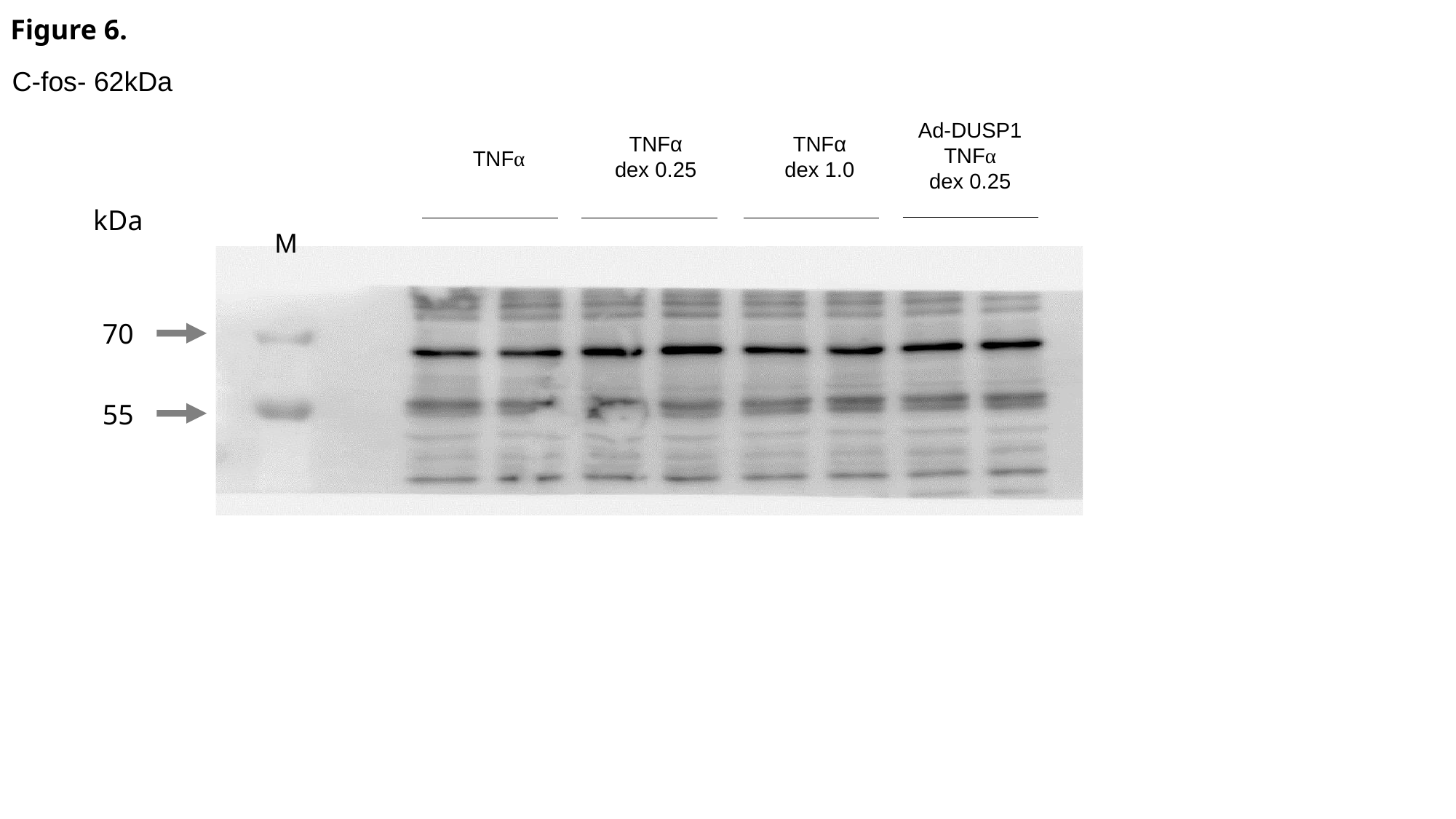

Figure 6.
C-fos- 62kDa
Ad-DUSP1
TNFα
dex 0.25
TNFα
dex 0.25
TNFα
dex 1.0
TNFα
kDa
M
70
55

## Slide 81
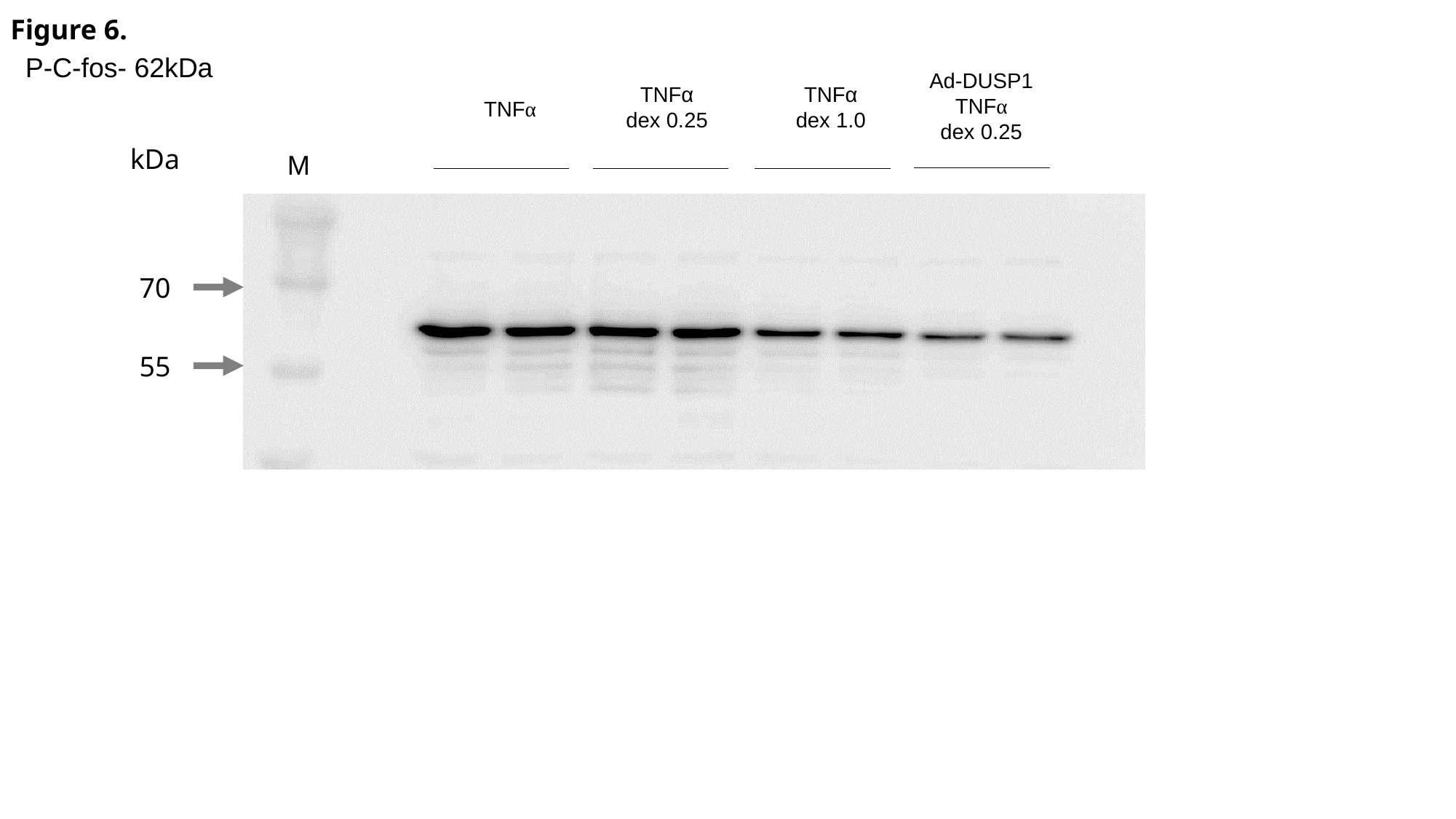

Figure 6.
P-C-fos- 62kDa
Ad-DUSP1
TNFα
dex 0.25
TNFα
dex 0.25
TNFα
dex 1.0
TNFα
kDa
M
70
55

## Slide 82
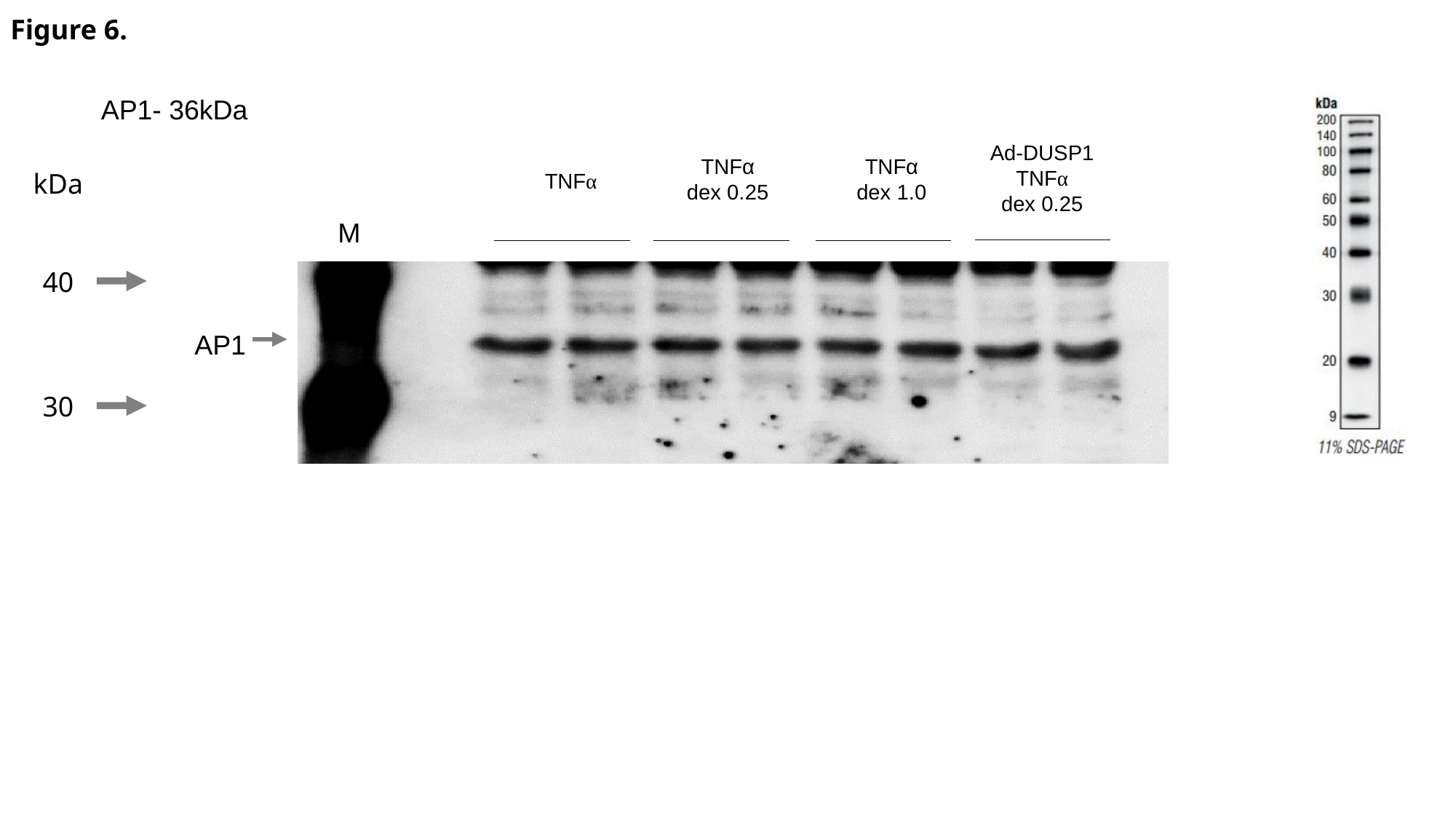

Figure 6.
AP1- 36kDa
Ad-DUSP1
TNFα
dex 0.25
TNFα
dex 0.25
TNFα
dex 1.0
kDa
TNFα
M
40
AP1
30

## Slide 83
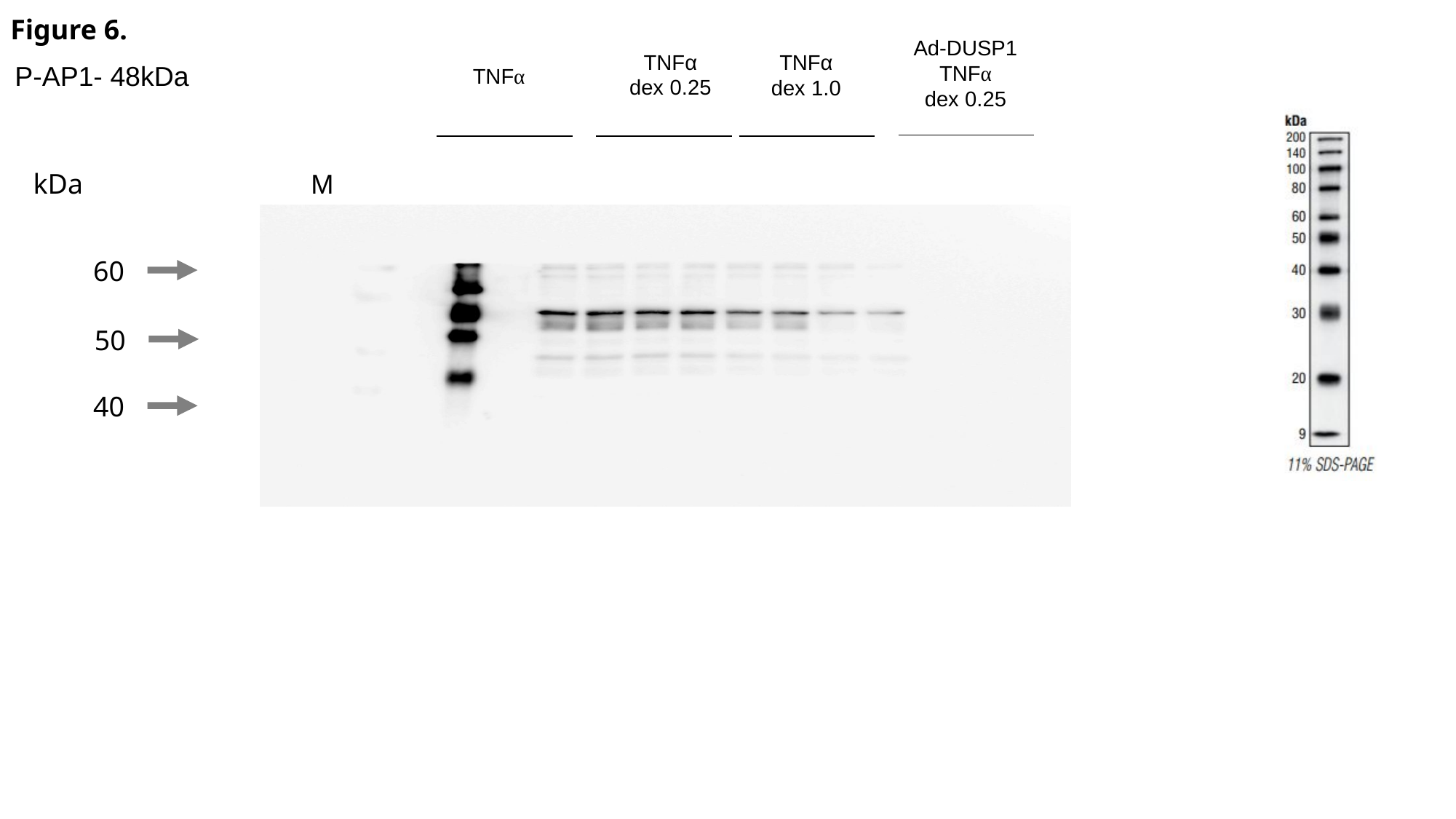

Figure 6.
Ad-DUSP1
TNFα
dex 0.25
TNFα
dex 0.25
TNFα
dex 1.0
P-AP1- 48kDa
TNFα
kDa
M
60
50
40

## Slide 84
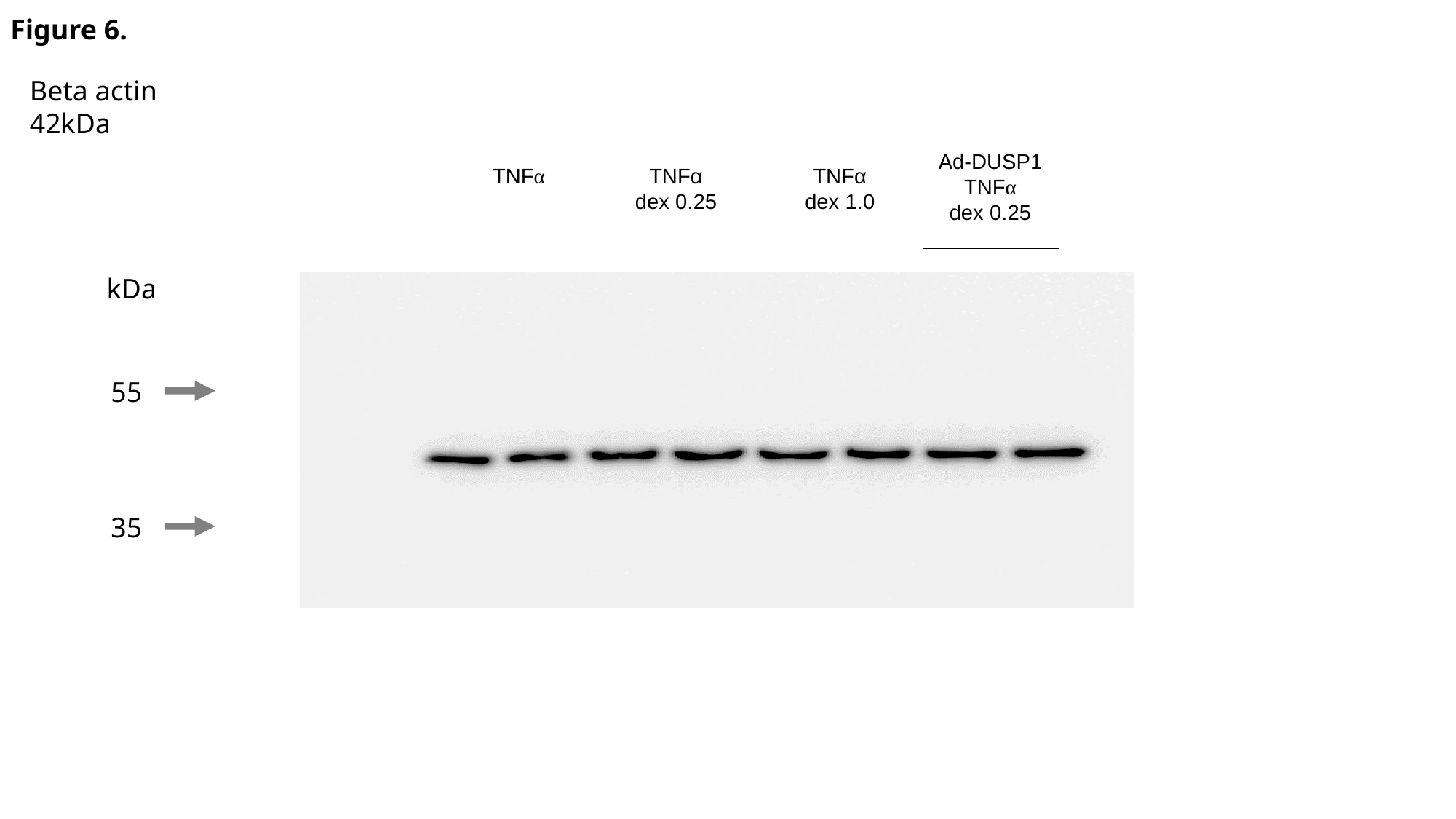

Figure 6.
Beta actin
42kDa
Ad-DUSP1
TNFα
dex 0.25
TNFα
TNFα
dex 0.25
TNFα
dex 1.0
kDa
55
35

## Slide 85
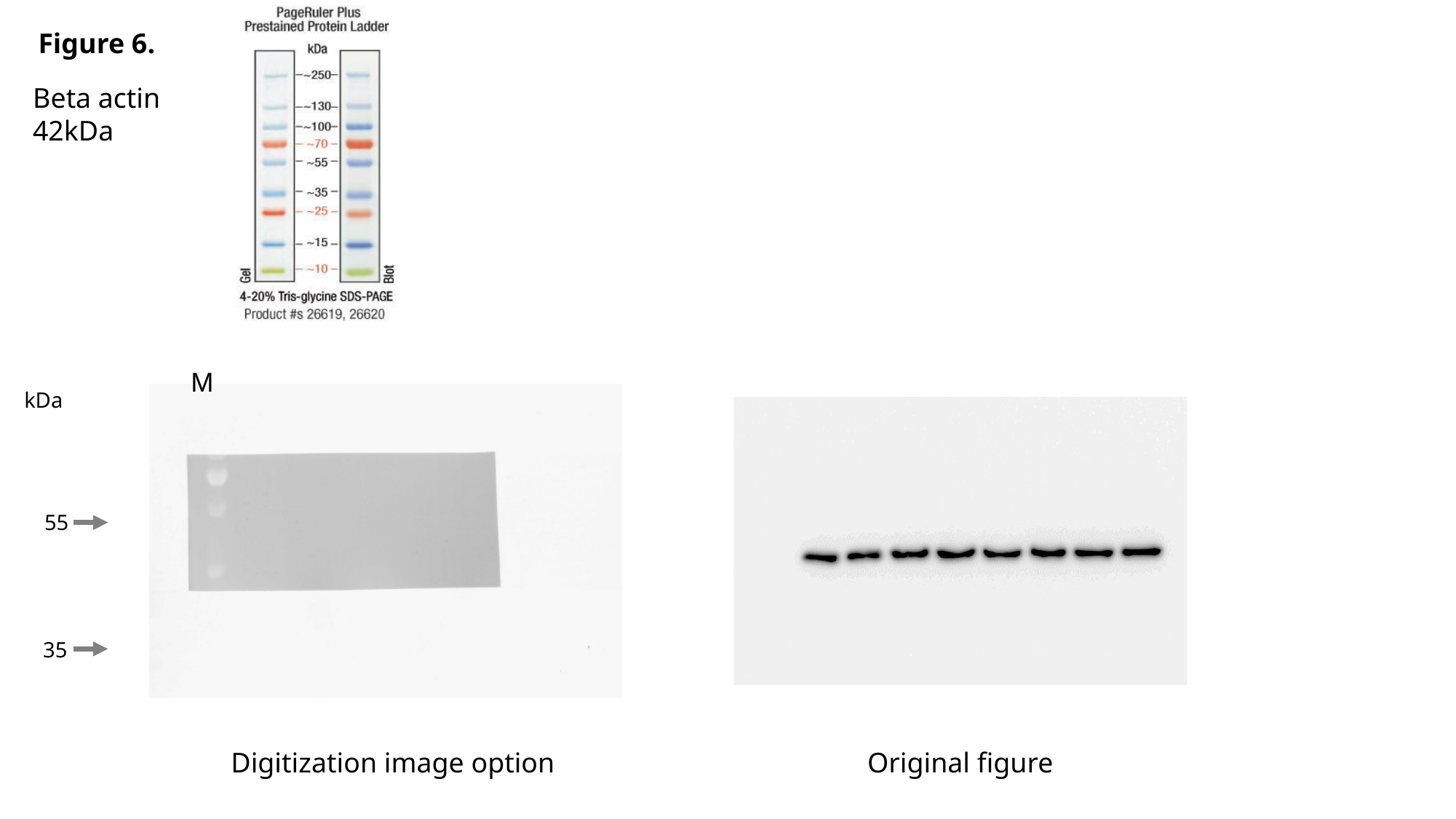

Figure 6.
Beta actin
42kDa
M
kDa
55
35
Digitization image option
Original figure
