# Supplementary material for: RNA‐seq profiling of tubulointerstitial tissue reveals a potential therapeutic role of dual anti‐phosphatase 1 in glomerulonephritis
Source: J Cell Mol Med. 2022 Apr 29;26(12):3364–77. doi: 10.1111/jcmm.17340 (PMC9189340; doi:10.1111/jcmm.17340)
Supplement: Supplementary file 4 — Supplementary Material [file JCMM-26-3364-s004.pdf]

Online Supplemental Materials

RNA-seq profiling of tubulointerstitial tissue reveals a potential therapeutic role of dual anti-phosphatase 1 in glomerulonephritis

Park S, Lee H et al.

Table of contents

Supplemental Table 1. Differentially expressed genes (.xlsx).

Supplemental Table 2. Gene ontology annotation results of differentially expressed genes with false discovery rate < 0.05 and absolute log2 fold change ≥ 1 (xlsx).

Supplemental Figure 1. Principal component analysis results and expression of structure-specific genes.

Supplemental Figure 2. Experiment to investigate the appropriate BCI concentration.

Supplemental Figure 3. Experiment to investigate the appropriate MOI of adeno-DUSP1 for DUSP1 overexpression.

Supplemental Figure 4. Experiment to investigate dexamethasone dosage. We assessed phospho-p38 and DUSP1 levels in hTECs treated with 0.25, 0.5, and 1.0 mM dexamethasone for 1 hour and then stimulated with TNFα.

Supplemental Figure 1. Principal component analysis results and expression of structure-specific genes.

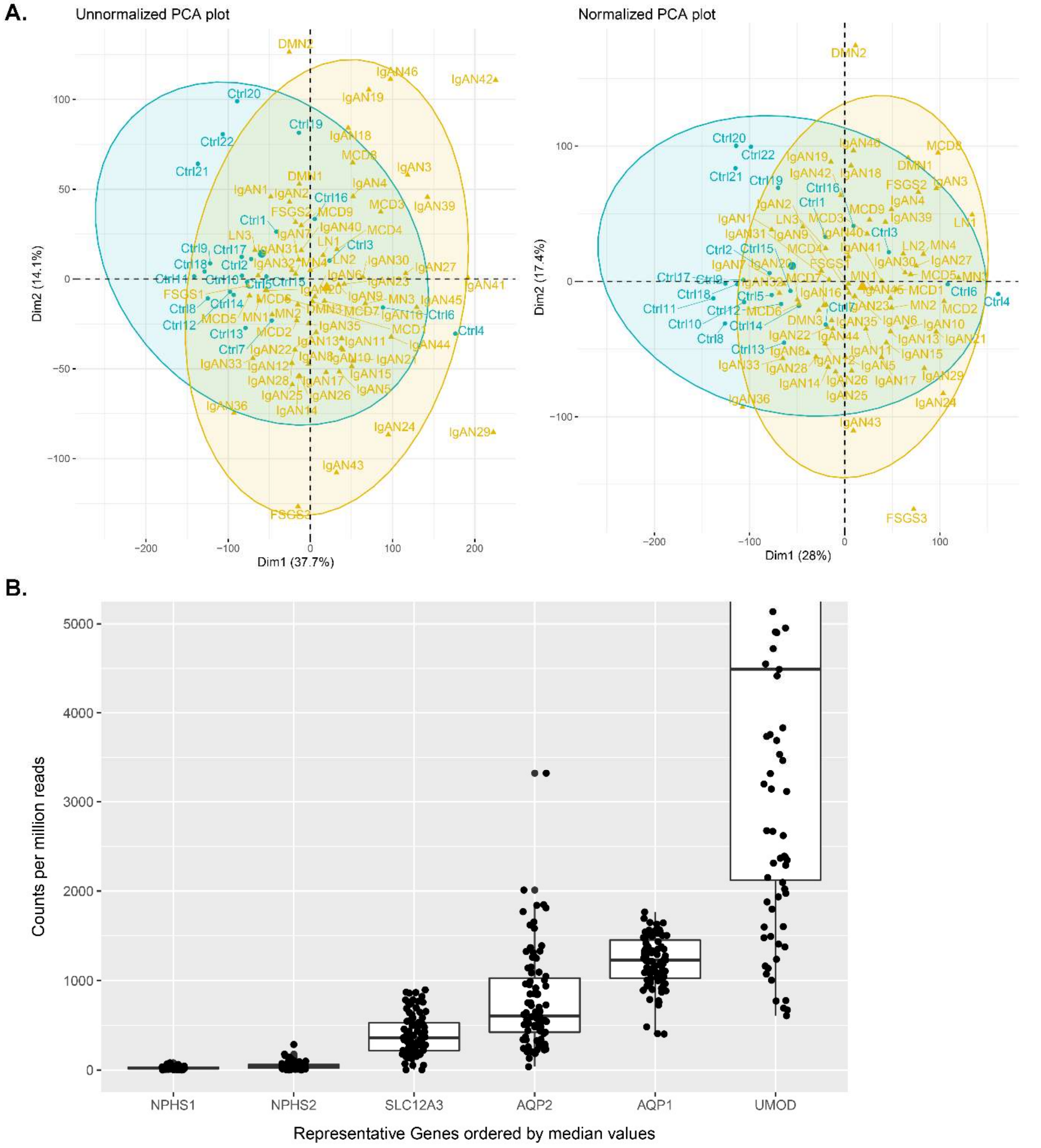

A: Principal components analysis with unnormalized and normalized read counts were performed and plots were drawn by the first and second principal components. B: The counts per million reads values of structure-specific genes expressions. The expressions of glomerulus-specific NPHS1 or NPHS2 were markedly low in the manually dissected tubule tissues, suggesting that the microdissection procedure successfully isolated tubules and glomerulus.

Supplemental Figure 2. Experiment to investigate the appropriate BCI concentration.

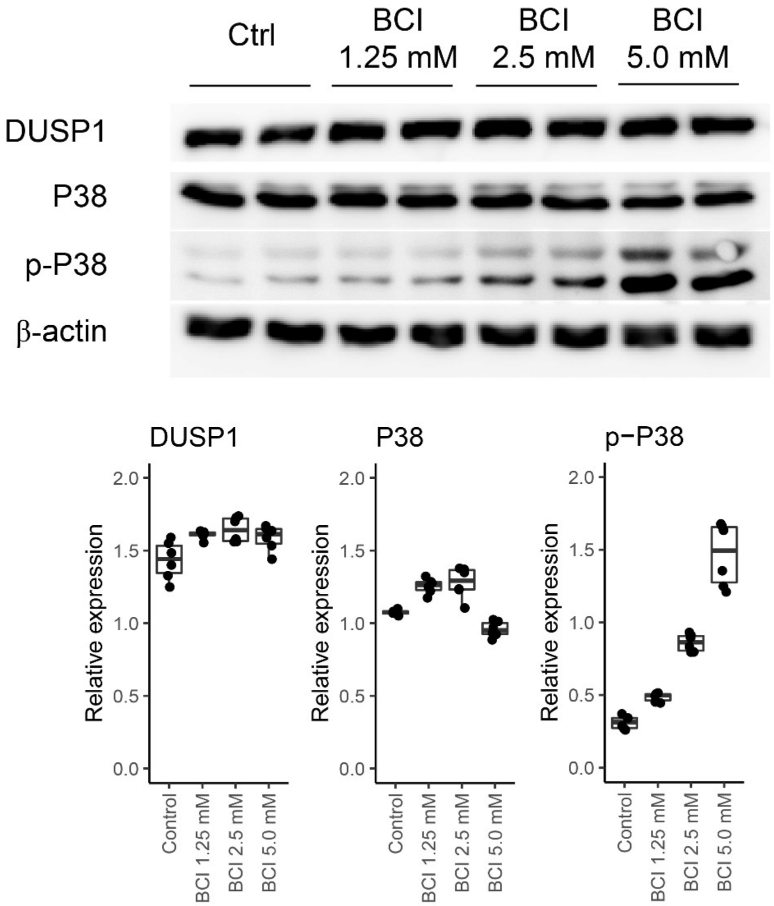

We performed the experiment by adding BCI at 1.25 mM, 2.5 mM, and 5.0 mM to the culture medium of hTECs for 3 hours and then stimulating the hTECs with TNFα. The BCI concentration 5.0 mM was considered to sufficiently increase p38 expression was used for the main experiment.

Supplemental Figure 3. Experiment to investigate the appropriate MOI of adeno-DUSP1 for DUSP1 overexpression.

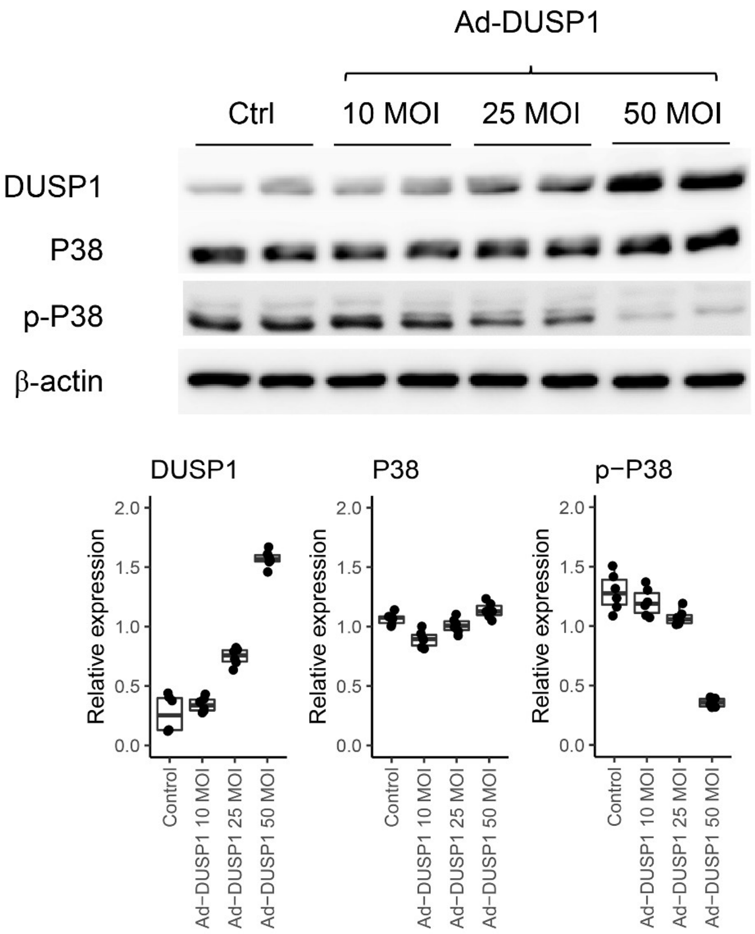

We confirmed the appropriate multiplicity of infection (MOI) for *DUSP1* overexpression by transducing cells with adeno-DUSP1 at MOIs of 10, 25, and 50 for 24 hours and assessing phospho-p38 and DUSP1 levels.

Supplemental Figure 4. Experiment to investigate dexamethasone dosage.

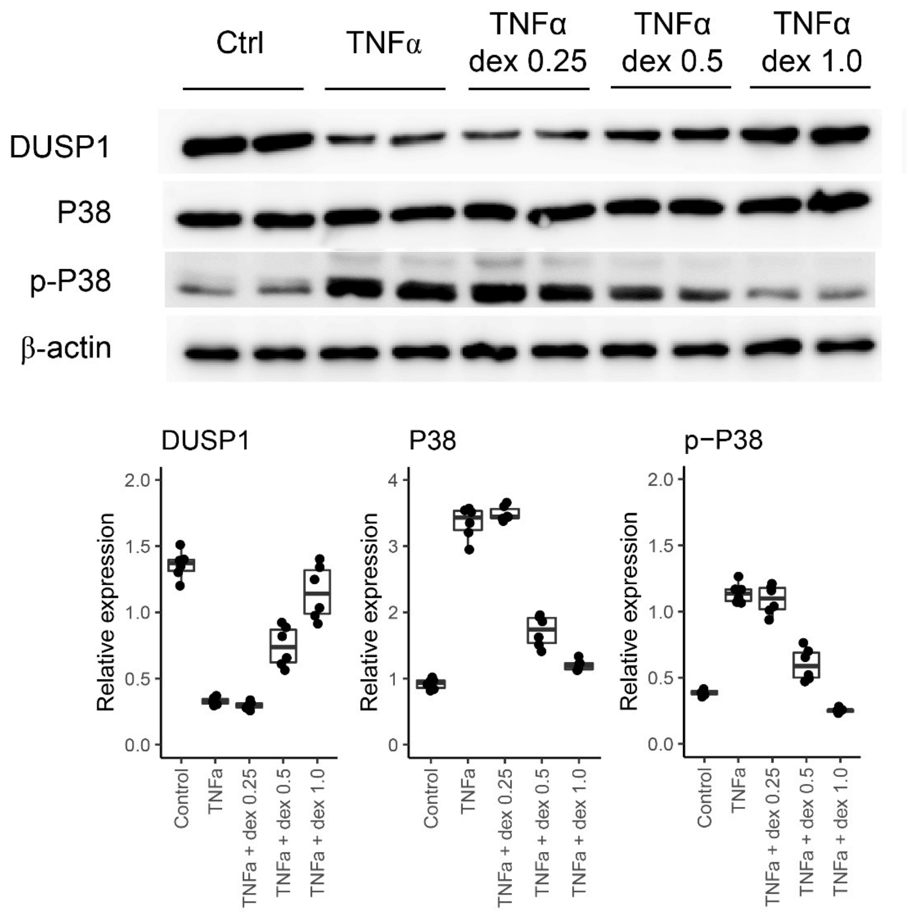

We assessed phospho-p38 and DUSP1 levels in hTECs treated with 0.25, 0.5, and 1.0 mM dexamethasone for 1 hour and then stimulated with TNFα.
